# SEASIM-NEAM: A Spatially-Explicit Agent-based SIMulator of North East Atlantic Mackerel population dynamics

**DOI:** 10.1016/j.mex.2020.101044

**Published:** 2020-08-29

**Authors:** Robin Boyd, Nicola Walker, Kieran Hyder, Robert Thorpe, Shovonlal Roy, Richard Sibly

**Affiliations:** a Centre for Ecology and Hydrology, Wallingford, United Kingdom; b Centre for Environment, Fisheries and Aquaculture, Science, Lowestoft, United Kingdom; c School of Environmental Sciences, University of East Anglia, Norfolk, United Kingdom; d Department of Geography and Environmental Science, University of Reading, Reading, United Kingdom; e School of Biological Sciences, University of Reading, Reading, United Kingdom

**Keywords:** Atlantic mackerel, Individual-based model, Bioenergetics, Spatially-explicit, Approximate bayesian computation

## Abstract

In 2018 we published a spatially-explicit individual-based model (IBM) that uses satellite-derived maps of food availability and temperature to predict Northeast Atlantic mackerel (Scomber scombrus, NEAM) population dynamics. Since then, to address various ecological questions, we have extended the IBM to include additional processes and data. Throughout its development, technical documents have been provided in the form of e.g. supplementary information to published articles. However, we acknowledge that it would be difficult for potential users to collate information from separate supplementary documents and gain a full understanding of the current state of the IBM. Here, we provide a full technical specification of the latest version of our IBM. The technical specification is provided in the standard ODD (Overview, Design concepts and Details) format, and supplemented by a TRACE (TRAnsparent and Comprehensive model Evaludation) document. For the first time, we give our model the acronym SEASIM-NEAM: a Spatially-Explicit Agent-based SIMulator of North East Atlantic Mackerel population dynamics. This article supersedes previous documentation. Going forward we hope that this article will stimulate development of similar models.

• This article collates improvements that have been made to SEASIM-NEAM over time.

## Graphical abstract

**Figure:**
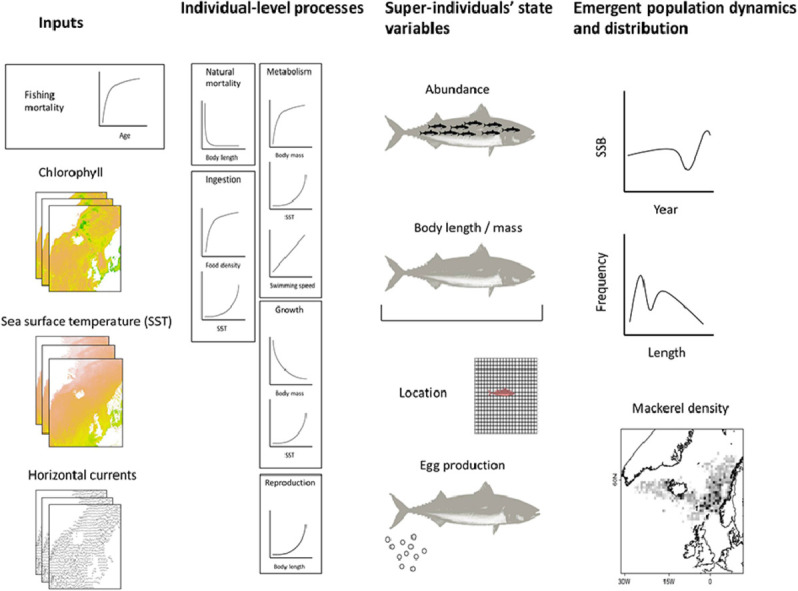

**Table:** Specifications Table

Subject Area	Environmental Science
More specific subject area	Fisheries ecology
Method name	SEASIM-NEAM
Name and reference of original method	Boyd, R., S. Roy, R. Sibly, R. Thorpe, and K. Hyder. 2018. A general approach to incorporating spatial and temporal variation in individual-based models of fish populations with application to Atlantic mackerel. Ecological Modelling 382:9–17.Boyd, R., R. M. Sibly, K. Hyder, R. B. Thorpe, N. D. Walker, and S. Roy. 2020. Simulating the summer feeding distribution of Northeast Atlantic mackerel with a mechanistic individual-based model. Progress in Oceanography 183:102299.Boyd R, Thorpe R, Hyder K, Roy S, Walker N and Sibly R (2020) Potential Consequences of Climate and Management Scenarios for the Northeast Atlantic Mackerel Fishery. Front. Mar. Sci. 7:639. doi: 10.3389/fmars.2020.00639
Resource availability	Model code - https://github.com/robboyd/SEASIM-NEAM/tree/masterR Markdown documents showing how to run and calibrate the model provided in supplementary material.Supporting “TRAnsparent and Comprehensive model Evaludation” (TRACE) document provided in the supplementary material.Input data for the model is very large. We are happy to provide this data to anyone who can provide a means to transfer it.

## Method details

### Model description

In this article, we provide a technical specification of SEASIM-NEAM in the standard ODD (Overview, Design Concepts and Details) format [26]. See [6], [7] and [8] for earlier applications of the SEASIM-NEAM. We refer the reader to the supplementary TRACE (TRAnsparent and Comprehensive model Evaludation) document throughout, where full details of the IBM's calibration, validation, sensitivity analyses etc. can be found. SEASIM-NEAM was built in the open-source software NetLogo [83], where it comes with an easy-to-use GUI, but can also be run from the R statistical environment [59] using the RNetLogo package [73]. The Netlogo and R code can be found at https://github.com/robboyd/SEASIM-NEAM/tree/master.

### Purpose and patterns

The primary goal of SEASIM-NEAM is prediction: it is designed to assess how the Northeast Atlantic mackerel (NEAM) stock may respond to various climate and management (fishing) scenarios. Specifically, the model predicts how e.g. NEAM Spawning Stock Biomass (SSB, biomass of mature individuals), average body weights-at-age and spatial distribution (density and presence/ absence) may respond to spatial and temporal variations in prey availability, temperature and exploitation.

### Model overview

**Fig. 1:**
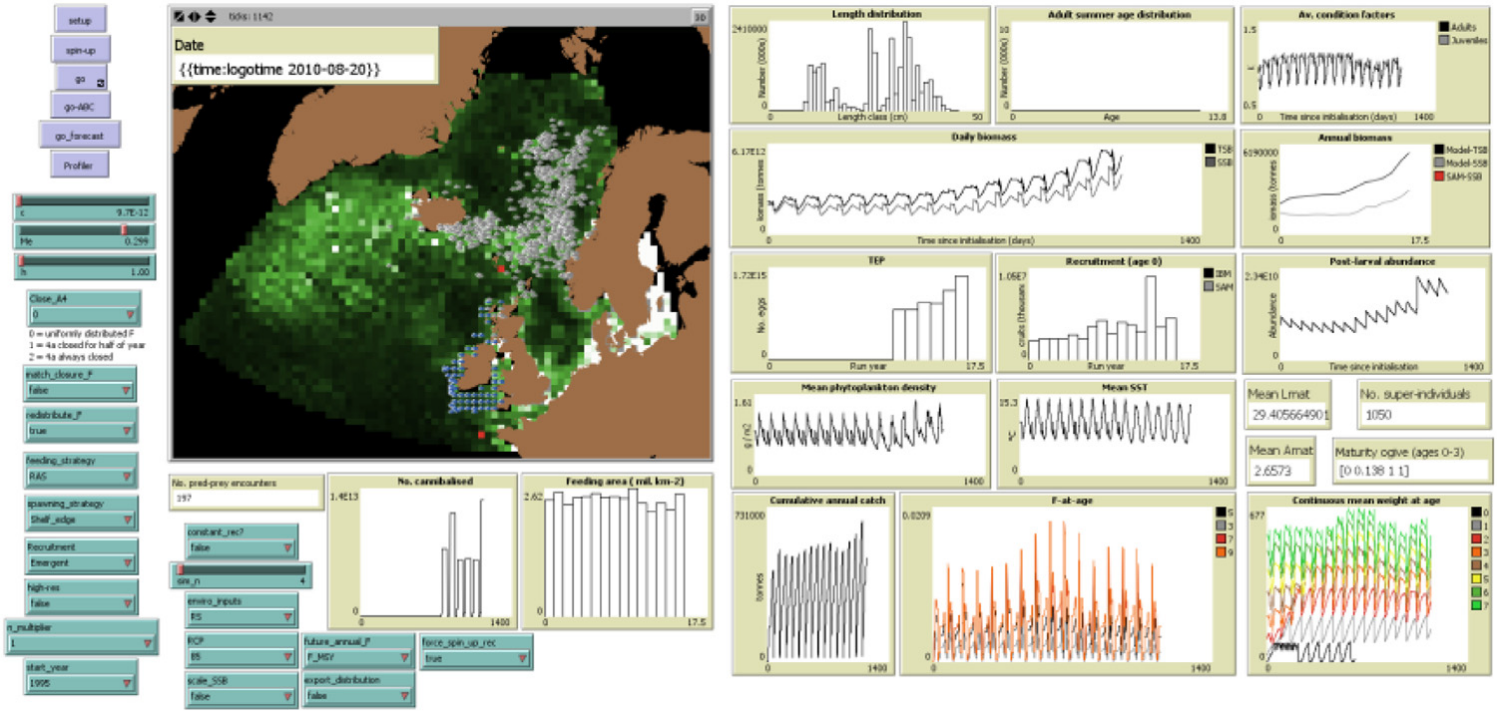
SEASIM-NEAM's GUI. There are there are three types of NetLogo widgets shown: Grey buttons (used to initialise and run the model); green “sliders”, used to select input values from a specified range; green “choosers” allowing users to select configuration options from drop down menus; and brown “plots and monitors” displaying e.g. predicted population dynamics. See TRACE section 5 for full details of the interface widgets. Grey SIs in the Nordic seas are adults, and the blue SIs to the west of the British Isles are juveniles. The red cells indicate “destination” patches towards which adults migrate. The southerly red cell is the destination for the spawning migration, and the northerly red cell is the destination for the feeding and return overwintering migrations (see text for details). The colour of the landscape corresponds to phytoplankton density: black indicates low density, through green and then white which indicates high density. The colour bins are arbitrary.

The model seascape comprises dynamic maps of phytoplankton density, which is used as a proxy for baseline prey availability (Fig. 1), Sea Surface Temperature (SST), photoperiod and horizontal current velocities. The fish population represents the largest spawning unit of the NEAM stock, the western component, which has historically comprised ~80% of the stock's total biomass [32,^33^,35]. It should be noted that there is evidence of straying between the western and the much smaller North Sea spawning component of NEAM [41], which is not accounted for in the IBM. Fish are grouped into super-individuals (SIs), which comprise a number of individuals with identical variables [63]. SIs are sometimes considered to represent schools of identical individuals in varying abundances [66], but the approach is mainly used for computational tractability. Each year, at fixed times, SIs migrate between spawning, feeding and overwintering areas. After reaching the destination areas they then move locally until the next migration is triggered. Each SI has an energy budget which determines how its state variables (e.g. body length, body mass, energy stores) change in response to its local environment. Age-specific rates of fishing mortality from the NEAM stock assessment determine the number of individuals removed from the population due to harvesting [37]. A constant number of new SIs enter the model as eggs each year, but the abundance that they represent on entry reflects the amount of energy the spawning stock was able to accumulate for egg production prior to spawning. SIs’ abundances are reduced as fishing and natural mortalities are applied each time-step. Population-level outputs are by summarising the characteristics of the SIs including their abundances. For example, biomass is the sum of individual body masses, and spatial distribution is a summary of the individuals’ locations.

**Fig. 2:**
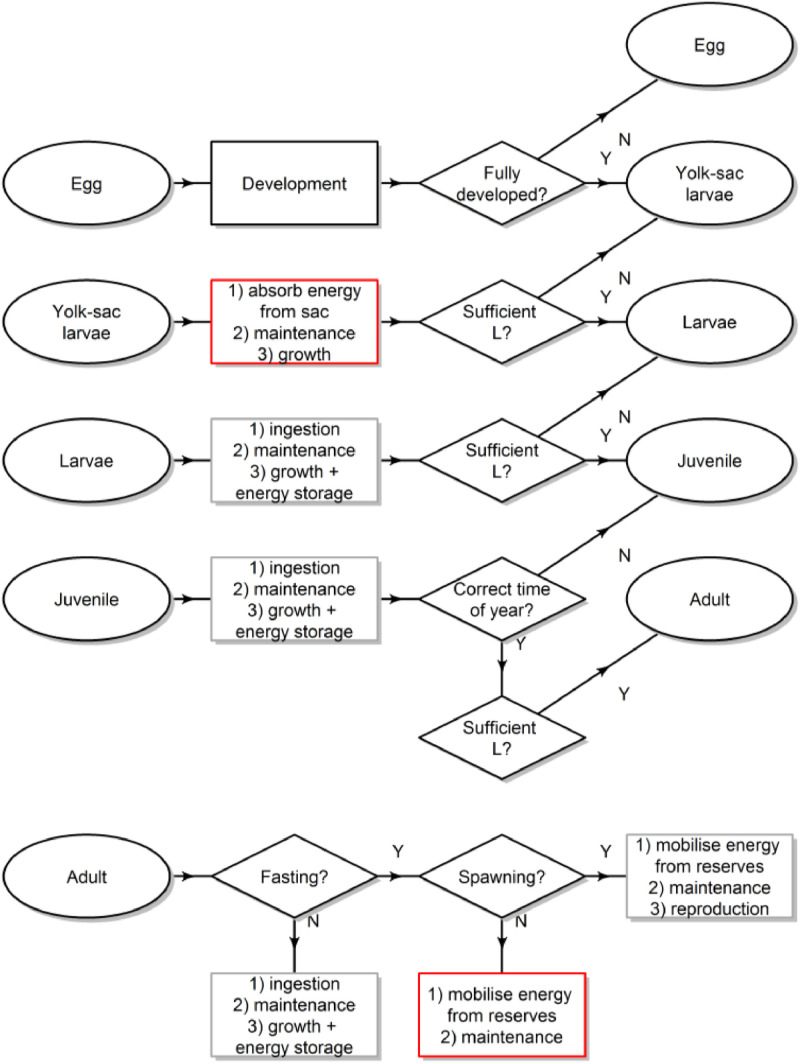
Conceptual model showing the key bioenergetics processes that individuals of different life stages implement between time t and t+1, and the conditions required for progression to the next life stage. Red boxes indicate an effect of SST, and grey boxes an effect of SST and food availability. L is body length.

### State Variables and Scales

The IBM seascape comprises a two-dimensional grid of 60 km^2^ patches representing the sea surface (Fig. 1). The geographical extent spans from 47 to 77°N, and from -45° to 20°E. Each grid cell is characterised by prey density, SST, NEAM density, photoperiod (proportion of day length) and horizontal current velocities in x and y dimensions. The NEAM population is represented by a roughly constant number of SIs; as n_cohort_ new SIs enter the model as eggs each year, an equal number reach 15 years of age and are removed from the model. Slight variations in the number of SIs arise throughout simulations because 1) a SI is removed from the model if its abundance < 1 (this is rare). Users can select the number of SIs in multiples of 1050, which is equivalent to choosing n_cohort_ in multiples of 70 (as 70 * number of age groups (15) = 1050). While the number of SIs remains approximately constant, the abundance that they represent differs: A SI's abundance is determined by the level of egg production in the year that it entered the model, and all subsequent mortality. Each SI is characterised by a number of state variables (see Table 1). The spawning area comprises patches on the continental shelf edge to the west of the British Isles (-550 m < depth < -50 m; fig. 1) on which 10°C < SST < 14°C [65]. The feeding area is a fully emergent feature of the IBM and is not constrained geographically. The overwintering area is assumed to be ICES divisions 6a (west of Scotland) and 4a (northern North Sea, Fig. 5). The nursery area includes all patches that are ≤ 200 m deep [42] to the west of the British Isles (< 4°00 west). The temporal extent of the IBM depends on the choice of input data; where Earth System Model (ESM) derived inputs are used the model extent may span 1981 to 2050 (chosen by the user), but if satellite-derived inputs are used the temporal extent is fixed at 2005 to 2018. The IBM proceeds in discrete five-day time-steps.

**Table 1:** Key state variables charcterising SIs and patches. Here we define state variables as variables that cannot be immediately deduced from the state variables of the other entities [60]. As such this table does not include „rate“ variables (e.g. growth, metabolic rate) which could be calculated from e.g. body size and temperature.

State variable	Description	Details
Super-individuals		
Abundance	Number of “actual“ individuals represented by SI	
Age		years/ days
A_mat_	Age at which sexual maturity was reached	Years
Batches	Cumulative number of egg batches spawned in a season	Used to determine when spawning should cease (when >= 5)
Breed	Life stage	Egg, yolk-sac larvae, larvae, juvenile or adult
Development	Number of days developed as an egg	
Energy-reserve	Energy stored as lipid	kJ
F	Fishing mortality rate	Day^−1^
Feeding	Whether or not the individual is feeding (only half of year for adults)	Boolean
Gender		
L	Body length	Cm
L_mat_	Length at which sexual maturity was reached	Cm
M	Total body mass	g
M_gon_	Gonad mass	g
Migrating	Whether or not individual is currently migrating	Boolean
M_standard_	Standard body mass	g
M_struct_	Structural body mass	g
Prey-choices	Potential prey (sufficiently small and on same patch as focal individual)	Netlogo ID numbers
f_r_	Realised fecundity	Proportion of potential fecundity
V_r_	Realised swimming velocity	Minimum velocity plus random noise (km hour^−1^)
Spawning	Whether or not an individual is spawning	Boolean
V_min_	Minimum swimming velocity	Km hour^−1^
x	x coordinate	Floating point
y	y coordinate	Floating point
Patches		
A4	In ICES division 4a?	Boolean
A5	In ICES division 5a?	Boolean
A6	In ICES division 6a?	Boolean
Depth		m
Feed-dist	Distance from destination at the entrance to the feeding area	No. Patches
Latitude		Decimal degrees
Longitude		Decimal degrees
NArea	In nursery area?	Boolean
Ocean	In ocean?	Boolean
OWArea	In overwintering area?	Boolean
p_photo_	Photoperiod	Proportion of 24 h
Rectangle	ICES rectangle	
Ricker-spawn-area	Area designated as spawning grounds for Ricker model	Used as area over which to calculate mean SST for use in Ricker recruitment model (not the default recruitment configuration, see TRACE section 3)
SArea	In spawning area?	Boolean
Shelf-edge	On the European continental shelf edge?	-550 m < depth < -50 m
Spawn-dist	Distance from destination at end of spawning migration	No. Patches
SST	Sea Surface temperature	°C
True-north	Heading equal to true north on the Netlogo grid	Used to calculate effects of horizontal currents in the NetLogo grid
True-west	Heading approximately equal to true west on the Netlogo grid	Used to calculate effects of horizontal currents in the NetLogo grid
U	Zonal component of current velocity	km hour^−1^
V	Meridional component of current velocity	km hour^−1^
X	Phytoplankton biomass	g m^−2^

### Processes, Overview and Scheduling

Full details of the model processes are given in the Submodels sections indicated in parentheses here. The order in which individuals or patches carry out a given process is random. State variables are updated immediately after being calculated by a process. The order in which processes are implemented each time-step are as follows:

1. Phytoplankton and SST data are updated if appropriate (i.e. every tenth day)
2. If it is the first time-step in a month, then photoperiod, current velocities and the proportion of annual fishing mortality that should be applied in that month are updated
3. If it is the first time-step in a year, then annual rates of fishing mortality-at-age are updated
4. Fishing, starvation and background mortalities are applied to SIs (mortality)
5. SIs move to a new location (movement)
6. SIs update their energy budgets (with the exception of reproduction which comes later; energy budget Figure 2)
7. SIs progress to the next life stage if body size thresholds are met (it must also be February 1st for juveniles to reach sexual maturity)
8. If it is the start of the spawning period (March 1st), adults calculate their potential fecundity and the associated energy cost (energy budget)
9. If in the spawning period, adults implement a spawning module. This includes allocating energy to the production of egg batches, spawning those batches at specified intervals, and moving northward as suitably warm regions open up for egg development in the north
10. New SIs enter the model at the egg stage, and calculate their development
11. All SIs age by Δt (days post-hatch)
12. SIs‘ state variables are recorded for analysis outside of the IBM

### Design Concepts

#### Emergence

Movement and bioenergetics models describe the ways in which SIs’ characteristics (e.g. body mass, energy reserves and location) respond to their local food availability and SST. By summarising the characteristics of all the SIs, population measures can be obtained. For example, SSB can be obtained by summing the individual body masses of all adults, and spatial distribution by summarising the locations of the individuals.

#### Sensing

To direct movement individuals can sense the plankton biomass, SST, depth and area type of all patches, and the global variables that indicate when migrations and spawning should begin. To select prey, SIs can sense one another‘s body length and ID number. In order for density dependence to act an ingestion rates and perceptions of patch profitability (see Movement), SIs can sense the density of mackerel on all patches.

#### Interaction

Larger individuals can feed on smaller ones, inflicting predation mortality on them and hence depleting fish prey. Individuals on the same patch also compete with each other for baseline prey (proxied by phytoplankton) according to a competition term in Eq. (2).

#### Stochasticity

There are several stochatic elements to the IBM. If not migrating or actively foraging over summer, individuals move randomly to patches within their search radius (see Movement) and with suitable environmental conditions. Swimming velocity when feeding is given by a minimum swimming velocity plus some random noise (see Movement). In the gradient area search (GAS) foraging model, half of each day is spent moving in a random direction. If multiple potential mackerel prey SIs are available, one is selected randomly to be canniblised. At the end of the feeding migration, SIs stop migrating at a randomly selected distance from their target patch at the entrance to the feeding grounds (see Movement). In the spin-up period, recruits enter the model at the end of each year at body length L_1_ (Table 4) minus some random noise.

#### Observation

During simulations the state variables of all, or a subset, of the SIs can be extracted and summarised to obtain measures of population dynamics and spatial distribution. Key model outputs are summarised in Table 2.

**Table 2:** Population metrics obtained by summarising the characteristics of the individuals, and the dates on which they are extracted.

Metric	Date extracted	Details
SSB in summer	August 1st	Sum of adult body masses
SSB at spawning time	May 1st	Sum of adult body masses
Egg production	June 1st	Number of eggs produced by the spawning stock
Recruitment	December 31st	Number of young-of-the-year surviving until December 31st
Maturity ogives	February 10th	Proportion mature-at-age
Adult summer age distribution	August 1st	Relative age distribution (years) of adults
Weight of 36 cm individuals	Mean each month	Mean body mass of individuals in the 36 cm length group. This length class was chosen as data are available for each month of the year
Quarter 1 juvenile length distribution	March 16th	Relative body length distribution of juveniles
Quarter 4 juvenile length distribution	November 23rd	Relative body length distribution of juveniles
Mean weight-at-age in summer	August 1st	Mean body mass in each age group
Mean weight-at-age at spawning time	May 1st	Mean body mass in each age group
Presence/ absence in summer	Mean over July/ August	Whether or not individuals were present on each patch
Density in summer	Mean over July/ August	Density of individuals present on each patch

**Table 3:** Parameters and their values used in the model. All normalizing and rate constants are shown in units of 1/day and are adjusted for the time-step in the IBM.

Parameter	Symbol	Value	Units	Reference	Details
Taxon-specific normalization constant (AMR)	a_AMR_	8.86 × 10^7^		[16]	See TRACE section 2.3
Assimilation efficiency	A_e_	0.95	Proportion of ingested energy	[46]	Proportion of ingested energy made available to the energy budget
Normalizing constant for fecundity	a_f_	8.80		[48]	Normalizing constant for fecundity-length relationship
Caudal fin aspect ratio	A_r_	4.01		FishBase	
Taxon-specific normalization constant (SMR)	a_SMR_	0.45 × 10^8^		[29]	See TRACE section 2.3
Swimming speed normalizing constant	a_v_	0.15		[62]	Normalizing constant for speed-length relationship
Exponent for the scaling of AMR with body mass	b_AMR_	0.75		[29]	See TRACE section 2.3
Scaling exponent for fecundity	b_f_	3.02		[48]	Exponent in fecundity-length relationship
Exponent for the scaling of SMR with body mass	b_SMR_	0.75		[16]	See TRACE section 2.3
Exponent for scaling of swimming velocity with body length	b_v_	0.62		[62]	
Strength of the predator density dependence	c	9.71 × 10^−11^		This study	Estimated with ABC (see TRACE section 2.3)
Exponent for the scaling of AMR with swimming speed	c_AMR_	1		[16]	See TRACE section 2.3
Maximum consumption rate	C_max_	0.69	g g^−1^ day^−1^	[28]	See TRACE section 2.3
Exponent for scaling of swimming velocity with caudal fin aspect ratio	c_v_	0.35		[62]	
Activation energy	E_a_	0.5	eV	[22]	Minimum energy required for physiological chemical reactions
Energy content of flesh	E_flesh_	7.00	kJ g^−1^	[53]	
Energy content of lipid	E_lipid_	39.3	kJ g^−1^	[64]	
Maximum energy reserves	E_max_	0.78	Proportion of structural mass	[24]	See TRACE section 2.3
Energy density of phytoplankton	E_phyto_	6.02	kJ g^−1^	[2]	
Energy costs of synthesizing flesh	E_sf_	3.60	kJ g^−1^	[67, 68]	
Energy costs of synthesizing lipid	E_sl_	14.7	kJ g^−1^	[58]	See TRACE section 2.3
Half saturation constant	h	1.26	g m^−2^	This study	Estimated with ABC (see section 2.3). The food density at which ingestion is half maximum at a given temperature
Rate of cannibalism	IR_cannibalism_	0.064	Proportion of ingestion rate	[56]	Only relavent where suitable prey are available. Proportion of ingestion rate comprising mackerel prey.
Growth constant	k	8.6 × 10^−4^	day^−1^	FishBase	See TRACE section 2.3
Boltzmann's constant	K	8.62 × 10^−5^	eV K^−1^		
Maximum growth rate	k_1_	0.025	day^−1^	[79]	See TRACE section 2.3
Maximum length after first growing season	L_1_	20	cm	[79]	
Asymptotic length	L_∞_	42.4	cm	FishBase	See TRACE section 2.3
Length at hatching	L_hatch_	0.3	cm	[79]	
Threshold length for maturity	L_mat_	26.2	cm	FishBase	See TRACE section 2.3
Background adult mortality	M_a_	0.00041	day^−1^	[36]	Constant for all ages, based on tagging studies in 1980’s and used in the stock assessment
Background early mortality	M_e_	0.287	day^−1^	This study	Estimated with ABC (see TRACE section 2.3). Applied to eggs, yolk-sac larvae and larvae
Number of egg batches spawned	n_b_	5	season^−1^	This study	For simplicity, the actual number is around 20
Lower temperature limit	SST_lim_	7	°C	[52]	In summer the threshold SST below which SIs avoid
Age at maximum growth	t_max_	55	days	[79]	

### Initialization

The IBM is initialised on January 1st of a chosen year (1981 onwards) using numbers-at-age in from the latest ICES stock assessment. This population is apportioned in to n SIs assuming a gender ratio of 1:1. Body lengths are calculated from age using the standard von Bertalanffy equation (Eq. 12 here), and energy reserves are set at half maximum. From these all other state variables are calculated when the simulations begin. Adults and juveniles are distributed randomly in the overwintering and nursery areas, respectively (Fig. 5). After initialisation the model spins up for ten years with recruitment forced from the ICES stock assessment. Recruits are introduced at the end of each year, with body length set at the maximum length at the end of the first growing season (first winter), L_1_ (cm), minus ε 3, where ε is drawn randomly from uniform distribution between 0 and 1.

### Input data

The IBM is forced with estimates of fishing mortality at age, chlorophyll concentration (from which we derive phytoplankton biomass with an empirical conversion factor), SST, zonal and meridional horizontal current velocities and photoperiod. See section TRACE section 3 for details of how these data were processed.

#### Fishing mortality

**Fig. 3:**
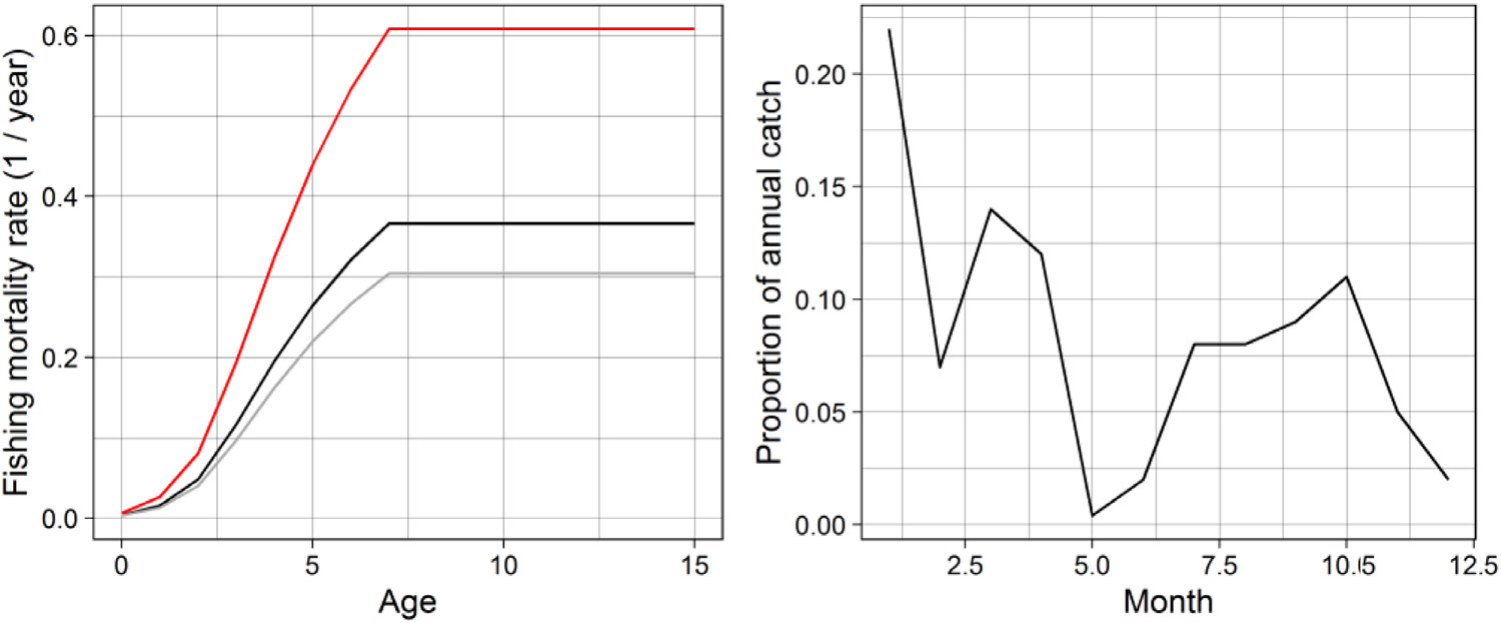
Left panel: Mean historical F at age (2001 to 2018, black line) from which F_MSY_ is calculated with a multiplier of 0.83 (grey line) and F_lim_ with a multiplier of 1.66 (red line). Right panel: Proportion of annual catch in each month over the historical period which is used to apportion F over each year in SEASIM-NEAM.

Historical annual rates of fishing mortality F at age are taken from the 2019 NEAM stock assessment [37]. Unless stated otherwise, F is applied uniformly to all individuals in an age group regardless of their location. We incorporate monthly variation in F by setting the fractional of annual F taken in each month as proportional to the mean historical (2001 to 2018) fraction of the annual NEAM catch taken in each month (Fig. 3).

**Fig. 4:**
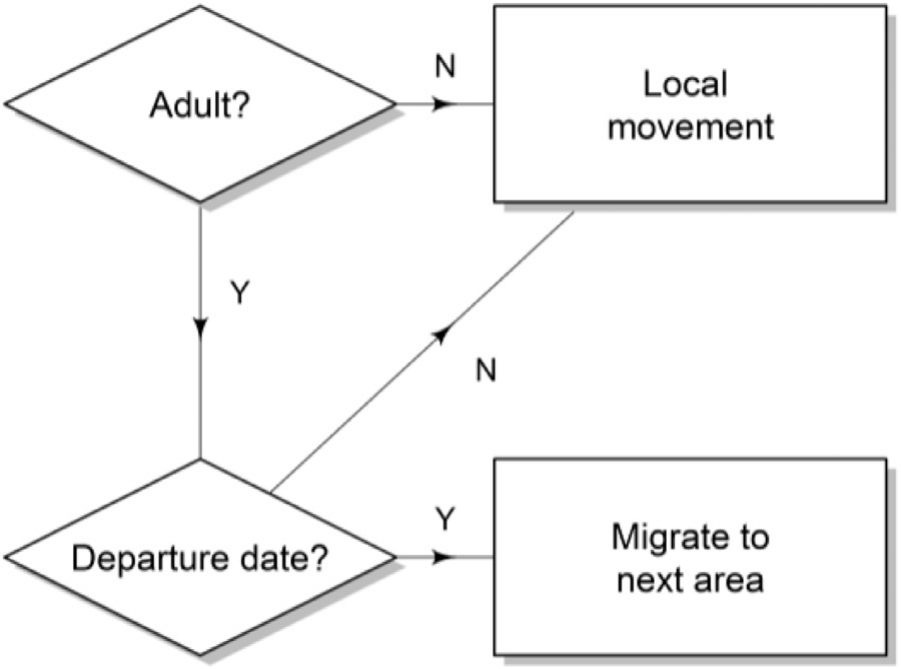
Conceptual movement algorithm. Local movement differs between different areas and at different times (e.g. spawning period and overwintering, see text for full details).

The default future fishing scenario comprises a constant F-at-age at the historical mean level (Fig. 3). Alternatively, users can select one of three multipliers which are used to convert the historical mean F over the most important age groups to the fishery (for NEAM 4-8 years) to one of three rates: F = 0; F_MSY_ (0.23 year^−1^)^,^ i.e. the level of harvesting that is likely to result in maximum sustainable yield in the long-term; and F_lim_ (0.46 year^−1^), i.e. high mortality used as an upper reference point [31,38] (see Fig. 3). Monthly variation in F is implemented as in the historical period.

#### Environmental inputs

Environmental inputs to SEASIM-NEAM include maps of chlorophyll concentration, from which we derive phytoplankton density (with an empirical conversion factor), SST, bathymetry, photoperiod and horizontal current velocities. Users can select chlorophyll and SST estimates derived from satellite remote-sensing, or from the earth system model GFDL-ESM-2M [18]. The satellite-derived inputs comprise ten-day composites and are updated accordingly. It should be noted that when using the satellite-derived inputs the temporal extent of SEASIM-NEAM is fixed at 2005 to 2018. The ESM outputs represent monthly averages. The data required processing for use in SEASIM-NEAM (e.g. re-gridding), the details of which can be found in TRACE section 3. When using the ESM inputs users must choose from one of representative concentration pathways (RCPs) 2.6 or 8.5, representing high and low levels of climate change mitigation action, respectively. Forecasts of the environmental inputs are available out to 2050 for each RCP scenario.

Near surface (average over 0 to -30 m) horizontal current velocities were taken from the 1/3 ⁰ OSCAR dataset [20]. Currents influence the movements of adults over summer (Eq. 4), so we obtained data for the months May through September. Outside of this period current velocities have no effect in SEASIM-NEAM. It would not be appropriate to include the effects of near surface current velocities on individuals outside of the summer period, when mackerel may inhabit deeper waters (e.g. -50 to -220 m over winter) [39]. Over summer NEAM are found in the upper water layer (average of ~ -20 m) [50]. As data are not available for the selected months prior to 2012, we generated mean climatologies for each month over 2012 to 2018. As such we do not account for inter-annual variability in current velocities.

Data on photoperiod (as a proportion of 24 h) at all latitudes in the IBM grid was extracted for each month using the daylength() function in the R package geosphere [30]. Values correspond to the 15th day of each month, and are updated at the start of each month in SEASIM-NEAM.

### Sub-models

Most parameters were derived from the literature as shown in Table 4. Mass is in units of wet weight throughout.

#### Movement

The following sub-models describe the ways in which SIs are directed around the landscape. In broad terms, SIs migrate between different areas (e.g. spawning, nursery, feeding), and otherwise move locally within an area. Migrations are date-triggered. Localised movement differs between area, e.g. local movement when spawning in spring differs from local movement when feeding over summer. At most times of year movement is represented in discrete space, i.e. on a patch-by-patch basis. However, we spent considerable time improving the way in which movement is modelled for adults in the summer period [7], which now operates in continuous space (details below). We hope that in time SEASIM-NEAM will be further developed such that all movement is described in a Lagrangian framework.

#### Migrations

**Fig. 5:**
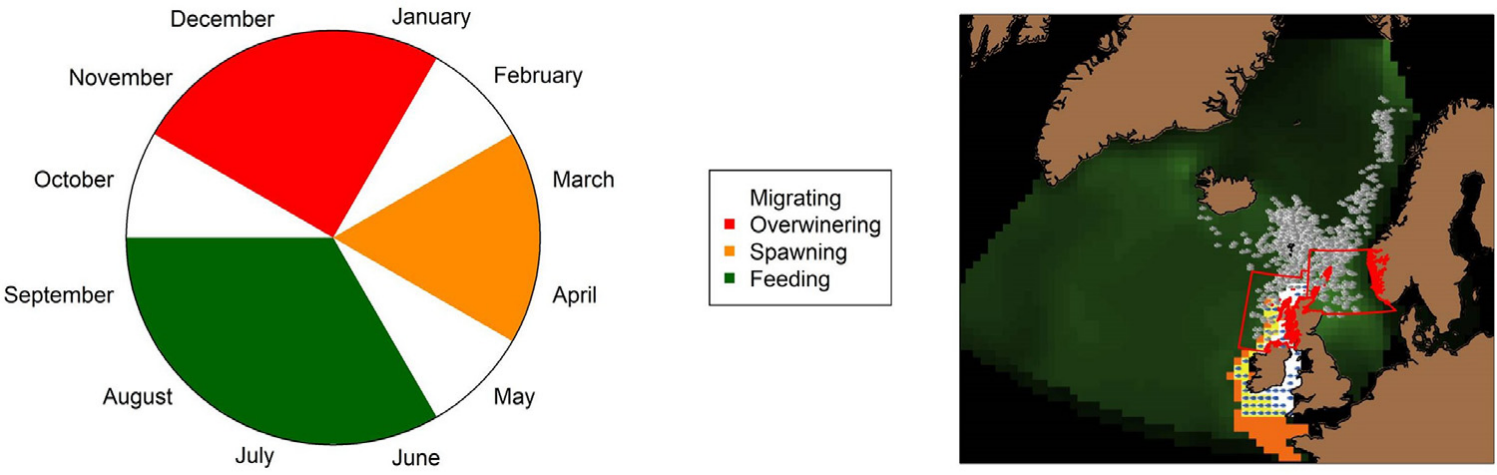
Left panel: Broad summary of adults‘ locations throughout a year. White segments indicate migrations which begin at the start of a month but do not necessarily last the whole month. Right panel: Locations of the spawning (orange cells), overwintering (red boxes) and nursery areas (white cells, yellow cells are both spawning and nursery areas). The adult feeding distribution is not constrained geographically; it is a fully-emergent feature of the IBM.

Adults cycle between overwintering, spawning and feeding areas [75] (see state variables and scales). Migration departure dates were approximated from Uriarte et al. [75] and Petitgas et al. [55] and imposed at: October 1st for the overwintering migration; February 1st for spawning; and May 1st for the feeding migration (Fig. 5). While this scheme captures the general pattern of NEAM migration, it should be noted that in reality migration timings can vary between years [40].

Once a migration is triggered, adults move from their current area towards the relevant destination. For each migration there is a destination patch corresponding to the entrance to the relevant area. The destination for the feeding and return overwintering migrations are at the entrance to the feeding area in the Faroe Shetland channel (northernmost red patch on Fig. 1). For the spawning migration the destination patch is located in the southern region of the spawning area (southernmost red patch on Fig. 1). We give each patch that is not on land an index R corresponding to its distance from the destination patch, while accounting for the fact that individuals cannot move over land. Once a migration is triggered (see Fig. 5 for dates), individuals move towards the appropriate destination to a patch with the lowest R within their possible search area. A SIs‘ search area is calculated from its minimum swimming velocity V_min_ (km hr^−1^) [62]:

(1) $$V_{\text{min}}=a_{v}\;L^{b_{v}}{A_{r}}^{c_{v}}$$

where a_v_ is a normalizing constant, L is body length, A_r_ is the caudal fin aspect ratio, and b_v_ and c_v_ are scaling exponents (see Table 2 for a full list of parameters and TRACE section 8 for a local sensitivity analysis). Eq. 1 allows larger SIs to arrive earliest in destination areas, which has been noted for NEAM [40].

The spawning and feeding migrations are slightly more complicated than the overwintering migration, as they occur primarily along the European shelf edge to the west of the British Isles [10,81]. We represent the shelf edge with a corridor around the British Isles in which -550 m < depth < -50 m (Fig. 1). For the spawning and feeding migrations we then add the constraint that individuals must remain on the shelf edge while moving to minimise R.

#### Local movement

There are three types of local (non-migratory) movement in SEASIM-NEAM: 1) adult foraging in the summer months; 2) random movement constrained to a particular area type (e.g. juveniles in the nursery area, adults in overwintering area in deep winter months); and 3) gradual northward movement on the spawning grounds as suitably warm regions open up for egg development in spring. The details of each type of local movement are given below.

#### Adult foraging

At the end of their feeding migration (Fig. 5), adults begin to move in search of the most profitable locations at which to feed. Each patch is characterised by a profitability cue c_dd_ which is proportional to potential ingestion rate (Eq. 6) in that location. c_dd_ represents the bottom-up effect of phytoplankton density as a proxy for food availability, a density-dependent effect of intraspecific competition, an effect of photoperiod (as NEAM are primarily visual feeders), and an effect of SST (Kelvins), in the form of a Beddington-DeAngelis functional response [4,15]:

(2) $$c_{\text{dd}}=A\left(\text{SST}\right)\;p_{\text{photo}}\;\frac{X}{X+h+\text{cD}}$$

where X is phytoplankton density (g m^−2^), h is a half saturation constant, p_photo_ is photoperiod (as a proportion of 24 h) at the SI's location, D is local mackerel density (g patch^−1^), c determines the strength of the density dependence, and A(SST) is an Arrhenius function giving the effect of SST. A(SST) is given as:

(3) $$A\left(\text{SST}\right)=e^{\frac{-E_{a}}{K}\left(\left(\frac{1}{\text{SST}}\right)-\left(\frac{1}{T_{\text{ref}}}\right)\right)}$$

where E_a_ is an activation energy, K is Boltzmann's constant and T_ref_ is an arbitrary reference temperature.

SIs move in search of the most profitable locations (Eq. 2) at which to feed following a gradient area search (GAS). The GAS algorithm is based on those of Politikos et al. [57], Tu et al. [74] and Boyd et al. [7]. SIs can detect the profitability of the four patches neighbouring their current location. Positions are updated five times per time step (i.e. once per day) to ensure that SIs cannot overshoot the neighbouring patch. Positions in x and y dimensions are updated by:

(4) $$\begin{array}{ccc} x_{t+1} & = & x_{t}+\left(D_{x}+R_{x}+C_{x}\right)\\ y_{t+1} & = & y_{t}+\left(D_{y}+R_{y}+C_{y}\right) \end{array}$$

where D_x_ and D_y_ denote orientated movements towards the most profitable patches, R_x_ and R_y_ denote random movements, and C_x_ and C_y_ are displacements caused by zonal and meridional horizontal currents, respectively.

In the orientated component of Eq. (4) D_x_ and D_y_, SIs make a comparison between the environment at their current location and that of the day before. If it their current location is more profitable, then they maintain the heading of the orientated component of their movement the day before. If their current location is less profitable than the day prior, SIs undertake a gradient search towards what is perceived to be the most profitable neighbouring patch, given by:

(5) $$\begin{array}{ccc} D_{x} & = & V_{r}\;\frac{g_{x}}{\sqrt{{g_{x}}^{2}+{g_{y}}^{2}}}\\ D_{y} & = & V_{r}\;\frac{g_{y}}{\sqrt{{g_{x}}^{2}+{g_{y}}^{2}}} \end{array}$$

where g_x_ and g_y_ are the gradients of the profitability cue (Eqn 2) in x and y dimensions, and V_r_ is realized swimming velocity. V_r_ is equivalent to V_min_ (Eq. 2) plus some random noise, as V_r_ = V_min_ + (V_min_ ε), where ε is drawn randomly from a uniform distribution ranging from zero to one.

Following Politikos et al. [57] we assume that SIs spend half of each day moving in search of the best feeding opportunities (D_x_, D_y_) and half moving in a random direction that is not southward (R_x_, R_y_). Random southward movement is not permitted because acoustic studies have shown that NEAM infrequently swim southwards over summer [50]. However, SIs may still move southward during the oriented component of the GAS algorithm (i.e. if feeding conditions are best on a more southerly patch), or due to currents. R_x_ and R_y_ introduce stochasticity into the GAS models and prevent unrealistic overcrowding on optimal patches.

The effects of horizontal currents on SIs’ locations, C_x_, C_y_, are given as zonal (u) and meridional (v) current velocities (km hour^−1^), respectively, multiplied by the time step (here 24 h as the GAS model operates five times per 5 day time-step).

NEAM avoid areas in which temperature is below 7⁰C [52]. To reflect this, SIs are deterred from moving to patches on which SST is below this threshold. In the directed component of Eq. 4, we repel individuals from patches with SST < 7°C by setting their profitability at zero. For the random component of Eq. 4, if a SI orientates towards a patch on which SST < 7°, its heading is reversed. If currents displace individuals on to an intolerably cold patch (or land) then this movement is abandoned and the SI instead moves to the centroid of the nearest suitable patch.

The energy cost associated with the GAS algorithm is subsumed in to a SI's active metabolic rate (see Maintenance for details).

#### Random movement constrained to particular area types

Movement for adults in the overwintering area, and juveniles in the nursery area, follows a true random walk: They each move to a randomly-selected patch within their possible search area and the same area type each time-step (see migrations and Eq. 1).

#### Spawning movement

Spawning begins on March 1st and lasts for 60 days [19,^43^,82]. This period covers peak spawning in 2007 and 2010 as observed in the triennial mackerel egg survey (MEGS) [34]. Throughout spring NEAM gradually progress northwards towards the feeding area as warming opens up suitable spawning habitat at higher latitudes [14,19]. To reflect this, after spawning a batch of eggs, SIs move to the nearest patch north of their current location on which 10⁰C < SST < 14⁰ C (preferred spawning temperature). If there are no patches northwards in which 10⁰ C < SST < 14⁰C, SIs move to a random neighboring patch within that temperature range.

#### Bioenergetics

The following sub-models describe the energy budgets of larvae, juveniles and adults in terms of individual physiology. We assume eggs and yolk-sac larvae have sufficient energy to satisfy maintenance and maximal growth/ development.

#### Prey availability

SIs can cannibalise other individuals which are: 1) located on the same patch at the same time; 2)  ≥   3.5 times smaller (as in [66]); and 3) < 0.33 cm [3]. If multiple SIs satisfy these conditions, then one is chosen at random to be preyed upon. The energy content of prey depends its fat content (as a proportion of its total mass). Lipid has an energy content E_lipid_ (kJ g^−1^) which is higher than the energy content of structural mass (1- lipid content, E_flesh_). At most times individuals do not overlap with potential mackerel prey and instead eat phytoplankton, which we use as a proxy for baseline food availability, with energy content E_p_ (kJ g^−1^).

#### Ingestion and energy uptake

Adults fast from November until after spawning the following year. Otherwise, ingestion rate IR is given as a function of both predator and prey density. This is described by a Beddington-DeAngelis functional response [4,15], relating IR to body surface area (M^2/3^) [45] and SST (kelvin), as:

(6) $$\text{IR}=A\left(\text{SST}\right)\;C_{\text{max}}\frac{X}{X+h+\text{cD}}M^{2/3}$$

where IR is in g time-step^−1^, C_max_ is the maximum ingestion rate (g time-step^−1^ g^−1^ mackerel), h a the half saturation constant (g m^−2^), X is phytoplankton density (g m^−2^), D is local mackerel density (g patch^−1^) including the density of the focal individual, c determines the strength of predator density dependence and A(SST) is an Arrhenius function giving the effect of SST (Eq. 3). If potential mackerel prey are available (see conditions above), then a proportion of IR, IR_cannibalism_, comprises mackerel prey (see mortality later in this section for the associated predation mortality). A justification for IR_cannibalism_ is provided in TRACE section 3. The remainder of ingested prey, total IR multiplied by (1 – IR_cannibalism_), comprises baseline prey availability as proxied by phytoplankton. Ingestion rate is converted from g time-step^−1^ to kJ time-step^−1^ using the energy content of the relevant prey type (kJ g^−1^). A proportion of ingested energy, an assimilation efficiency A_e_, becomes available for allocation to the following vital processes.

#### Maintenance

Standard metabolic rate, SMR, the level below which an individual cannot survive [21], is used as a baseline measure of maintenance. SMR scales with body mass and temperature, according to:

(7) $$\text{SMR}=a_{\text{SMR}}M^{b_{\text{SMR}}}e^{-E_{a}/K\;\text{SST}}$$

where SMR is measured in kJ time-step^−1^, a_SMR_ is a normalizing constant and $M^{b_{\text{SMR}}}$ is body mass (g) raised to a scaling exponent b_SMR_ (see TRACE section 2.3). SMR is increased to active metabolic rate (AMR, kJ time-step^−1^) when migrating or actively foraging, given by:

(8) $$\text{AMR}=a_{\text{AMR}}M^{b_{\text{AMR}}}V^{c_{\text{AMR}}}e^{-E_{a}/K\;\text{SST}}$$

where a_AMR_ is another normalizing constant and V is swimming velocity (km hr^−1^). For this case study we calculated that AMR scales linearly with V, i.e. an exponent of 1 (see TRACE section 2.3).

#### Growth

S. scombrus growth has a different form and rate in the first growing season than in later life [71,84]. Body length L (cm) at age t (days) in the first growing season is well described by the Gompertz function:

(9) $$L_{t}=L_{1}e^{-e^{-k_{1}\left(t-t_{\text{max}}\right)}}$$

[13,^23^,^69^,79] where L_1_ is the maximum length at the end of the first growing season (cm), k_1_ is the maximum growth rate in the first season, and t_max_ is t (days) at which growth is maximum. k_1_ is adjusted for the SST at which it was recorded using the Arrhenius function. For older individuals the von Bertalanffy equation [5] is generally used:

(10) $$L_{t}=L_{\infty }\left(1-e^{-k\left(t-t_{0}\right)}\right)$$

[78] where k is the Bertalanffy growth constant (time-step^−1^), L_∞_ is the asymptotic length (cm) and t_0_ is an adjustment parameter. k is adjusted for the SST at which it was recorded using the Arrhenius function. Taking the end of the first growth phase to be at t = 240 (days, see TRACE section 2.3), from Eqs. 9 and 10 the maximum growth rate ΔL (cm time-step^−1^) is given by:

(11) $$\Delta L=\left\{ \begin{array}{c} \;k_{1}e^{\frac{-E_{a}}{K}\left(\left(\frac{1}{SST}\right)-\left(\frac{1}{T_{ref}}\right)\right)}\;L\;\ln\left(\frac{L_{1}\;}{L}\right),\;\;t<240\\ \;ke^{\frac{-E_{a}}{K}\left(\left(\frac{1}{SST}\right)-\left(\frac{1}{T_{ref}}\right)\right)}\left(L_{\infty }-L\right),\;\;t\geq 240 \end{array}\right.$$

We assume that adults grow only when feeding [55], i.e. for half of the year. To reflect this, their value of k obtained from Eq. 10 is doubled. ∆L (cm time-step^−1^) is converted to the difference in structural mass ∆M (g time-step^−1^) assuming an allometric relationship between L and structural body mass M_struct_:

(12) $$M_{\text{struct}}=a_{w}L^{b_{w}}$$

where a_w_ is a normalizing constant and b_w_ is a scaling exponent. We define structural mass as total body mass minus lipid stores and gonads. Growth costs are calculated using $\Delta M\;(E_{c}+E_{s})$, where E_c_ is the energy content of flesh (kJ) and E_s_ is the energy costs of synthesising flesh (kJ g^−1^). If insufficient energy is available to support maximum growth, the growth rate is reduced accordingly.

#### Reproduction

The maximum number of eggs that a female can produce, potential fecundity f_p_, is calculated at the beginning of the spawning period (Fig. 5) as a function of body length L, as:

(13) $$f_{p}=a_{f}L^{b_{f}}$$

where a_f_ is a normalizing constant and b_f_ is a scaling exponent. The energy cost of producing a maximum-sized batch of eggs b_max_ (kJ time-step^−1^) is then given as a function of f_p_, as:

(14) $$b_{\text{max}}=\frac{f_{p\;}M_{0}\;\left(E_{c}+E_{s}\right)}{n_{b}}$$

where M_0_ is egg mass, E_c_ is the energy content of flesh, E_s_ is the cost of synthesising tissue and n_b_ is the number of batches produced. NEAM are batch spawners, so energy is allocated to each batch over the inter-batch intervals b_int_. Hence, the duration of the spawning period is given by n_b_ multiplied by b_int_. If less energy than b_max_ is available, batch size is reduced accordingly. We define gonad mass as equal to the mass of the eggs produced in a batch. This increases as energy is allocated to a batch over b_int_, then is reset to zero when that batch is spawned. The egg production of all females is divided equally among n_cohort_ new individuals (eggs) each year. We assume that male and female investment in reproduction is equal.

#### Energy reserves

Larval mackerel prioritse growth [54] over energy storage. Juveniles and adults store energy as lipid [24,80] in preparation for maturation, spawning and, for adults, the winter fast. Individuals can store energy up to their maximum possible energy reserve E_max_ (see TRACE section 2.3). The energy cost of synthesising lipid L_s_ is accounted for when assimilated energy is converted to energy stores. The mass of stored lipid and, for adults, the gonads are added to structural mass to get total mass M.

#### Egg development

While embryo duration in S. scombrus decreases with temperature, background mortality rate M_back_ increases. Hence, the cumulative proportion of eggs that die from M_back_ varies little except at extreme temperatures [49] (see TRACE section 2.4) not encountered in the model (see TRACE section 2.4). We therefore assume for simplicity that the egg development period is five days and M_back_ is constant at rate M_e_ (see Mortality).

#### Ontogenetic transformation

Eggs transform into yolk-sac larvae at length L_hatch_ once reaching the end of their development period. Thereafter individuals transform into larvae (cease to be nourished by the yolk sac) when they reach 0.61 cm [72]; into juveniles when they reach 3 cm (at which point *S.scombrus* have been observed to exhibit active taxis and schooling behaviour; Sette [65]); and can sexually mature as adults after reaching 26.2 cm (L_mat_). For simplicity juveniles with a sufficient length all reach maturity on the same day each year, February 1st. At this point they join the adult migration towards the spawning area.

#### Mortality

The ways in which the abundance n of of an individual can decrease are outlined below.

Starvation: If a SIs‘ total mass reduces to its structural mass it is removed from the model.

Predation: If a SI is selected as prey for a larger SI, its abundance is reduced by M_pred_. M_pred_ is given as ingestion rate IR of the predator (g time-step^−1^) / prey body mass (g), after adjusting the predator's IR by IR_cannibalism_. Hence, M_pred_ depends on the number of predators and SST.

Background mortality: Eggs and larvae are susceptible to background mortality at rate M_e_. Juvenile susceptibility to M_back_ at length L is given by:

(15) $$M_{\text{back}}=M_{a}\frac{L_{\text{mat}}}{L}$$

where M_a_ is a constant equal to adult mortality susceptibility (time-step^−1^), L_mat_ is the threshold length above which juveniles can sexually mature and L is length (cm) [9]. Because background mortality rates decrease with life stage or body length, cumulative mortality depends on growth.

Fishing mortality: Annual rates of fishing mortality rates F (time-step^−1^) are taken from the stock assessment (stockassessment.org). These rates are applied each day, such that the proportion being applied in each month is proportional to the historical proportion of annual catch in that month (Fig. 3).

M_back_ and F are converted to a proportion of a SI‘s abundance dying in a time-step as $1-e^{-(M_{\text{back}}+F)\;}$. SIs with abundance < 1 are removed from the model.

#### Recruitment

Recruitment is defined as the number of young-of-the-year that survive to December 31st each year. This depends on the total number of eggs spawned, and the fraction of those eggs that survive. The number of eggs spawned is determined by the amount of energy that the spawning stock is able to accumulate prior to spawning, which reflects the feeding opportunities available over the previous summer. The fraction of eggs that survive to the end of their first year depends largely on the previling environmental conditions on the spawning grounds. Mortality rate is inversely related to body size meaning that, if conditions favour quick growth (e.g. high prey availability and temperature), then cumulative mortality in that cohort is reduced and more SIs recruit. Users can choose to substitute this 'emergent“ recruitment scheme for a more traditional Ricker-type stock-recruitment relationship that has been fitted to data from the stock assessment. Doing so removes the egg and larval stages from the IBM; instead, recruits enter the model on December 31st. See TRACE section 2 for details of the Ricker model.

## Model calibration and validation

In the following we outline how SEASIM-NEAM was calibrated and evaluated following the principles of “pattern oriented modelling” (see e.g. [12,^25^,^27^,^44^,61]), i.e. by assessing its ability to match spatial and temporal patterns at the individual and population level.

### Model calibration

Values for three of SEASIM-NEAM's parameters cannot be justifiably extracted from the literature. These parameters are the half saturation constant (h) and strength of the competition effect (c) in the functional feeding response, and the background early mortality rate for eggs and larvae (excluding explicit cannibaism, M_e_). Although alternative algorithms are available (e.g. [17,^70^,77]), we estimate these parameters by fitting SEASIM-NEAM to available data using rejection Approximate Bayesian Computation (ABC). Generally following van der Vaart et al. [76], the ABC comprises: 1) selecting data to which the model should be fitted; 2) generating prior distributions for the three parameters; 3) running a number of simulations while randomly sampling values of the parameters from their prior distributions; 4) accepting the parameters that resulted in the best fits to the data; and 5) examining the accepted parameters (i.e. the posterior distributions).

**Fig. 6:**
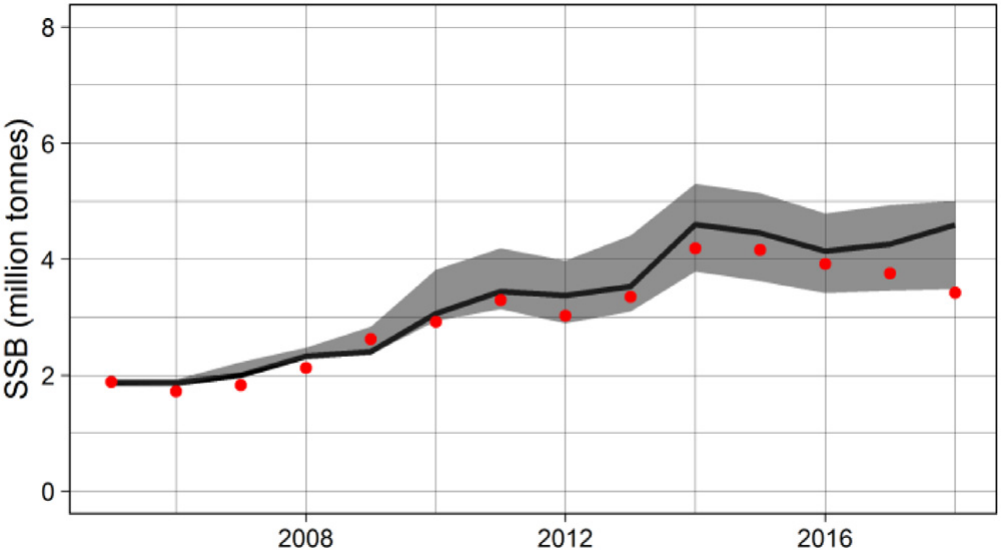
Predicted vs observed (stock assessment, red dots) SSB. The black line denotes the single best-fitting simulation from the ABC, and the grey shaded area delimits the 95% credible intervals from the accepted 1% of 2000 simulations (i.e. the posterior parameter uncertainty).

**Fig. 7:**
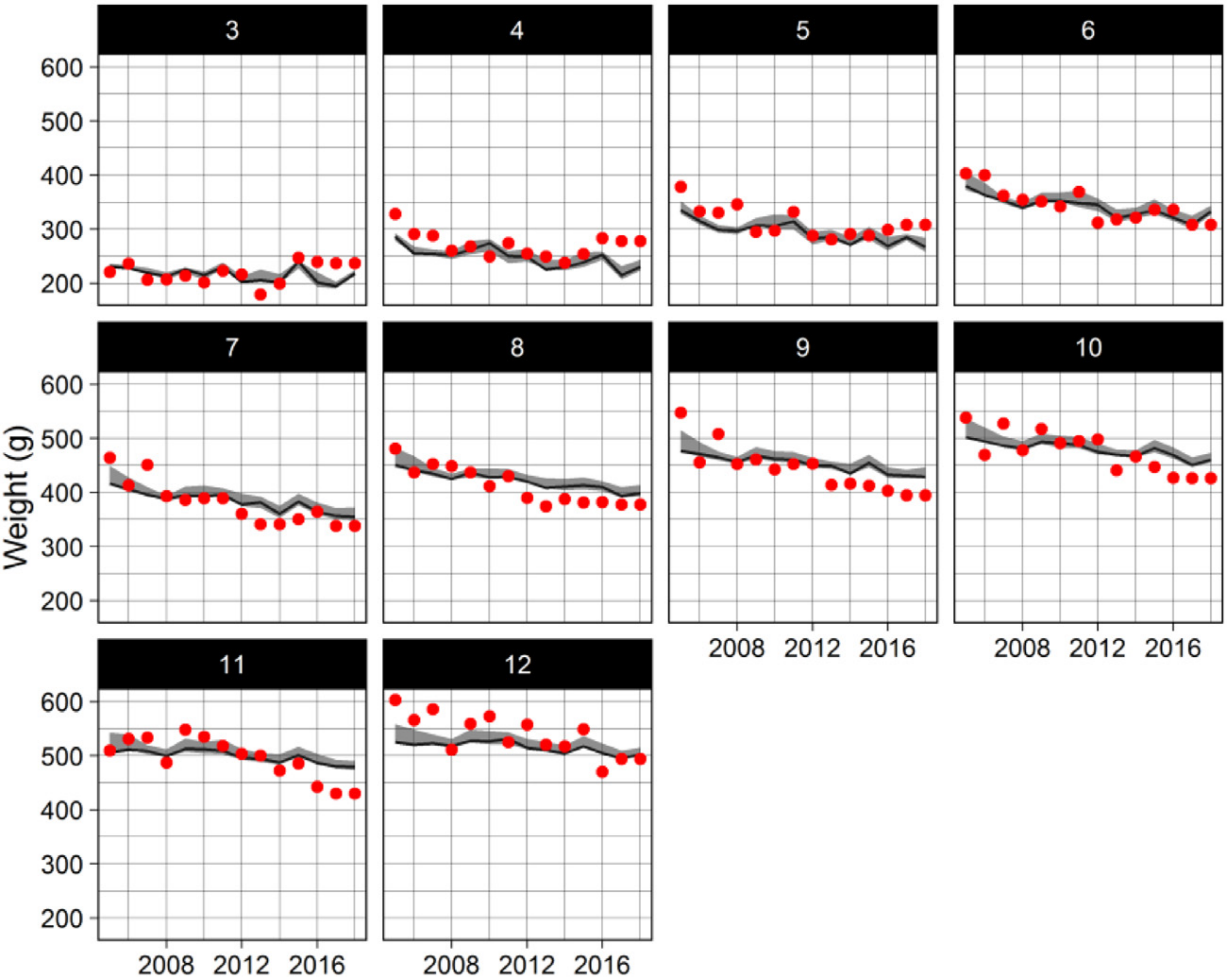
Predicted (SEASIM-NEAM, black line) and observed (red dots) weight at ages three to twelve at spawning time (extracted May 1st). The grey shaded region represents the 95% credible intervals of the posterior distributions as estimated by ABC.

We suggest that SEASIM-NEAM should be fitted to estimates of SSB from the latest NEAM stock assessment, and data on weight-at-age (spawning time). For both variables data are freely available at www.stockassessment.org, where they are updated annually. Note that, if new SSB data from the stock assessment is used for calibration, then fishing mortality from the same stock assessment must be used as input to the IBM. If SEASIM-NEAM can simultaneously match data SSB and weight-at-age, then competition for food at a given SSB can be considered realistic, as reflected by the individual body weights of the fish. In addition, the model's ability to match data at both the individual (body weights) and population level (biomass) gives credibility to its underlying structure. For M_e_, c and h we use uninformative uniform prior distributions spanning intentionally wide bounds, but users can define their own priors based on e.g. expert knowledge. In previous applications we have run 1000 - 4500 simulations, but again users can decide on how many simulations are sufficient for their purposes. We use the sum of the squared deviations of the model outputs from the data as a cost function, but normalise the deviations to account for differences in units between the datasets. Full details of the most recent ABC can be found in TRACE section 3. We also provide an annotated R script in the supplementary material which can be used to conduct future calibrations. Figs. 6 and 7 show the fits of SEASIM-NEAM to the data on SSB and weight-at-age, respectively.

### Model validation using data on the summer distribution

**Fig. 8:**
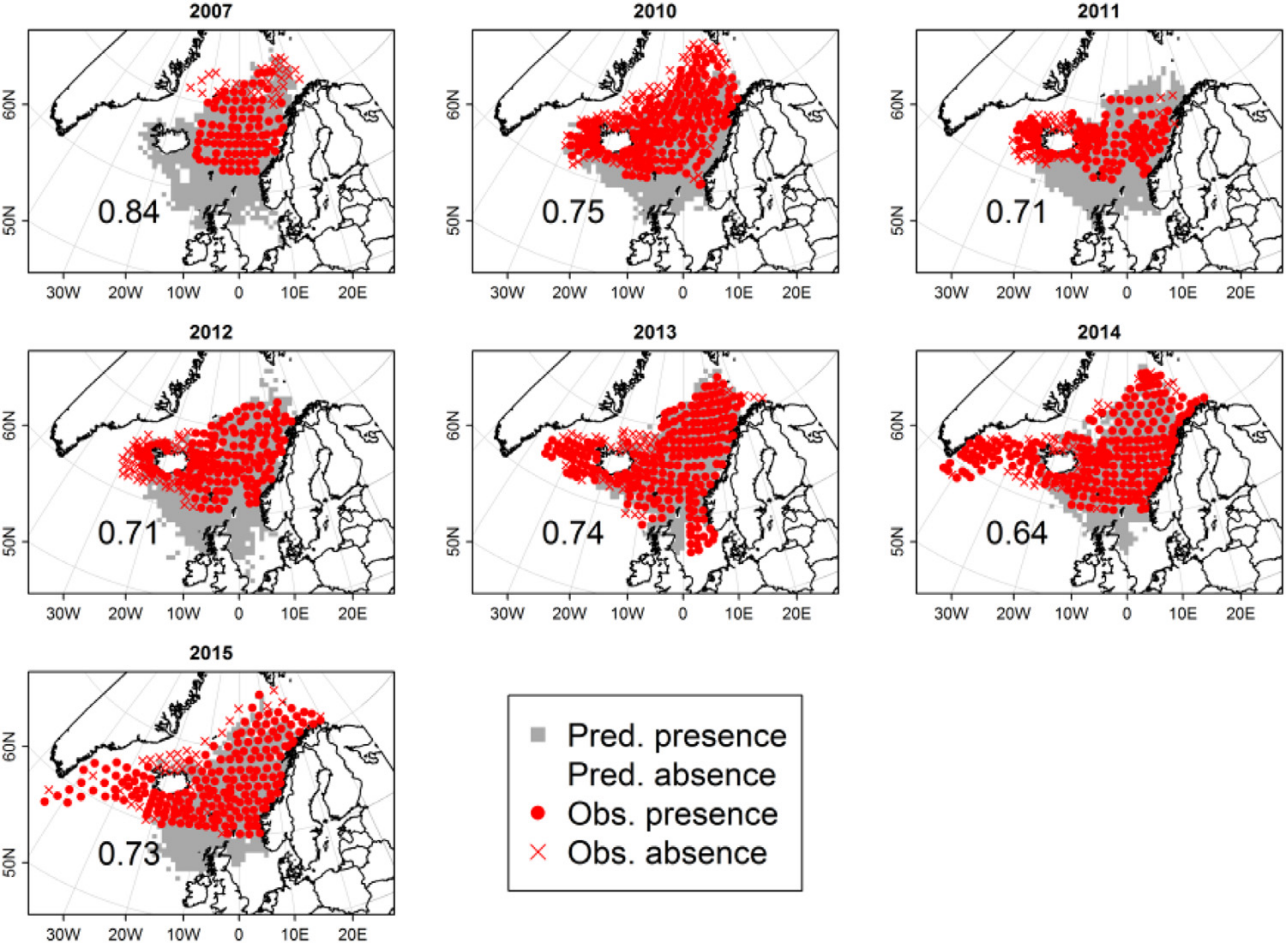
Predicted and observed presence/ absence of NEAM over July/ August. Predictions were obtained after optimising a threshold mackerel density above which a patch is classed as a presence, and below which it is classed as an absence (see text). The numbers on each panel represent the total proportion of data points for which SEASIM-NEAM correctly predicted whether or not NEAM were present.

To assess SEASIM-NEAM's ability to match data that was not used in the ABC, we compare its predictions of presence/ absence over July/ August to observations from the Internation Ecosystem Survey in the Noridc Seas (IESSNS, Fig. 8). See Nøttestad et al. [51] for details of these data, which we approximated from Olafsdottir et al. (2018) using Java's PlotDigitizer (http://plotdigitizer.sourceforge.net/). To assess model fits we used two standard statistics for binary data, sensitivity and specificity, i.e. the proportion of presences and absences correctly classified, respectively. As is standard, we first optimised a threshold mackerel density (patch^−1^) above which that patch is classed as a presence, and below which it is classed as an absence (see TRACE section 7 for full details) [11,47]. After optimising this threshold, sensitivty and specificity values of 0.73 and 0.68 were obtained, respectively. Note that we pooled the predictions and data across all (surveyed) years over 2007 to 2015. This gives extra weight to years in which sampling effort was higher, which we consider appropriate. See Fig. 8 for a comparison of predicted and observed presence/ absence over the summer months.

## Declaration of Competing Interest

The authors declare that they have no known competing financial interests or personal relationships that could have appeared to influence the work reported in this paper.

## Appendix Supplementary materials

Image, application 1

Image, application 2
