# Supplementary material for: SEASIM-NEAM: A Spatially-Explicit Agent-based SIMulator of North East Atlantic Mackerel population dynamics
Source: MethodsX. 2020 Aug 29;7:101044. doi: 10.1016/j.mex.2020.101044 (PMC7490848; doi:10.1016/j.mex.2020.101044)
Supplement: Supplementary file 1 [file mmc1.docx]

ABC_analysis

Rob Boyd

26/04/2020

Load packags

library(car)

## Loading required package: carData

library(Metrics)
library(ggplot2)
library(reshape2)

Set up a couple of things

subset <- "true" # if true, allows users to set aside data for validation

feeding_strategy <- "4.0.1_" ## tell R which version of SEASIM-NEAM you want the outputs from

Load the outputs from the simulations, and the parameters that gave rise to those outputs

stats <- t(read.table(paste0("F:/SEASIM-MAC_2020/outputs/ABC/",feeding_strategy,"1.txt")))

for (i in 2:20) {
 if (file.exists(paste0("F:/SEASIM-MAC_2020/outputs/ABC/",feeding_strategy,i,".txt"))) {
 stats<-rbind(stats, t(read.table(paste0("F:/SEASIM-MAC_2020/outputs/ABC/",feeding_strategy,i,".txt"))))
}
}

length(stats[,1]) # number of simulations

## [1] 2000

## READ IN THE PARAMETER VALUES

params<-read.table(paste0("F:/SEASIM-MAC_2020/outputs/ABC/",feeding_strategy,"1param.txt"))

for (i in 2:20) {
 if (file.exists(paste0("F:/SEASIM-MAC_2020/outputs/ABC/",feeding_strategy,i,"param.txt"))) {
 params<-rbind(params, read.table(paste0("F:/SEASIM-MAC_2020/outputs/ABC/",feeding_strategy,i,"param.txt")))
}
}

summary(as.logical(duplicated(params))) ## check for duplicates

## Mode FALSE
## logical 2000

## identify which outputs correspond to what metric (here weight at age and SSB)

stat_types = c("W3","W4","W5","W6","W7","W8",
"W9","W10","W11","W12","SSB")

names<-c()

for(i in stat_types) {
 for (j in 2005:2018) {
 names<-c(names, paste0(i,j))
}
}

colnames(stats)<-names

Now we can see what the model outputs look like: a dataframe in which each row represents one simulation, and each column one metric/ year combination

head(stats, 3)

## W32005 W32006 W32007 W32008 W32009 W32010 W32011 W32012
## X1 237.4935 232.4926 231.8463 221.3329 229.2831 232.6968 231.3640 222.9951
## X2 230.5524 231.5510 224.1260 216.7445 227.5340 215.4455 236.1766 210.3418
## X3 234.8382 230.0075 223.6622 221.7177 227.6611 222.2510 236.7816 218.5771
## W32013 W32014 W32015 W32016 W32017 W32018 W42005 W42006
## X1 219.4437 228.1296 236.4057 231.5466 217.9559 234.7521 305.1166 289.1703
## X2 223.6956 211.0537 230.5774 209.2184 204.7653 214.6451 286.2082 262.3090
## X3 227.1561 221.6182 234.1416 226.8943 214.3371 232.2085 291.9890 269.9202
## W42007 W42008 W42009 W42010 W42011 W42012 W42013 W42014
## X1 279.7584 282.1303 302.7135 308.1211 298.6052 290.7422 282.0360 283.8711
## X2 258.6858 255.3737 268.3165 279.3347 252.1914 259.1819 238.4724 242.5101
## X3 262.1479 253.9062 284.8001 292.3211 278.8744 276.1141 256.9435 270.2486
## W42015 W42016 W42017 W42018 W52005 W52006 W52007 W52008
## X1 308.3748 299.3754 277.4200 295.5470 361.3885 358.2253 343.1525 335.5750
## X2 249.9964 248.8627 220.7792 238.7962 344.1651 317.4225 303.5965 301.3357
## X3 291.1253 281.7747 256.7667 274.7049 346.7098 320.9252 308.0632 301.2575
## W52009 W52010 W52011 W52012 W52013 W52014 W52015 W52016
## X1 362.4809 376.2573 378.5903 355.6673 338.5882 344.9191 374.0862 361.8908
## X2 315.3435 320.2047 322.0267 286.0370 296.7572 283.9010 300.4096 277.2095
## X3 317.6846 344.0421 348.0174 317.5013 313.7349 309.6895 351.5461 330.3679
## W52017 W52018 W62005 W62006 W62007 W62008 W62009 W62010
## X1 336.0562 352.4977 428.0936 417.5517 401.8747 388.1700 419.3284 434.6046
## X2 283.0598 270.4667 391.0750 373.7060 353.1535 346.6547 359.1282 361.7556
## X3 310.6591 314.3362 389.4498 374.2586 358.9415 350.7865 369.4402 379.1043
## W62011 W62012 W62013 W62014 W62015 W62016 W62017 W62018
## X1 430.8273 425.4561 400.8476 389.5796 436.0929 419.5727 389.5196 405.0577
## X2 358.9537 353.7231 328.0374 336.9586 345.3277 335.5306 316.4645 332.0978
## X3 394.6191 381.1475 357.8989 359.9864 386.0078 384.5230 356.0702 371.6980
## W72005 W72006 W72007 W72008 W72009 W72010 W72011 W72012
## X1 477.4010 464.2567 446.0476 430.2693 468.2369 477.4536 480.3702 466.1398
## X2 433.8418 414.4990 403.5693 388.1848 406.1597 403.9719 402.0674 386.0718
## X3 436.3475 412.0503 403.0366 393.1077 413.9152 423.2701 425.6786 418.6475
## W72013 W72014 W72015 W72016 W72017 W72018 W82005 W82006
## X1 454.4670 439.1102 474.0302 473.8852 431.5758 455.4329 524.7829 503.0662
## X2 385.1873 367.2168 395.5836 377.6400 367.6269 362.8849 465.6098 456.4726
## X3 408.5947 398.8682 426.5452 416.5656 401.7947 410.6521 480.4726 457.5992
## W82007 W82008 W82009 W82010 W82011 W82012 W82013 W82014
## X1 489.3194 473.3581 511.6670 522.9861 519.3057 502.4366 495.8483 487.6472
## X2 438.4214 430.0405 438.0761 442.2677 435.6400 428.8913 418.1813 419.0559
## X3 440.5868 432.9368 457.5516 453.2465 460.5731 441.2313 442.7105 443.6880
## W82015 W82016 W82017 W82018 W92005 W92006 W92007 W92008
## X1 521.6318 508.5152 470.0905 484.1961 562.4773 543.1797 521.9971 508.6179
## X2 419.5418 417.2923 404.5350 408.5772 496.0586 482.6583 470.8835 461.4683
## X3 460.0642 452.3049 430.9063 448.1536 505.1040 489.6356 471.7989 468.8296
## W92009 W92010 W92011 W92012 W92013 W92014 W92015 W92016
## X1 544.4866 561.1611 560.6998 536.5496 520.5943 518.9497 564.4368 549.3898
## X2 475.8293 469.0458 473.1167 455.7349 449.9642 445.2596 461.0940 436.1849
## X3 491.5211 487.3886 483.0861 474.3817 465.9766 472.2510 499.0730 479.0869
## W92017 W92018 W102005 W102006 W102007 W102008 W102009 W102010
## X1 498.2302 517.6091 590.3457 568.5372 548.8135 528.9758 577.3952 583.1787
## X2 436.9624 441.6126 516.1876 509.7613 494.2219 485.3514 506.2249 500.3080
## X3 463.0084 475.6588 529.9544 511.8520 493.9005 486.3685 512.4829 520.4523
## W102011 W102012 W102013 W102014 W102015 W102016 W102017 W102018
## X1 588.6378 565.3340 547.3180 534.3326 583.2414 575.9429 529.3431 546.3924
## X2 493.2770 480.9428 473.4336 471.3017 485.3694 473.3417 458.4201 466.1801
## X3 513.4758 496.1561 498.2911 494.5936 522.5934 514.1806 487.0071 500.1016
## W112005 W112006 W112007 W112008 W112009 W112010 W112011 W112012
## X1 591.9315 590.9544 569.4219 546.4324 601.3070 615.0502 618.6410 591.9254
## X2 520.0663 524.8593 516.0620 507.2048 522.9094 520.4016 520.8713 501.8095
## X3 540.5815 531.7628 515.7264 508.6010 530.1822 533.8129 546.0240 520.2181
## W112013 W112014 W112015 W112016 W112017 W112018 W122005 W122006
## X1 573.5665 558.6183 610.5125 602.8923 546.7545 569.2179 613.8054 599.0629
## X2 499.7533 493.4690 504.9980 492.4536 487.1884 486.0356 538.0533 535.5841
## X3 513.8650 513.2433 540.3366 528.8738 509.1882 522.6573 550.2849 541.0547
## W122007 W122008 W122009 W122010 W122011 W122012 W122013 W122014
## X1 598.5352 565.0131 601.6623 634.0729 645.2841 621.3512 599.6381 584.6982
## X2 533.1495 525.4672 543.4229 537.6848 535.5572 527.6767 516.0853 513.8740
## X3 535.6483 530.4886 555.9867 557.0222 556.7647 546.2093 536.2228 532.3952
## W122015 W122016 W122017 W122018 SSB2005 SSB2006 SSB2007 SSB2008
## X1 627.1971 625.5495 582.9434 582.2328 2.012889 2.082657 2.267910 1.980504
## X2 532.0650 513.6866 502.0920 510.9962 1.872477 1.895213 2.101551 2.556619
## X3 560.3784 551.7998 526.2701 543.8089 1.923888 1.892086 2.043068 1.773307
## SSB2009 SSB2010 SSB2011 SSB2012 SSB2013 SSB2014 SSB2015
## X1 1.986142 1.993339 1.826674 1.578990 1.367445 1.270963 1.1621733
## X2 2.893680 3.511409 3.986850 3.883107 4.334106 5.613724 5.5502360
## X3 1.649936 1.545328 1.379042 1.113816 1.020710 1.010052 0.8997529
## SSB2016 SSB2017 SSB2018
## X1 0.9957602 0.8236354 0.7359981
## X2 5.3785393 5.2909211 5.3573563
## X3 0.7731000 0.6464173 0.5716571

Now load the data

dat<- read.csv("F:/SEASIM-MAC_2020/calibration_data.csv")

dat <- dat[dat$Year > 2004 & dat$Year <= 2018,] ## subset the data

ssb<- dat$SSB

ssb <- (ssb *0.8) / 1e+6 ## scale to reflect the fact we only consider the western spawning component (~80% of stock)

all.data <- c(dat$w3, dat$w4,dat$w5,dat$w6,dat$w7,
 dat$w8, dat$w9, dat$w10,dat$w11, dat$w12,ssb)

all.data[1:(length(all.data)-14)] <- all.data[1:(length(all.data)-14)] * 1000 ## convert from kg to g

Now that we have the model outputs and the data, we can run the ABC analysis. First, users can decide to set aside some data for “validation” (an out of bag sample). Here, we set the out of bag sample to zero, i.e. we use all data for model fitting

x<-0 # x is the number of data points to set aside and not use for model training

#############

calib_period <- function (x,output) {

 sub_stats <- stats[,(1:(14-x))]
 sub_data <- all.data[1:(14-x)]

 for (i in 1:10) {
 sub_stats <- cbind(sub_stats, stats[,((i*14)+1):(((i+1)*14)-x)])
 sub_data <- c(sub_data, all.data[((i*14)+1):(((i+1)*14)-x)])

 }

if (output == "data") {
return(sub_data)
} else
{
return(sub_stats)
}
}

sub_stats <- calib_period(x=x, output="stats")
sub_data <- calib_period(x=x, output="data")

Now to calculate the sum of the squared differences between the model outputs and the data. First, calculate the mean absolute deviation from the median for each output type (weight-at-age or SSB). This is used to normalize the distance calculations so that they are not affected by the different units

for (i in 1:length(sub_stats[1,])) {
 assign(paste0("mad_",i), mad(sub_stats[,i]))
}

mads<-c()

for (i in 1:length(sub_stats[1,])) {
 mads<-c(mads, get(paste0("mad_",i)))
}

wage_mad<-mean(mads[1:(length(sub_data)- (14 - x))])
wage_mad

## [1] 26.89929

ssb_mad<-mean(mads[((length(sub_data)- (14 - x))+1) : length(sub_data)])
ssb_mad

## [1] 2.728256

mads<-c(rep(wage_mad, (length(sub_data)- (14 - x))), rep(ssb_mad, (14-x)))

Now calculate the weighted deviations of the model outputs from the data

for(i in 1:length(sub_stats[,1])) {
 assign(paste0("d_",i),0)
}

## set up weights for the calibration

w<-c(rep(1/15, (length(sub_data)- (14 - x))),rep(1, (14-x)))

for(i in 1:length(sub_stats[,1])) {
 for (j in 1:length(sub_stats[1,])) {
 assign(paste0("d_",i), get(paste0("d_",i)) + (w[j] *(((sub_stats[i,j] - sub_data[j])/mads[j]) ^2)))

 }
}

for(i in 1:length(sub_stats[,1])) {
 assign(paste0("d_",i), sqrt( get(paste0("d_",i))))
}

dists<-c()

for (i in 1:length(sub_stats[,1])) {
 dists<-c(dists, get(paste0("d_",i)))
}

min<-which.min(dists) ## best-fitting parameter set

sim<-1:length(dists)
sum_cal <- cbind(params, dists,sim)

accepted_sims <-sum_cal[order(sum_cal$dists),]
accepted_sims<-accepted_sims[1:(length(params[,1]) *0.01),]

Now we can see the accepted simulations

accepted_sims

## h Me c dists sim
## 12112 1.2615874 0.2867446 9.705957e-12 3.167408 1821
## 2611 1.0566431 0.2927914 9.348771e-12 3.168227 1661
## 403 0.9550973 0.2957587 9.047381e-12 3.194146 640
## 1808 1.0388907 0.2942462 8.671740e-12 3.197604 1880
## 230 1.1854496 0.2905954 8.617519e-12 3.205527 1630
## 1807 1.2894711 0.2862267 9.241326e-12 3.212722 1580
## 1734 1.2307489 0.2911038 9.520946e-12 3.232551 973
## 69 1.1931342 0.2895996 7.312097e-12 3.238239 69
## 83 1.2941368 0.2868770 9.948068e-12 3.240790 83
## 11112 0.8931913 0.2942522 9.451712e-12 3.243664 1811
## 583 1.2509404 0.2923883 8.782625e-12 3.246655 658
## 1258 1.1732991 0.2908436 8.111579e-12 3.253327 1825
## 27 1.2520123 0.2869459 9.494404e-12 3.253453 27
## 1571 1.1530479 0.2880304 9.350206e-12 3.268880 357
## 437 1.1695283 0.2941470 7.629748e-12 3.269595 1443
## 1429 1.2898662 0.2867602 8.971019e-12 3.271235 1714
## 976 0.9462418 0.2932611 9.943183e-12 3.277745 1297
## 1211 0.9833218 0.3005281 9.241502e-12 3.279614 321
## 368 1.2644706 0.2835076 9.442906e-12 3.285472 1736
## 1742 1.2735609 0.2897282 9.191126e-12 3.289748 574

Next compare the prior and posterior distributions of the three parameters. Using a Levene’s test, we can check to see if the posteriors are narrower than the priors

cred_ints<-c()

for(i in 1:3) {
 cred_ints<-c(cred_ints, quantile(accepted_sims[,i],probs=c(0.025,0.975)))
}

cred_ints

## 2.5% 97.5% 2.5% 97.5% 2.5%
## 9.183903e-01 1.292108e+00 2.847992e-01 2.982626e-01 7.462981e-12
## 97.5%
## 9.945748e-12

for (i in 1:3) {
merge<-c(params[,i],accepted_sims[,i])
groups<-c(rep("prior",length(params[,i])),rep("post",length(accepted_sims[,i])))
assign(paste0("levene",i),leveneTest(merge~groups))
}

## Warning in leveneTest.default(y = y, group = group, ...): group coerced to
## factor.

## Warning in leveneTest.default(y = y, group = group, ...): group coerced to
## factor.

## Warning in leveneTest.default(y = y, group = group, ...): group coerced to
## factor.

sigs<-levene1[1,3]
sigs

## [1] 0.005002694

for ( i in 2:3) {
 sigs<-c(sigs, get(paste0("levene",i))[1,3])
}

sigs

## [1] 5.002694e-03 9.850004e-11 3.822988e-09

labels<-c("h",expression("M"["e"]), "c")

par(mfrow=c(1,3),oma=c(2,2,3,2))
for(i in 1:3) {
 hist(params[,i], col="grey",border="grey",main=labels[i],breaks=10,xlab="")
 hist(accepted_sims[,i], col="black",border="black",add=T,breaks=5)
 abline(v=accepted_sims[1,i],col="red")

 if (sigs[i] < 0.05) {
 text(((min(params[i])+ max(params[,i]))/2), 80, "*",cex=4)
 }

 if (i ==2) {
}
}


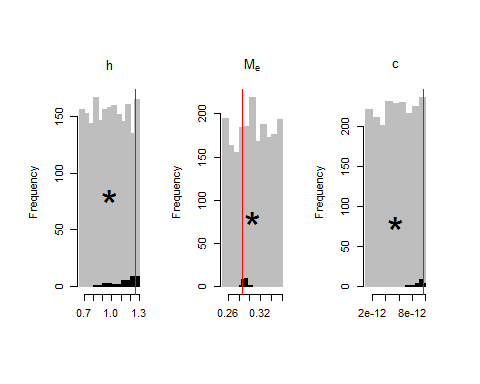


Next we can plot out the best-fitting model outputs vs the data, and quantify the goodness of fit

accepted_stats<- stats[accepted_sims$sim,]

uppers<-c()
lowers<-c()

for (i in 1:length(accepted_stats[1,])) {
 uppers=c(uppers, quantile(accepted_stats[,i], probs=0.975))
 lowers = c(lowers, quantile(accepted_stats[,i], probs=0.025))
}

dat<- data.frame(as.numeric(accepted_stats[1,141:length(accepted_stats[1,])]))

up <- as.numeric(uppers[141:154])
low <-as.numeric(lowers[141:154])

years<-2005:2018

#png("ssb_4.0.2.png",units="in",width=5,height=3,res=500)
ggplot(data=dat, aes(x=years, y= as.numeric(accepted_stats[1,141:length(accepted_stats[1,])]))) +
 geom_line(colour="black",size=1) +
 geom_ribbon (aes(alpha=0.5,ymax=up,ymin=low))+
 ylab("SSB (million tonnes)") +
 xlab("") +
 ylim(c(0,8)) +
 theme_linedraw() +
 geom_point (data=NULL, aes(x=years,y=ssb), colour="red") +
 scale_alpha_continuous(guide=F)


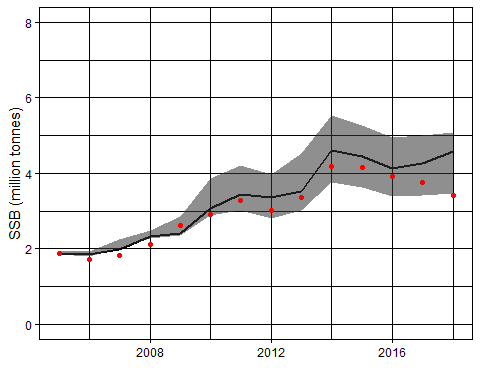


#dev.off()

cor.test(unlist(dat), all.data[141:length(all.data)])

##
## Pearson's product-moment correlation
##
## data: unlist(dat) and all.data[141:length(all.data)]
## t = 12.226, df = 12, p-value = 3.929e-08
## alternative hypothesis: true correlation is not equal to 0
## 95 percent confidence interval:
## 0.8815681 0.9882286
## sample estimates:
## cor
## 0.9621225

bias(predicted=as.numeric(accepted_stats[1,141:length(all.data)]), actual=all.data[141:length(all.data)])

## [1] -0.2637624

wage = accepted_stats[,1:140]

best= wage[1,]

## now extract the min and max

mins<-c()
maxs<-c()

for (i in 1:length(wage[1,])) {
 mins<-c(mins, quantile(wage[,i], probs=0.025))
 maxs<-c(maxs, quantile(wage[,i],probs=0.975))
}

year<-rep(2005:2018, 10)
age <- c(rep(3, 14),rep(4, 14), rep(5, 14), rep(6, 14), rep(7, 14), rep(8, 14), rep(9, 14),
 rep(10, 14), rep(11, 14), rep(12, 14))

x<-cbind(as.numeric(best), mins,maxs, year, age)
x<-data.frame(x)
colnames(x) <- c("best","min","max","year","age")

#png("4.0.2_wage.png",units="in",width=6,height=5,res=500)
p = ggplot (x, aes(x= year, y = best)) +
geom_line(aes(x=year, y=best)) +
geom_ribbon (aes(alpha=0.5,ymax=max,ymin=min)) +
theme_linedraw() +
xlab("") +
ylab("Weight (g)") +
scale_alpha_continuous(guide=F) +
scale_fill_discrete(name ="RCP") +
scale_alpha_continuous(guide=F) +
geom_point (data=NULL,aes(x=year,y=all.data[1:140]), colour="red")

## Scale for 'alpha' is already present. Adding another scale for 'alpha',
## which will replace the existing scale.

p + facet_wrap (~age)


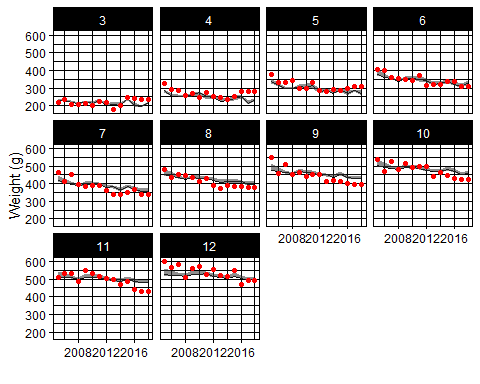


#dev.off()

bias(predicted=x$best, all.data[1:140])

## [1] 3.880153

cor.test(x$best, all.data[1:140])

##
## Pearson's product-moment correlation
##
## data: x$best and all.data[1:140]
## t = 43.168, df = 138, p-value < 2.2e-16
## alternative hypothesis: true correlation is not equal to 0
## 95 percent confidence interval:
## 0.9512897 0.9747696
## sample estimates:
## cor
## 0.964909
