# Supplementary material for: SEASIM-NEAM: A Spatially-Explicit Agent-based SIMulator of North East Atlantic Mackerel population dynamics
Source: MethodsX. 2020 Aug 29;7:101044. doi: 10.1016/j.mex.2020.101044 (PMC7490848; doi:10.1016/j.mex.2020.101044)
Supplement: Supplementary file 2 [file mmc2.docx]

TRACE document

This technical specification is a TRACE document (“TRAnsparent and Comprehensive model Evaludation”) which provides supporting evidence that our model presented in:

- Boyd, R., Roy, S., Sibly, R., Thorpe, R., & Hyder, K. (2018). A general approach to incorporating spatial and temporal variation in individual-based models of fish populations with application to Atlantic mackerel. *Ecological Modelling*, *382*(April), 9–17. <https://doi.org/10.1016/j.ecolmodel.2018.04.015>
- Boyd, R., Sibly, R., Hyder, K., Walker, N., Thorpe, R., Roy, S. (2020). Simulating the summer feeding distribution of Northeast Atlantic mackerel with a mechanistic individual-based model. Progress in Oceanography
- Boyd, R., R. Thorpe, K. Hyder, S. Roy, and N. Walker. 2020. Potential Consequences of Climate and Management Scenarios for the Northeast Atlantic Mackerel Fishery. Frontiers in Marine Science

was thoughtfully designed, correctly implemented, thoroughly tested, well understood, and appropriately used for its intended purpose.

The rationale of this document follows:

Schmolke A, Thorbek P, DeAngelis DL, Grimm V. 2010. Ecological modelling supporting environmental decision making: a strategy for the future. *Trends in Ecology and Evolution* 25: 479-486.

and uses the updated standard terminology and document structure in:

Grimm V, Augusiak J, Focks A, Frank B, Gabsi F, Johnston ASA, Kułakowska K, Liu C, Martin BT, Meli M, Radchuk V, Schmolke A, Thorbek P, Railsback SF. 2014. Towards better modelling and decision support: documenting model development, testing, and analysis using TRACE. *Ecological Modelling*

and

Augusiak J, Van den Brink PJ, Grimm V. 2014. Merging validation and evaluation of ecological models to ‘evaludation’: a review of terminology and a practical approach. *Ecological Modelling*.

Contents

[1 Problem formulation 4](#_Toc48150874)

[2 Model description 4](#_Toc48150875)

[2.1 Purpose 4](#_Toc48150876)

[2.2 Model overview 5](#_Toc48150877)

[2.3 State Variables and Scales 5](#_Toc48150878)

[2.4 Processes, Overview and Scheduling 8](#_Toc48150879)

[2.5 Design Concepts 10](#_Toc48150880)

[2.5.1 Emergence 10](#_Toc48150881)

[2.5.2 Sensing 10](#_Toc48150882)

[2.5.3 Interaction 10](#_Toc48150883)

[2.5.4 Stochasticity 10](#_Toc48150884)

[2.5.5 Observation 10](#_Toc48150885)

[2.5.6 Initialization 11](#_Toc48150886)

[2.5.7 Input data 11](#_Toc48150887)

[2.5.8 Sub-models 13](#_Toc48150888)

[3 Data evaluation 22](#_Toc48150889)

[3.1 Parameter derivations 22](#_Toc48150890)

[3.1.1 Parameters from the literature 22](#_Toc48150891)

[3.1.2 Calibrated parameters 28](#_Toc48150892)

[3.2 Input data 30](#_Toc48150893)

[3.2.1 Fishing mortality 30](#_Toc48150894)

[3.2.2 Environmental inputs 30](#_Toc48150895)

[4 Conceptual model evaluation 33](#_Toc48150896)

[Summary**:** 33](#_Toc48150897)

[4.1 Choice of food availability and temperature as model inputs 33](#_Toc48150898)

[4.2 Stock spatial distribution 33](#_Toc48150899)

[4.3 Omission of temperature-dependent egg mortality and development 33](#_Toc48150900)

[5 Implementation verification 34](#_Toc48150901)

[Summary: 34](#_Toc48150902)

[5.1 How to install, use and update the model 35](#_Toc48150903)

[5.1.1 User options and model interface 35](#_Toc48150904)

[5.2 Summary of changes to the IBM between versions 37](#_Toc48150905)

[5.2.1 Data used for calibration 37](#_Toc48150906)

[5.2.2 Spatial distribution and movement models 37](#_Toc48150907)

[5.2.3 Model inputs 38](#_Toc48150908)

[5.2.4 Model resolution and extent 38](#_Toc48150909)

[5.2.5 Initialisation 38](#_Toc48150910)

[6 Model output verification 40](#_Toc48150911)

[7 Model analysis 41](#_Toc48150912)

[Summary: 41](#_Toc48150913)

[7.1 Local Sensitivity analysis 41](#_Toc48150914)

[7.2 Sensitivity to number of super-individuals 43](#_Toc48150915)

[7.3 Optimising threshold density for presence 43](#_Toc48150916)

[8 Model output corroboration 44](#_Toc48150917)

# Problem formulation

**This TRACE element provides supporting information on**: The decision-making context in which the model will be used; the types of model clients or stakeholders addressed; a precise specification of the question(s) that should be answered with the model, including a specification of necessary model outputs; and a statement of the domain of applicability of the model, including the extent of acceptable extrapolations.

While SEASIM-NEAM is not intended to be used in a tactical capacity, we hope that it can be used strategically to support management of NEAM. It could be considered as a potential source of information to the ICES working group on widely-distributed and migratory stocks (WGWIDE) who conduct the NEAM stock assessment. The stock assessment model, SAM, is undoubtedly best-able to provide estimates of SSB and the rates of fishing mortality. However, SAM cannot address questions regarding the long-term effects of management options, or of spatial management scenarios. We hope that our IBM could provide answers to such questions as: 1) how will the implementation or removal of area closures affect the stock? 2) how will catch limits affect the stock in the long-term against various climate backdrops? and 3) what fraction of the stock is in various jurisdictions at different times of year?

Care must be taken when using our IBM not to use its predictions inappropriately. Currently movements of individuals in the IBM outside of the summer feeding period are to some extent hard-wired. For this reason only spatial management scenarios pertaining to the Northern region of the IBM domain and in summer can be tested. Moreover, the IBM only represents the western spawning component of the NEAM stock which accounts for roughly 80% of its total biomass. For this reason the IBM should not be considered a reliable predictor of overall stock size. Finally, it is possible for the user to use environmental inputs to the IBM provided from an earth system model GFDL-ESM-2M. We have used these inputs to drive the IBM into the future and make forecasts about the effects of long-term management and climate scenarios. However, these inputs are themselves outputs from another model so should not be used when seeking information on historical NEAM population dynamics or spatial distribution (remote-sensing inputs should be used instead).

# Model description

**This TRACE element provides supporting information on**: The model. Provide a detailed written model description. For individual/agent-based and other simulation models, the ODD protocol is recommended as standard format. For complex submodels it should include concise explanations of the underlying rationale. Model users should learn what the model is, how it works, and what guided its design.

Summary**:**

**In this section we provide an ODD (overview, design concepts and details) description of the model and its parameterization for *S. scombrus*** (Grimm et al. 2006, 2010)**.**

## Purpose

SEASIM-NEAM was designed to capture the ways in which *S. Scombrus* population dynamics, structure and distribution respond to spatial and temporal variations in prey availability, temperature and exploitation. It can be used to assess how various environmental and management scenarios may impact the stock.

## Model overview

In broad terms, the model landscape consists of dynamic maps of sea surface temperature (SST), phytoplankton biomass, and horizontal current velocities (zonal and meridional). These variables are derived from satellite remote-sensing (see TRACE section 3). Fish are grouped into super-individuals (hereafter SIs), which comprise a number of individuals with identical variables (Scheffer et al. 1995). Individuals move around the landscape according to their life cycles (e.g. to spawn, feed and overwinter). Each SI has an energy budget which determines how its characteristics (e.g. body size, life stage, energy reserves) change in response to local food availability and SST. Time and age-varying fishing pressure determines the rate of mortality from exploitation. A constant number of new SIs enter the model as eggs at spawning time each year, but the abundance that they represent is determined by the amount of energy the spawning stock has put into egg production. The amount of energy that the spawning stock can accumulate for egg production is an emergent feature of the bioenergetics model and reflects the feeding opportunities available to adults prior to spawning. Abundance reduces as fishing and natural mortalities are applied throughout life. Population measures such as SSB and recruitment are obtained by summarising the characteristics of all the individuals including their abundances.

## State Variables and Scales

The model landscape comprises a two-dimensional grid of patches of sea surface (Fig. 1). The spatial extent spans from 47 to 77°N, and from -45° to 20°E. Each patch represents 60 x 60 km and is characterised by prey density, sea surface temperature (SST), mackerel density, photoperiod (proportion of day length) and horizontal current velocities in x and y dimensions. The mackerel population is represented by a constant number n of SIs (chosen by the user); as n_cohort_ new SIs enter the model as eggs each year, an equal number reach terminal age (> 15 years) and are removed from the model. While the number of SIs remains constant, the abundance that they represent differs: A SI’s abundance is determined by the level of egg production in the year that it entered the model, and all subsequent mortality. Each SI is characterised by e.g. age, gender, life stage (egg, yolk-sac larvae, larvae, juvenile or adult), length, mass (structural, lipid and gonad), abundance and location (see Table 1 for a full list of state variables). The spawning area comprises patches on the continental shelf edge to the west of the British Isles (500m < depth < 50m) on which 10°C < SST < 12°C (Sette 1943). The feeding area is a fully emergent feature of the IBM and is not constrained geographically. The overwintering area is assumed to be ICES divisions 6a (west of Scotland) and 4a (northern North Sea, see TRACE section 2.4). The nursery area includes all patches that are ≤ 200 m deep (Jansen et al. 2014) to the west of the British Isles (< 4°00 west). The temporal extent spans from January 1^st^ 2005 to December 31^st^ 2018, but may be extended to 2050 if the satellite-derived inputs are substituted for forecasts from an earth system model (GFDL-ESM-2M, see TRACE section 3). The model proceeds in discrete five-day time-steps.


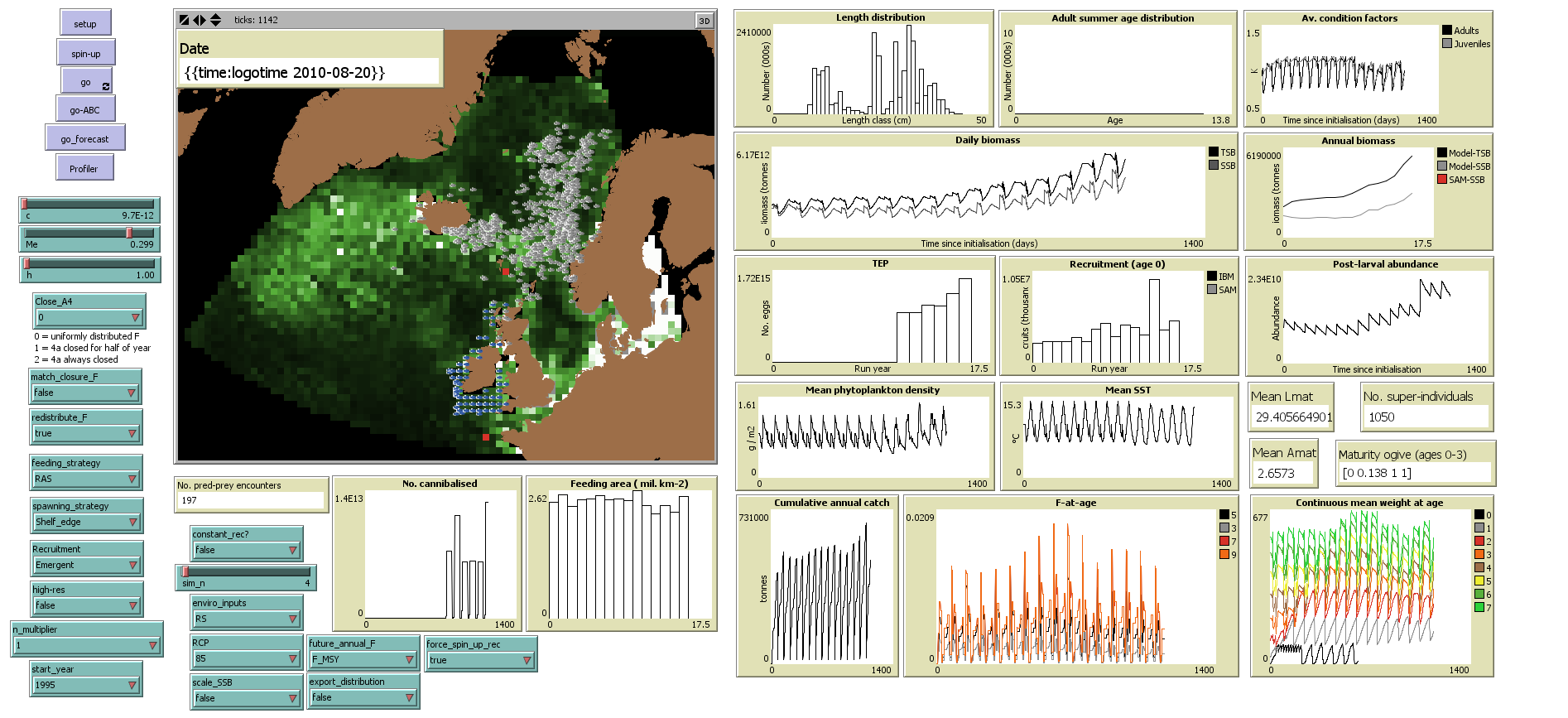


Figure 1. Snapshot of the IBM GUI. There are there are three types of NetLogo widgets shown: Grey buttons (used to initialise and run the model); green “sliders”, used to select input values from a specified range; green “choosers” allowing users to select configuration options from drop down menus; and brown “plots and monitors” displaying e.g. predicted population dynamics. See TRACE section 5 for full details of the interface widgets. Grey SIs in the Nordic sea are adults, and the blue SIs to the west of the British Isles are juveniles. The red cells indicate “destination” patches towards which adults migrate. The southerly red cell is the destination for the spawning migration, and the northerly red cell is the destination for the feeding and return overwintering migrations (see text for details). The colour of the landscape corresponds to phytoplankton density: black indicates low density, through green and then white which indicates high density. The colour bins are arbitrary.

Table 1. Key state variables charcterising SIs and patches. Here we define state variables as variables that cannot be immediately deduced from the state variables of the other entities (Railsback and Grimm 2010). As such this table does not include „rate“ variables (e.g. growth, metabolic rate) which could be calculated from e.g. body size and temperature.

| State variable | Description | Details |
| --- | --- | --- |
| Super-individuals | | |
| Abundance | Number of “actual“ individuals represented by SI |  |
| Age |  | years/ days |
| A_mat_ | Age at which sexual maturity was reached | Years |
| Batches | Cumulative number of egg batches spawned in a season | Used to determine when spawning should cease (when >= 5) |
| Breed | Life stage | Egg, yolk-sac larvae, larvae, juvenile or adult |
| Development | Number of days developed as an egg |  |
| Energy-reserve | Energy stored as lipid | kJ |
| F | Fishing mortality rate | Day^-1^ |
| Feeding | Whether or not the individual is feeding (only half of year for adults) | Boolean |
| Gender |  |  |
| L | Body length | Cm |
| L_mat_ | Length at which sexual maturity was reached | Cm |
| M | Total body mass | g |
| M_gon_ | Gonad mass | g |
| Migrating | Whether or not individual is currently migrating | Boolean |
| M_standard_ | Standard body mass | g |
| M_struct_ | Structural body mass | g |
| Prey-choices | Potential prey (sufficiently small and on same patch as focal individual) | Netlogo ID numbers |
| f_r_ | Realised fecundity | Proportion of potential fecundity |
| V_r_ | Realised swimming velocity | Minimum velocity plus random noise (km hour^-1^) |
| Spawning | Whether or not an individual is spawning | Boolean |
| V_min_ | Minimum swimming velocity | Km hour^-1^ |
| x | x coordinate | Float |
| y | y coordinate | Float |
| Patches | | |
| A4 | In ICES division 4a? | Boolean |
| A5 | In ICES division 5a? | Boolean |
| A6 | In ICES division 6a? | Boolean |
| Depth |  | m |
| Feed-dist | Distance from destination at the entrance to the feeding area | No. Patches |
| Latitude |  | Decimal degrees |
| Longitude |  | Decimal degrees |
| NArea | In nursery area? | Boolean |
| Ocean | In ocean? | Boolean |
| OWArea | In overwintering area? | Boolean |
| p_photo_ | Photoperiod | Proportion of 24 hours |
| X | Phytoplankton biomass | g m^-2^ |
| Rectangle | ICES rectangle |  |
| Ricker-spawn-area | Area designated as spawning grounds for Ricker model | Used as area over which to calculate mean SST for use in Ricker recruitment model |
| SArea | In spawning area? | Boolean |
| Shelf-edge | On the European continental shelf edge? | 550m < depth < 50m |
| Spawn-dist | Distance from destination at end of spawning migration | No. Patches |
| SST | Sea Surface temperature | °C |
| U | Zonal component of current velocity | km hour^-1^ |
| V | Meridional component of current velocity | km hour^-1^ |

## Processes, Overview and Scheduling

Full details of the model processes are given in the *Submodels* sections indicated in parentheses here. The order in which individuals or patches carry out a given process is random. State variables are updated immediately after being calculated by a process. The order in which processes are implemented each time-step are as follows:

1. Phytoplankton and SST data are updated if appropriate (i.e. every tenth day)
2. If it is the first time-step in a year, then annual rates of fishing mortality-at-age are updated
3. If it is the first time-step in a month, then photoperiod, current velocities and the proportion of annual fishing mortality that should be applied in that month are updated
4. Fishing, starvation and background mortalities are applied to SIs (*mortality*)
5. SIs move to a new location (*movement*)
6. SIs update their energy budgets (with the exception of reproduction which comes later; *energy budget*)
7. SIs progress to the next life stage if body size thresholds are met (it must also be February 1st for juveniles to reach sexual maturity)
8. If it is the start of the spawning period (March 1st), adults calculate their potential fecundity and the associated energy cost (*energy budget*)
9. If in the spawning period, adults implement a spawning module. This includes allocating energy to the production of egg batches, spawning those batches at specified intervals, and moving northward as suitably warm regions open up for egg development in the north
10. New SIs enter the model at the egg stage, and calculate their development
11. All SIs age by Δt (days post-hatch)
12. SIs‘ state variables are recorded for analysis outside of the IBM


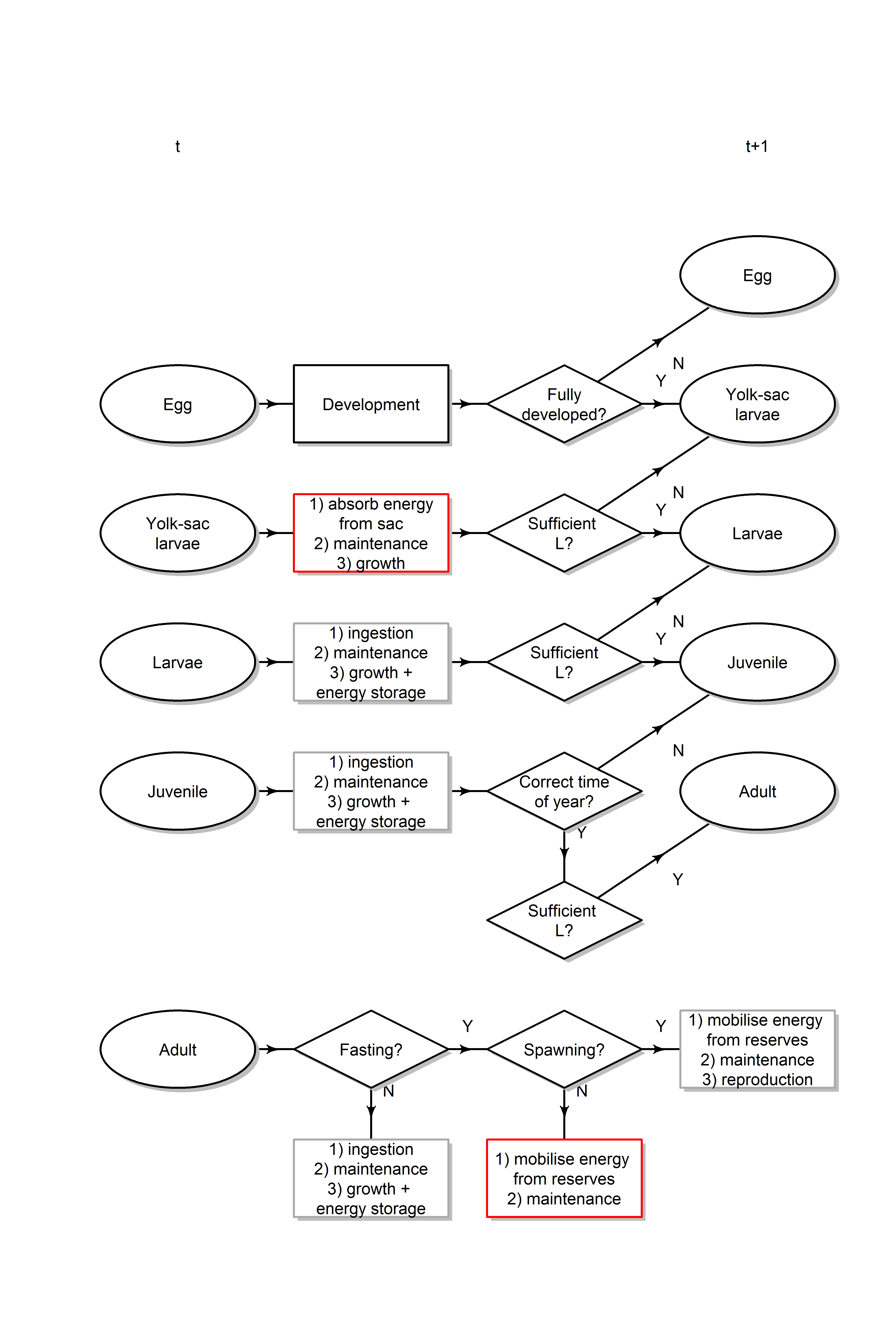


Figure 2. Conceptual model showing the key bioenergetics processes that individuals of different life stages implement between time t and t+1, and the conditions required for progression to the next life stage. Red boxes indicate an effect of SST, and grey boxes an effect of SST and food availability. L is body length.

## Design Concepts

### Emergence

Movement and bioenergetics models describe the ways in which SIs’ characteristics (e.g. body mass, energy reserves and location) respond to their local food availability and SST. By summarising the characteristics of all the SIs, population measures can be obtained. For example, SSB can be obtained by summing the individual body masses of all adults, and spatial distribution by summarising the locations of the individuals.

### Sensing

To direct movement individuals can sense the plankton biomass, SST, depth and area type of all patches, and the global variables that indicate when migrations and spawning should begin. To select prey, SIs can sense one another‘s body length and ID number. In order for density dependence to act an ingestion rates and perceptions of patch profitability (see *Movement*), SIs can sense the density of mackerel on all patches.

### Interaction

Larger individuals can feed on smaller ones, inflicting predation mortality on them and hence depleting fish prey. Individuals on the same patch also compete with each other for baseline prey (proxied by phytoplankton) according to a competition term in equation (2).

### Stochasticity

There are several stochatic elements to the IBM. If not migrating or actively foraging over summer, individuals move randomly to patches within their search radius (see *Movement*) and with suitable environmental conditions. Swimming velocity when feeding is given by a minimum swimming velocity plus some random noise (see *Movement*). In the gradient area search (GAS) foraging model, half of each day is spent moving in a random direction. If multiple potential mackerel prey SIs are available, one is selected randomly to be canniblised. At the end of the feeding migration, SIs stop migrating at a randomly selected distance from their target patch at the entrance to the feeding grounds (see *Movement*). In the spin-up period, recruits enter the model at the end of each year at body length L_1_ (Table 4) minus some random noise.

### Observation

During simulations the state variables of all, or a subset, of the SIs can be extracted and summarised to obtain measures of population dynamics and spatial distribution. Key model outputs are summarised in Table 2.

Table 2. Population metrics obtained by summarising the characteristics of the individuals, and the dates on which they are extracted.

| Metric | Date extracted | Details |
| --- | --- | --- |
| SSB in summer | August 1st | Sum of adult body masses |
| SSB at spawning time | May 1st | Sum of adult body masses |
| Egg production | June 1st | Number of eggs produced by the spawning stock |
| Recruitment | December 31st | Number of young-of-the-year surviving until December 31st |
| Maturity ogives | February 10th | Proportion mature-at-age |
| Adult summer age distribution | August 1st | Relative age distribution (years) of adults |
| Weight of 36cm individuals | Mean each month | Mean body mass of individuals in the 36cm length group |
| Quarter 1 juvenile length distribution | March 16th | Relative body length distribution of juveniles |
| Quarter 4 juvenile length distribution | November 23rd | Relative body length distribution of juveniles |
| Mean weight-at-age in summer | August 1st | Mean body mass in each age group |
| Mean weight-at-age at spawning time | May 1st | Mean body mass in each age group |
| Presence/ absence in summer | Mean over July/ August | Whether or not individuals were present on each patch |
| Density in summer | Mean over July/ August | Density of individuals present on each patch |

### Initialization

The IBM is initialised on January 1st of a chosen year 1980 onwards) using numbers-at-age in from the latest ICES stock assessment. This population is apportioned in to n SIs assuming a gender ratio of 1:1. Body lengths are calculated from age using the standard von Bertalanffy equation (equation 12 here), and energy reserves are set at half maximum. From these all other state variables are calculated when the simulations begin. Adults and juveniles are distributed randomly in the overwintering and nursery areas, respectively. After initialisation the model spins up for ten years with recruitment forced from the ICES stock assessment. Recruits are introduced at the end of each year, with body length set at the maximum length at the end of the first growing season, L_1_ (cm), minus ε 3, where ε is drawn randomly from uniform distribution between 0 and 1.

### Input data

The IBM is forced with estimates of fishing mortality at age, chlorophyll concentration (from which we derive phytoplankton biomass with an empirical conversion factor), SST, zonal and meridional horizontal current velocities and photoperiod. See section TRACE section 3 for details of how these data were processed.

#### Fishing mortality

Historical annual rates of fishing mortality F at age are taken from the latest available version of the NEAM stock assessment (see TRACE section 3 for details). Unless stated otherwise, F is applied uniformly to all individuals in an age group regardless of their location. We incorporate monthly variation in F by setting the fractional of annual F taken in each month as proportional to the mean historical (2001 to 2018) fraction of the annual NEAM catch taken in each month (Table 3).

For future fishing pressure we take mean F-at-age over the historical period 2001 to 2018 (Fig. 3) and adjust it using one of three multipliers. The multipliers are used to set mean F over the most important age groups to the fishery (for NEAM 4-8 years) at one of three rates: F = 0; F_MSY_ (0.23 year^-1^)^,^ i.e. the level of harvesting that is likely to result in maximum sustainable yield in the long-term; and F_lim_ (0.46 year^-1^), i.e. high mortality used as an upper reference point (ICES 2012, 2019a). Monthly variation in F is implemented as in the historical period.


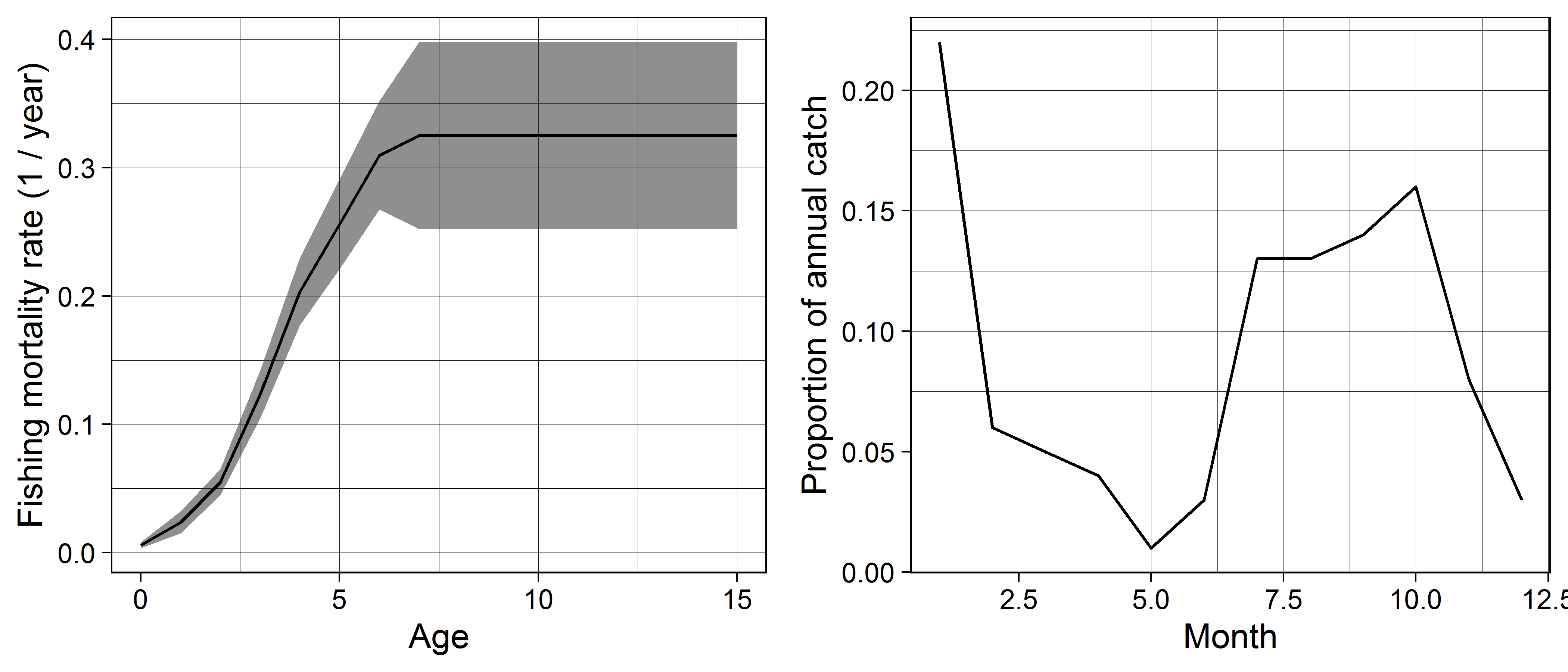


Figure 3. Mean F at age over the historical period 2001 to 2018 (black line) ± one standard deviation (shaded area). F here was taken from the 2019 stock assessment, but can be updated as new stock assessments are conducted each year.

#### Environmental inputs

Environmental inputs to SEASIM-NEAM include maps of chlorophyll concentration, from which we derive phytoplankton biomass (with an empirical conversion factor), SST, bathymetry, photoperiod and horizontal current velocities. Users can select chlorophyll and SST estimates derived from satellite remote-sensing, or from the earth system model GFDL-ESM-2M (Dunne et al. 2013). The satellite-derived inputs comprise ten-day composites and are updated accordingly. The ESM outputs represent monthly averages. The data required processing for use in SEASIM-NEAM (e.g. re-gridding), the details of which can be found in TRACE section 3. When using the ESM inputs users must choose from one of representative concentration pathways (RCPs) 2.6 or 8.5, representing high and low levels of climate change mitigation action, respectively. Forecasts of the environmental inputs are available out to 2050 for each RCP scenario.

Near surface (average over 0 to -30m) horizontal current velocities were taken from the 1/3 ⁰ OSCAR dataset (ESR 2009). Currents influence the movements of adults over summer (equation 4), so we obtained data for the months May through September. Outside of this period current velocities have no effect in SEASIM-NEAM. It would not be appropriate to include the effects of near surface current velocities on individuals outside of the summer period, when mackerel may inhabit deeper waters (e.g. -50 to -220m over winter) (Jansen et al. 2012). Over summer NEAM are found in the upper water layer (average of ~ -20m) (Nøttestad et al. 2016). As data are not available for the selected months prior to 2012, we generated mean climatologies for each month over 2012 to 2018. As such we do not account for inter-annual variability in current velocities.

Data on photoperiod (as a proportion of 24 hours) at all latitudes in the IBM grid was extracted for each month using the daylength() function in the R package geosphere (Hijmans 2019a). Values correspond to the 15^th^ day of each month, and are updated at the start of each month in SEASIM-NEAM.

### Sub-models

Most parameters were derived from the literature as shown in Table 4. Mass is in units of wet weight throughout.

Table 4. Parameters and their values used in the model. All normalizing and rate constants are shown in units of 1/day and are adjusted for the time-step in the IBM.

| Parameter | Symbol | Value | Units | Reference | Notes |
| --- | --- | --- | --- | --- | --- |
| Taxon-specific normalization constant (AMR) | a_AMR_ | 8.86x10^7^ |  | (Dickson et al. 2002) | See TRACE section 2.3 |
| Assimilation efficiency | A_e_ | 0.95 | Proportion of ingested energy | (Lambert 1985) |  |
| Normalizing constant for fecundity | a_f_ | 8.80 |  | (Lockwood et al. 1981) |  |
| Caudal fin aspect ratio | A_r_ | 4.01 |  | FishBase |  |
| Taxon-specific normalization constant (SMR) | a_SMR_ | 0.45x10^8^ |  | (Herrmann and Enders 2000) | See TRACE section 2.3 |
| Swimming speed normalizing constant | a_v_ | 0.15 |  | (Sambilay Jr 1990) |  |
| Exponent for the scaling of AMR with body mass | b_AMR_ | 0.75 |  | (Herrmann and Enders 2000) | See TRACE section 2.3 |
| Scaling exponent for fecundity | b_f_ | 3.02 |  | (Lockwood et al. 1981) |  |
| Exponent for the scaling of SMR with body mass | b_SMR_ | 0.75 |  | (Dickson et al. 2002) | See TRACE section 2.3 |
| Strength of the predator density dependence | c | 2.81x10^-11^ |  | This study | Estimated with ABC (see TRACE section 2.3) |
| Exponent for the scaling of AMR with swimming speed | c_AMR_ | 1 |  | (Dickson et al. 2002) | See TRACE section 2.3 |
| Maximum consumption rate | C_max_ | 0.69 | g g^-1^ day^-1^ | (Hatanaka et al. 1957) | See TRACE section 2.3 |
| Activation energy | E_a_ | 0.5 | eV | (Gillooly et al. 2006) |  |
| Energy content of flesh | E_flesh_ | 7.00 | kJ g^-1^ | (Peters 1983) |  |
| Energy content of lipid | E_lipid_ | 39.3 | kJ g^-1^ | (Schmidt-Nielsen 2013) |  |
| Maximum energy reserves | E_max_ | 0.78 | Proportion of structural mass | (Grégoire et al. 1992) | See TRACE section 2.3 |
| Energy density of phytoplankton | E_phyto_ | 6.02 | kJ g^-1^ | (Annis et al. 2011) |  |
| Energy costs of synthesizing flesh | E_sf_ | 3.60 | kJ g^-1^ | (Sibly and Calow 1986, Sibly et al. 2013) |  |
| Energy costs of synthesizing lipid | E_sl_ | 14.7 | kJ g^-1^ | (Pullar and Webster 1977) | See TRACE section 2.3 |
| Half saturation constant | h | 0.882 | g m^-2^ | This study | Estimated with ABC (see section 2.3) |
| Rate of cannibalism | IR_cannibalism_ | 0.064 | Proportion of ingestion rate | (Pinnegar 2014) | Only relavent where suitable prey are available. |
| Growth constant | k | 8.6x10^-4^ | day^-1^ | FishBase | See TRACE section 2.3 |
| Boltzmann’s constant | K | 8.62 x 10^-5^ | eV K^-1^ |  |  |
| Maximum growth rate | k_1_ | 0.025 | day^-1^ | (Villamor et al. 2004) | See TRACE section 2.3 |
| Maximum length after first growing season | L_1_ | 20 | cm | (Villamor et al. 2004) |  |
| Asymptotic length | L_∞_ | 42.4 | cm | FishBase | See TRACE section 2.3 |
| Length at hatching | L_hatch_ | 0.3 | cm | (Villamor et al. 2004) |  |
| Threshold length for maturity | L_mat_ | 26.2 | cm | FishBase | See TRACE section 2.3 |
| Background adult mortality | M_a_ | 0.00041 | day^-1^ | (ICES 2017) | Constant for all ages, based on tagging studies in 1980’s and used in the stock assessment |
| Background early mortality | M_e_ | 0.281 | day^-1^ | This study | Estimated with ABC (see TRACE section 2.3) |
| Number of egg batches spawned | n_b_ | 5 | season^-1^ | This study | For simplicity, the actual number is around 20 |
| Lower temperature limit | SST_lim_ | 7 | °C | (Ólafsdóttir et al. 2018) |  |
| Age at maximum growth | t_max_ | 55 | days | (Villamor et al. 2004) |  |

#### Movement

The following sub-models describe the ways in which SIs are directed around the landscape. In broad terms, SIs migrate between different areas (e.g. spawning, nursery, feeding), and otherwise move locally within an area. Migrations are date-triggered. Localised movement differs between area, e.g. local movement when spawning in spring differs from local movement when feeding over summer. At most times of year movement is represented in discrete space, i.e. on a patch-by-patch basis. However, we spent considerable time improving the way in which movement is modelled for adults in the summer period (Boyd et al. 2020), which now operates in continuous space (details below). We hope that in time SEASIM-NEAM will be further developed such that all movement is described in a Lagrangian framework.


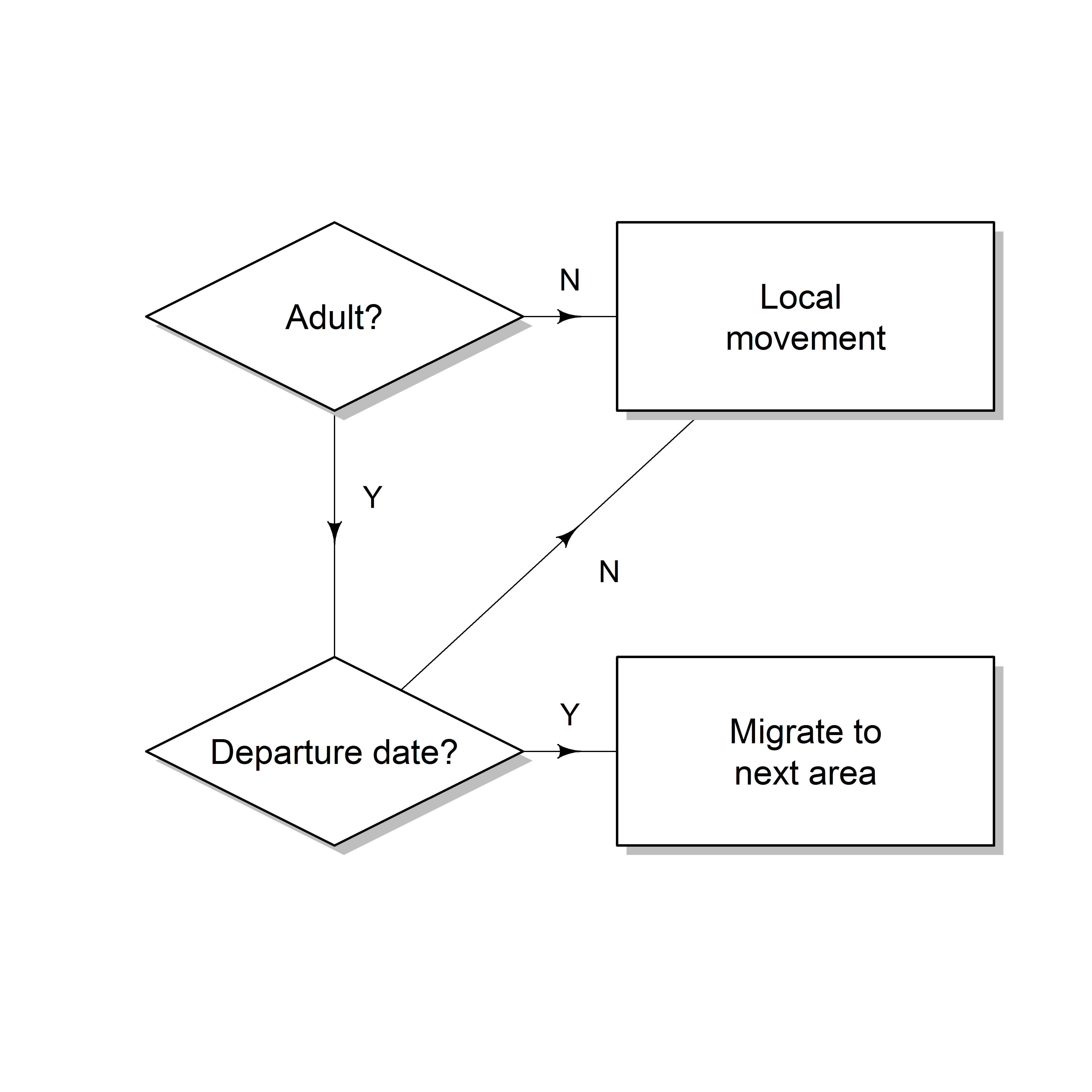


Figure 4. Conceptual movement algorithm. Local movement differs between different areas and at different times (e.g. spawning period and overwintering, see text for full details).

##### Migrations

Adults cycle between overwintering, spawning and feeding areas (Uriarte et al. 2001) (see state variables and scales). Migration departure dates were approximated from Uriarte *et al.* (2001) and Petitgas *et al.* (2010) and imposed at: October 1^st^ for the overwintering migration; February 1^st^ for spawning; and May 1^st^ for the feeding migration (Fig. 5). While this scheme captures the general pattern of NEAM migration, it should be noted that in reality migration timings can vary between years (Jansen and Gislason 2011).


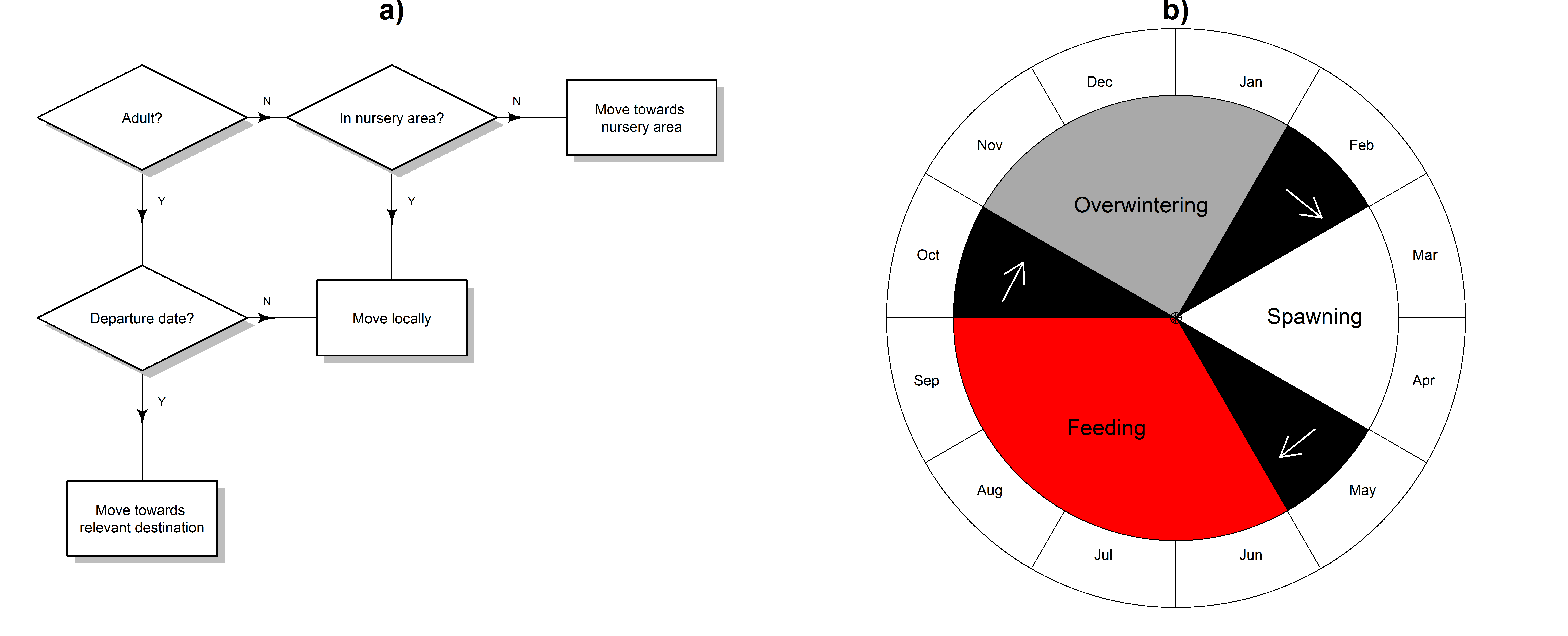


Figure 5. Broad summary of the annual cycle of adult locations in the SEASIM-NEAM, with black segments indicating when migrations occur (which do not necessarily last the whole month)

Once a migration is triggered, adults move from their current area towards the relevant destination. For each migration there is a destination patch corresponding to the entrance to the relevant area. The destination for the feeding and return overwintering migrations are at the entrance to the feeding area in the Faroe Shetland channel (northernmost red patch on Fig. 1). For the spawning migration the destination patch is located in the southern region of the spawning area (southernmost red patch on Fig. 1). We give each patch that is not on land an index R corresponding to its distance from the destination patch, while accounting for the fact that individuals cannot move over land. Once a migration is triggered (see Fig. 5 for dates), individuals move towards the appropriate destination to a patch with the lowest R within their possible search area. An individual’s possible search area is calculated from its minimum swimming velocity V_min ­_(km hr^-1^):

|  | $V_{min}=a_{v} L^{b_{v}}{A_{r}}^{c_{v}}$ | (2) |
| --- | --- | --- |

where a_v_ is a normalizing constant, L is body length, A_r_ is the caudal fin aspect ratio, and b_v_ and c_v_ are scaling exponents (see Table 2 for a full list of parameters and TRACE section 8 for a local sensitivity analysis). This means that larger individuals arrive earlier in destination areas, which has been noted for *S. Scombrus* (Jansen and Gislason 2011).

The spawning and feeding migrations are slightly more complicated than the overwintering migration, as they occur primarily along the European shelf edge to the west of the British Isles (Walsh et al. 1995, Brunel et al. 2017). We represent the shelf edge with a corridor around the British Isles in which 550m < depth < 50m (Fig. 1). For the spawning and feeding migrations we then add the constraint that individuals must remain on the shelf edge while moving to minimise R.

##### Local movement

There are three types of local (non-migratory) movement in SEASIM-NEAM: 1) adult foraging in the summer months; 2) random movement constrained to a particular area type (e.g. juveniles in the nursery area, adults in overwintering area in deep winter months); and 3) gradual northward movement on the spawning grounds as suitably warm regions open up for egg development in spring. The details of each type of local movement are given below.

###### Adult foraging

After reaching their destination at the end of the feeding migration, adults begin to seek out the most profitable patches on which to feed. Each patch is characterised by a profitability cue c_dd_ which is proportional to potential ingestion rate in that location. c_dd_ represents the bottom-up effect of phytoplankton density as a proxy for food availability, a density-dependent effect of intraspecific competition, an effect of photoperiod (as NEAM are primarily visual feeders), and an effect of SST (Kelvins), in the form of a Beddington-DeAngelis functional response:

|  | $c_{dd}=A\left( SST \right) p_{photo} \frac{X}{X+h+cD}$ | (3) |
| --- | --- | --- |

where X is phytoplankton density (g m^-2^), h is a half saturation constant, p_photo_ is photoperiod (as a proportion of 24 hours) at the SI’s location, D is local mackerel density (g patch^-1^), c determines the strength of the density dependence, and A(SST) is an Arrhenius function giving the effect of SST. A(SST) is given as:

|  | $A\left( SST \right)=e^{\frac{-E_{a}}{K}\left( \left( \frac{1}{SST} \right)-\left( \frac{1}{T_{ref}} \right) \right)}$ | (4) |
| --- | --- | --- |

where E_a_ is an activation energy, K is Boltzmann’s constant and T_ref_ is an arbitrary reference temperature.

SIs move in search of the most profitable locations (equation 3) at which to feed following a gradient area search (GAS). The GAS algorithm is broadly similar to that presented by Politikos et al. (2015), Tu et al. (2012) and Boyd et al. (2020). SIs can detect the profitability of the four neighbouring patches in x and y dimensions. Positions are updated five times per time step (i.e. once per day) to ensure that SIs cannot overshoot the neighbouring patch. Positions in x and y dimensions are updated in continuous space, as:

|  | $x_{t+1}=x_{t}+\left( D_{x}+R_{x}+C_{x} \right)$  $y_{t+1}=y_{t}+\left( D_{y}+R_{y}+C_{y} \right)$ | (6) |
| --- | --- | --- |

where D_x_ and D_y_ denote directed movements towards the most profitable patches, R_x_ and R_y_ denote random movements, and C_x_ and C_y_ are displacements caused by zonal and meridional horizontal currents, respectively.

In the orientated component of eq. (5) D_x_ and D_y_, SIs compare the profitability at their current location with that of the day before. If it has become more profitable, they will continue to swim in the same direction as the directed component of their movement the day before. If an SI’s current environment is less profitable than the day before, they follow a gradient search towards what is perceived to be the most profitable patch based on information in x and y dimensions, at realised velocity V_r_, given by:

|  | $D_{x}=V_{r} \frac{g_{x}}{\sqrt{{g_{x}}^{2}+{g_{y}}^{2}}}$  $D_{y}=V_{r} \frac{g_{y}}{\sqrt{{g_{x}}^{2}+{g_{y}}^{2}}}$ | (7) |
| --- | --- | --- |

where g_x_ and g_y_ are the gradients of the profitability cues (eq. 3) in x and y dimensions. V_r_ is equivalent to V_min_ (equation 2) plus some random noise, as V_r_ = V_min_ + (V_min_ ε), where ε is drawn randomly from a uniform distribution ranging from zero to one. The directed component of the GAS algorithm amounts to what is called a state-location orientation mechanism (basing new orientation on a comparison of the current and previous environment), and there is some indication that herring follow a similar strategy in the Norwegian sea (Fernö et al. 1998).

Following Politikos et al. (2015a) we assume that movement is directed (D_x_, D_y_) for 12 hours day^-1^, and movement in the other 12 hours follows the random component of eq. 5, R_x_, R_y_, given as moving at velocity V_min_ in a random direction that is not southward. Random southward movement is not permitted because acoustic studies have shown that NEAM infrequently swim southwards over summer (Nøttestad et al. 2016). However, SIs may still move southward during the oriented component of the GAS algorithm (i.e. if feeding conditions are best on a southward patch), or due to currents. R_x_ and R_y_ introduce stochasticity into the GAS models and prevent unrealistic overcrowding on optimal patches.

The effects of horizontal currents on SIs’ locations, C_x_, C_y_, are given as zonal (u) and meridional (v) current velocities (km hour^-1^), respectively, multiplied by the time step (here 24 hours as the GAS model operates five times per 5 day time-step).

NEAM avoid areas in which temperature is below 7⁰C (Ólafsdóttir et al. 2018). To reflect this, SIs are deterred from moving to patches on which SST is below this threshold. In the directed component of eq. 5, we repel individuals from patches with SST < 7°C by setting profitability cues in those areas to 0. For the random component of eq. 5, if a SI’s orientation would direct it on to a patch with SST < 7°C, its heading is reversed. If currents displace individuals on to an intolerably cold patch (or land) then this movement is abandoned and the SI instead moves to the centroid of the nearest suitable patch.

The energy cost associated with the GAS algorithm is subsumed in to a SI’s active metabolic rate (see section for details).

###### Random movement constrained to particular area types

Movement for adults in the overwintering area, and juveniles in the nursery area, follows a true random walk: They each move to a randomly-selected patch within their possible search area and the same area type each time-step (see migrations and eq. 1).

###### Spawning movement

Spawning begins on March 1^st^ and lasts for 60 days (Johnson 1977, Eltink 1987, Watson et al. 1992). This period covers peak spawning in 2007 and 2010 as observed in the triennial mackerel egg survey (MEGS) (ICES 2014). Throughout spring NEAM gradually progress northwards towards the feeding area as warming opens up suitable spawning habitat at higher latitudes (Dawson and Lockwood 1986, Eltink 1987). To reflect this, after spawning a batch of eggs, SIs move to the nearest patch north of their current location on which 10⁰C < SST < 12⁰ C (preferred spawning temperature). If there are no patches northwards in which 10⁰ C< SST < 12⁰C, SIs move to a random neighboring patch within that temperature range.

#### Bioenergetics

The following sub-models describe the energy budgets of larvae, juveniles and adults in terms of individual physiology. We assume eggs and yolk-sac larvae have sufficient energy to satisfy maintenance and maximal growth/ development.

##### Prey availability

SIs can cannibalise other individuals which are: 1) located on the same patch at the same time; 2) $\geq$3.5 times smaller (as in Shin & Cury 2001); and 3) < 0.33cm (Bachiller and Irigoien 2013). If multiple individuals satisfy these conditions then one is chosen at random to be preyed upon. The energy content of prey depends its fat content (as a proportion of its total mass). Lipid has an energy content E_lipid_ (kJ g^-1^) which is higher than the energy content of structural mass (1- lipid content, E_flesh_). At most times individuals do not overlap with potential mackerel prey and instead eat phytoplankton, which we use as a proxy for baseline food availability, with energy content E_p_ (kJ g^-1^).

##### Ingestion and energy uptake

Adults fast from November until after spawning the following year. Otherwise, ingestion rate IR is given as a function of both predator and prey density. This is described by a Beddington-DeAngelis functional response (Beddington 1975, DeAngelis et al. 1975), relating IR to body surface area (M^2/3^) (Kooijman and Metz 1983) and SST (kelvin), as:

|  | $IR=A\left( SST \right) C_{max}\frac{X}{X+h+cD}M^{2/3}$ | (8) |
| --- | --- | --- |

where IR is in g time-step^-1^, C_max_ is the maximum ingestion rate (g time-step^-1^ g^-1^ mackerel), h a the half saturation constant (g m^-2^), X is phytoplankton density (g m^-2^), D is local mackerel density (g patch^-1^) including the density of the focal individual, c determines the strength of predator density dependence and A(SST) is an Arrhenius function giving the effect of SST (eq. 3). If potential mackerel prey are available (see conditions above), then a proportion of IR, IR_cannibalism_, comprises mackerel prey (see *mortality* later in this section for the associated predation mortality). A justification for IR_cannibalism_ is provided in TRACE section 3. The remainder of ingested prey, total IR multiplied by (1 – IR_cannibalism_), comprises baseline prey availability as proxied by phytoplankton. Ingestion rate is converted from g time-step^-1^ to kJ time-step^-1^ using the energy content of the relevant prey type (kJ g^-1^). A proportion of ingested energy, an assimilation efficiency A_e_, becomes available for allocation to the following vital processes.

##### Maintenance

Standard metabolic rate, SMR, the level below which an individual cannot survive (Fry 1971), is used as a baseline measure of maintenance. SMR scales with body mass and temperature, according to:

|  | $SMR=a_{SMR}M^{b_{SMR}}e^{-E_{a}/K SST}$ | (9) |
| --- | --- | --- |

where SMR is measured in kJ time-step^-1^, $a_{SMR}$ is a normalizing constant and $M^{b_{SMR}}$ is body mass (g) raised to a scaling exponent b_SMR_ (see TRACE section 2.3). SMR is increased to active metabolic rate (AMR, kJ time-step^-1^) when migrating or actively foraging, given by:

|  | $AMR=a_{AMR}M^{b_{AMR}}V^{c_{AMR}}e^{-E_{a}/K SST}$ | (10) |
| --- | --- | --- |

where a_AMR_ is another normalizing constant and V is swimming velocity (km hr^-1^). For this case study we calculated that AMR scales linearly with V, i.e. an exponent of 1 (see TRACE section 2.3).

##### Growth

*S. scombrus* growth has a different form and rate in the first growing season than in later life (Steven 1952, Yohannan 1979). Body length L (cm) at age t (days) in the first growing season is well described by the Gompertz function:

|  | $L_{t}=L_{1}e^{{-e}^{-k_{1}(t-t_{max})}}$ | (11) |
| --- | --- | --- |

(Sirnard et al. 1992, Gluyas-Millan et al. 1998, Cotano and Alvarez 2003, Villamor et al. 2004) where L_1_ is the maximum length at the end of the first growing season (cm), k_1_ is the maximum growth rate in the first season, and t_max_ is t (days) at which growth is maximum. k_1_ is adjusted for the SST at which it was recorded using the Arrhenius function. For older individuals the von Bertalanffy equation (von Bertalanffy 1938) is generally used:

|  | $L_{t}=L_{\infty}(1-e^{-k\left( t-t_{0} \right)})$ | (12) |
| --- | --- | --- |

(Villamor et al. 2001) where k is the Bertalanffy growth constant (time-step^-1^), $L_{\infty}$ is the asymptotic length (cm) and t_0_ is an adjustment parameter. k is adjusted for the SST at which it was recorded using the Arrhenius function. Taking the end of the first growth phase to be at t = 240 (days, see TRACE section 2.3), from equations 11 and 12 the maximum growth rate $\Delta L$(cm time-step^-1^) is given by:

|  | $\Delta L=\left\{ \begin{aligned} k_{1}e^{\frac{-E_{a}}{K}\left( \left( \frac{1}{SST} \right)-\left( \frac{1}{T_{ref}} \right) \right)} L \ln\left( \frac{L_{1}}{L} \right), t<240 \\ ke^{\frac{-E_{a}}{K}\left( \left( \frac{1}{SST} \right)-\left( \frac{1}{T_{ref}} \right) \right)}\left( L_{\infty}-L \right), t\geq240 \end{aligned} \right.$ | (13) |
| --- | --- | --- |

We assume that adults grow only when feeding (Petitgas et al. 2010), i.e. for half of the year. To reflect this, their value of k obtained from equation 12 is doubled. ∆L (cm time-step^-1^) is converted to the difference in structural mass ∆M (g time-step^-1^) assuming an allometric relationship between L and structural body mass M_struct_:

|  | $M_{struct}=a_{w}L^{b_{w}}$ |  | (14) |
| --- | --- | --- | --- |

where a_w_ is a normalizing constant and b_w_ is a scaling exponent. We define structural mass as total body mass minus lipid stores and gonads. Growth costs are calculated using $\Delta M \left( E_{c}+E_{s} \right)$, where $E_{c}$ is the energy content of flesh (kJ) and $E_{s}$ is the energy costs of synthesising flesh (kJ g^-1^). If insufficient energy is available to support maximum growth, the growth rate is reduced accordingly.

##### Reproduction

The maximum number of eggs that a female can produce, potential fecundity f_p_, is calculated at the beginning of the spawning period (Fig. 5) as a function of body length L, as:

|  | $f_{p}=a_{f}L^{b_{f}}$ |  | (15) |
| --- | --- | --- | --- |

where a_f_ is a normalizing constant and b_f_ is a scaling exponent. The energy cost of producing a maximum-sized batch of eggs b_max_ (kJ time-step^-1^) is then given as a function of f_p_, as:

|  | $b_{max}=\frac{{f_{p}M}_{0} (E_{c}+E_{s})}{n_{b}}$ |  | (16) |
| --- | --- | --- | --- |

where $M_{0}$ is egg mass, E_c_ is the energy content of flesh, E_s_ is the cost of synthesising tissue and n_b_ is the number of batches produced. NEAM are batch spawners, so energy is allocated to each batch over the inter-batch intervals b_int_. Hence, the duration of the spawning period is given by n_b_ multiplied by b_int_. If less energy than b_max_ is available, batch size is reduced accordingly. We define gonad mass as equal to the mass of the eggs produced in a batch. This increases as energy is allocated to a batch over b_int_, then is reset to zero when that batch is spawned. The egg production of all females is divided equally among n_cohort_ new individuals (eggs) each year. We assume that male and female investment in reproduction is equal.

##### Energy reserves

Larval mackerel prioritse growth (Peterson and Ausubel 1984) over energy storage. Juveniles and adults store energy as lipid (Wallace 1991, Grégoire et al. 1992) in preparation for maturation, spawning and, for adults, the winter fast. Individuals can store energy up to their maximum possible energy reserve E_max_ (see TRACE section 2.3). The energy costs of synthesising lipid L_s_ are accounted for when assimilated energy is converted to energy stores. The mass of stored lipid and, for adults, the gonads are added to structural mass to get total mass M.

#### Egg development

While embryo duration in S. scombrus decreases with temperature, background mortality rate M_back_ increases. Hence, the cumulative proporion of eggs that die from M_back_ varies little except at extreme temperatures (Mendiola et al. 2006) (see TRACE section 2.4) not encountered in the model (see TRACE section 2.4). We therefore assume for simplicity that the egg development period is five days and M_back_ is constant at rate M_e_ (see Mortality).

#### Ontogenetic transformation

Eggs transform into yolk-sac larvae at length L_hatch_ once reaching the end of their development period. Thereafter individuals transform into larvae (cease to be nourished by the yolk sac) when they reach 0.61 cm (Studholme et al. 1999); into juveniles when they reach 3 cm (at which point *S.scombrus* have been observed to exhibit active taxis and schooling behaviour; Sette (1943)); and can sexually mature as adults after reaching 26.2 cm (L_mat_). For simplicity juveniles with a sufficient length all reach maturity on the same day each year, February 1^st^. At this point they join the adult migration towards the spawning area.

#### Mortality

The ways in which the abundance n of of an individual can decrease are as follows. Starvation: If an individual’s total mass reduces to its structural mass it dies.

Predation: If a SI is selected as prey for a larger SI, its abundance is reduced by M_pred_. M_pred_ is given as ingestion rate IR of the predator (g time-step^-1^) / prey body mass (g), after adjusting the predator’s IR by IR_cannibalism_. Hence, M_pred_ depends on the number of predators and SST.

Background mortality: Eggs and larvae are susceptible to background mortality at rate M_e_. Juvenile susceptibility to M_back_ at length L is given by:

|  | $M_{back}=M_{a}\frac{L_{mat}}{L}$ | (17) |
| --- | --- | --- |

where M_a_ is a constant equal to adult mortality susceptibility (time-step^-1^), L_mat_ is the threshold length above which juveniles can sexually mature and L is length (cm) (Brodziak et al. 2011). Because background mortality rates decrease with life stage or body length, cumulative mortality depends on growth.

Fishing mortality: Annual rates of fishing mortality rates F (time-step^-1^) are taken from the stock assessment (stockassessment.org). These rates are applied each day, such that the proportion being applied in each month is proportional to the historical proportion of annual catch in that month (Fig. 3).

M_back_ and F are converted to a proportion of a SI‘s abundance dying in a time-step as $1-e^{-(M_{back}+F)}$. SIs with abundance < 1 are removed from the model.

*Recruitment*

Default „emergent“ recruitment configuration

Recruitment is defined as the number of young-of-the-year that survive to December 31st each year. This depends on the total number of eggs spawned, and the fraction of those eggs that survive. The number of eggs spawned is determined by the amount of energy that the spawning stock is able to accumulate prior to spawning, which reflecs the feeding opportunities available over the previous summer. The fraction of eggs that survive to the end of their first year depends largely on the previling environmental conditions on the spawning grounds. Mortality rate is inversely related to body size meaning that, if conditions favour quick growth (e.g. high prey availability and temperature), then cumulative mortality in that cohort is reduced and more SIs recruit.

#### Ricker-style stock recruitment configuration

Users can choose to substitute the 'emergent“ recruitment scheme for a more traditional Ricker-type stock-recruitment-environment (SRE) relationship that has been fitted to data from the stock assessment. The SRE is a modified Ricker function, giving recruitment R as a function of SSB and SST, as:

|  | $R=a_{R}{SSB e}^{-b_{R} SSB+ c_{R} SST}$ | (18) |
| --- | --- | --- |

where a_R_ b_R_ and c_R_ are coefficients. For the SRE SST represents mean SST on the spawning grounds (see section 3) over the core spawning period (March, April).

Use of the SRE configuration removes the early life stages from the IBM. Instead of being introduced as eggs at spawning time, n_cohort_ new individuals are introduced at the end of each year with abundance = R / n_cohort_. Recruits‘ body lengths are set at the maximum length at the end of the first growing season L_1_ (cm) minus some random noise, given by L_1_ – (ε 3), where ε is a random number between 0 and 1. The energy reserves of recruits are set at one half maximum, and from this all other state variables are calculated in the simulation. At the end of the year the new recruits are distributed randomly in the nursery area.

# Data evaluation

**This TRACE element provides supporting information on**: The quality and sources of numerical and qualitative data used to parameterize the model, both directly and inversely via calibration, and of the observed patterns that were used to design the overall model structure. This critical evaluation will allow model users to assess the scope and the uncertainty of the data and knowledge on which the model is based.

Summary:

**In this section we first give details of how some of the model’s parameters were estimated from data in the literature. We then show how three parameters were estimated inversely from mackerel population data using approximate Bayesian computation (ABC). Finally, we give details of the model’s input data and how it was processed.**

## Parameter derivations

### Parameters from the literature

#### Parameters of the Ricker stock-recruitment-environment relationship

The Ricker stock-recruitment-environment relationship SRE (equation 15) gives recruitment per tonne of SSB as a function of SSB and SST on the spawning grounds at spawning time. Data used to fit the SRE includes 28 years (1990 – 2018) of recruitment, SSB and SST on the spawning grounds at spawning time. The spawning area is defined as the enhanced area for spawning sampling NEAM eggs in the MEGS egg survey (ICES 2014) (Fig. 7 , right hand panel). Recruitment and SSB were taken from the 2019 NEAM stock assessment (see stockassessment.org). SST was averaged over March and April (spawning time). The SST data was taken from NOAA’s Optimum Interpolation Sea Surface temperature 2.0.0 dataset ([https://www.esrl.noaa.gov/psd/data/gridded/data.noaa.oisst.2.0.0.html](https://www.esrl.noaa.gov/psd/data/gridded/data.noaa.oisst.v2.html)). The reason that the SST data used here differs from that used to force the IBM is that the satellite-derived estimates are not available sufficiently far back in time to fit the SRE. Fig 7. shows recruitment per tonne of SSB as a function of 1) SSB and 2) SST on the spanwing grounds at spawning time.


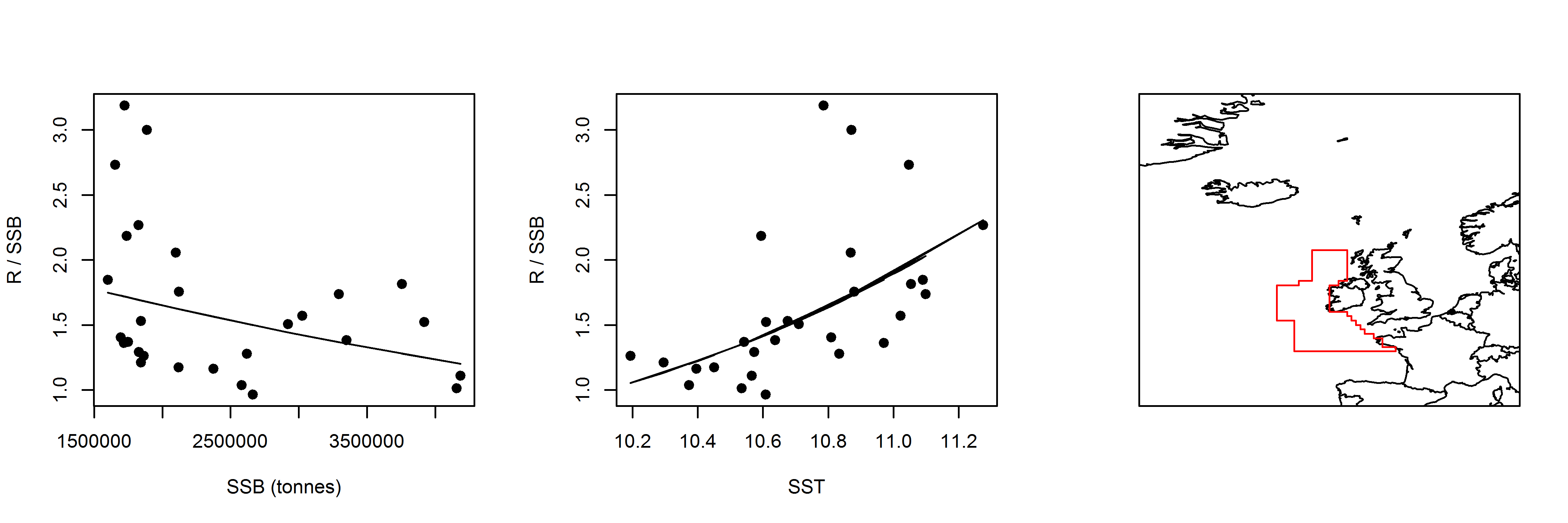


Figure 7. Recruitment per tonne of SSB (from the 2019 NEAM stock assessment) plotted as a function of 1) SSB (left panel) and 2) SST (middle panel) in the spawning area at spawning time. Fitted curves are the standard Ricker model for SSB and the Arrhenius function for SST. The right hand panel shows the spawning area over which SST is averaged.

As is standard, we estimated the parameters of the SRE using log-linear regression. Taking logarithms of equation 18 gives:

$$\ln\left( \frac{R}{SSB} \right)=-6.79-0.0000001323 SSB+0.7019 SST$$

The intercept, -6.79, must be exponentiated for use in equation 18, giving 0.001148843. In all, the parameters of the SRE are: a_R_ = 0.001148843, b_R_ = 0.0000001323, and c_R_ = 0.7019. See Fig. 8 for diagnostics of the fitted SRE.


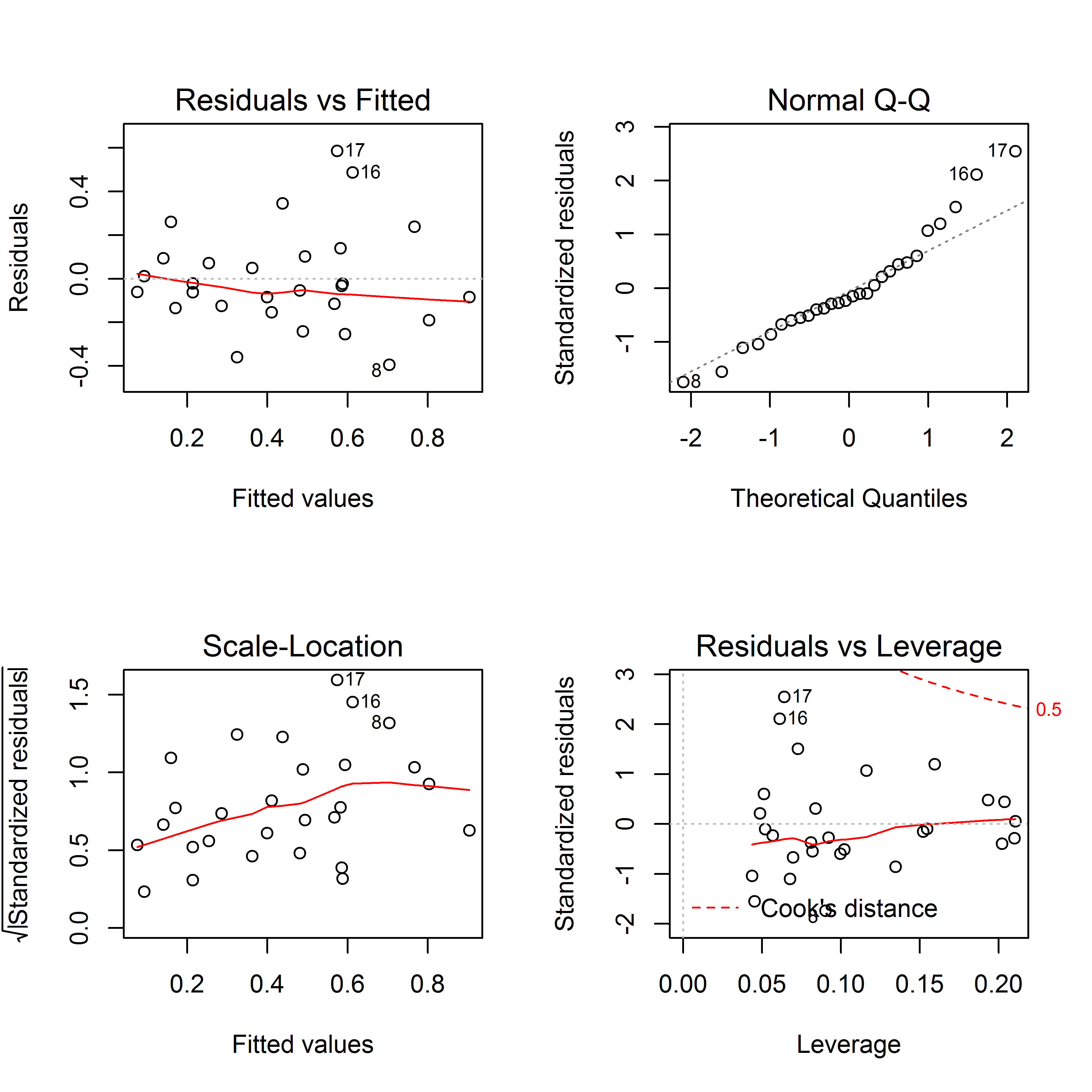


Figure 8. Diagnostics for the Ricker stock-environment-recruitment model (eq. 15).

To assess the relative importances of SSB and SST to recruitment, we compared the fits of three log-linear regression models: 1) a standard Ricker model after taking logarithms (i.e. ln(R/SSB) = a * SSB, as in equation 18); 2) log-linear regression of ln(R/SSB) on SST; and 3) the full SRE (ln(R/SSB) = a * SSB + b * SST). The full SRE provides the best fit, both in terms of R^2^ and AIC, the latter accounting for inclusion of an additional parameter (Table). Interestingly, model 2 provides a better fit than model 1, indicating that SST explains more of the variation in recruitment than does SSB. These results show that the full SRE should be used.

| Model | Coefficients | R^2^ | P value | AIC |
| --- | --- | --- | --- | --- |
| SSB | Intercept: 7.921e-01, p=0.00017  SSB: -1.453e-07, p = 0.04912 | 0.141 | 0.04912 | 16.39784 |
| SST | Intercept: -7.3382, p=0.00097  SST: 0.725, p=0.0005 | 0.3741 | 0.0005438 | 7.527512 |
|  |  |  |  |  |
| SSB + SST | Intercept:-6.79, p=0.0010  SSB: -1.323e-7, p=0.025  SST: 7.02e+1, p=0.00035 | 0.45 | 0.00022 | 3.77 |
|  |  |  |  |  |
|  |  |  |  |  |

#### Maximum ingestion rate C_max_

C_max_ was estimated using data on the daily ration of Pacific mackerel (*Scomber japiconus*) of average body mass 97 g (Hatanaka et al. 1957), fed to satiation at a temperature of 294.15 k. These individuals had an ingestion rate of 1.28 g day^-1^ g^-1^ of average body mass 97g. Substituting into equation 8 with a reference temperature T_ref_ of 285.15 k, gives a C_max_ of 0.69 g day^-1^ g^-1^ fish.

#### Cannibalism as a proportion of ingestion rate IR_cannibalism_, and the threshold size above which individuals cannot be cannibalised L_cannibalism_

Cannibalism represents a significant source of mortality for young-of-the-year NEAM (Fortier and Villeneuve 1996, Hillgruber and Kloppmann 2001, Mendiola et al. 2007). In our IBM juveniles overlap with eggs and larvae after spawning. Using stomach content data from Cefas’ DAPSTOM database (Pinnegar 2014), we calculated that, for juveniles (defined as less than 30cm) in the Celtic Sea, Irish Sea and West of Scotland, mackerel eggs and fish larvae comprise 6.4% of NEAM diet. As such we set the rate of cannibalism as a proportion of ingestion at 0.064. Note that in the IBM IR_cannibalism_ only applies if the focal SI overlaps spatially with a potential prey SI (i.e. a sufficiently small individual). To our knowledge, data are not available on the size at which individuals cease to be susceptible to cannibalism in our study region. However, in the Bay of Biscay, mackerel will not eat anything > 0.33cm (Bachiller and Irigoien 2013). We therefore set L_cannibalism_ at 0.33cm. It should be noted that another category in the DAPSTOM database, unidentified fish remains, could contain mackerel. However, given that mackerel will not eat anything above 0.33cm, we feel it is legitimate to assume that this will be negligible.

#### Normalizing constant for standard metabolic rate aSMR

S_0_ was calculated from data on the oxygen consumption of horse mackerel (*Trachurus trachurus*) at different body masses (Herrmann and Enders 2000). O_2_ consumption was converted to metabolic rate using an oxycaloric conversion factor of 13.6 kJ g^-1^ 0_2_ (Elliott and Davison 1975, Brett and Grove 1979, Bernreuther et al. 2013). Log-linear regression gave:

$$\ln\left( SMR \right)=-2.66+0.75 ln(M)$$

where SMR is measured in kJ day^-1^ and body mass M in grams. Evaluating at 1g gives SMR = 0.07. From equation 9, S_0_ = 0.07 $e^{E_{a}/K SST}$ = 0.45x10^8^, where T is the experimental temperature in kelvins (286.15).

#### Normalizing constant for active metabolic rate a_AMR_

A_0_ for active metabolic rate (AMR) was calculated from data on the respiration of *S. japiconus* of different body masses, M, swimming at different velocities V (Dickson et al. 2002). Metabolic rate was obtained from respiration as above. Multiple regression gave:

$$ln\left( AMR \right)=-1.6+0.75 ln\left( M \right)+ln\left( V \right)$$

where AMR is in kJ day^-1^, M in g and V in km hour^-1^. Evaluating at a mass of 1g and a speed of 1 km hour^-1^ gives AMR = 0.2. From equation 10, A_0_ = 0.2 $e^{E_{a}/K SST}$= 8.86x10^7^, where T is the experimental temperature in kelvins (291.15).

#### Bertalanffy growth constant k

Values of k for *S. scombrus* from areas within the range of the western stock (North, Irish and Cantabrian seas and the English channel) were taken from FishBase, along with the corresponding temperature. As recommended by FishBase we only included values for which the corresponding L_∞_ was greater than 2/3 maximum observed length (60cm), i.e. 40 cm. Each estimate was corrected to the reference temperature using the Arrhenius function, giving a range in k of 0.22 to 0.47. We took k to be the mean of the remainder, 0.314 year^-1^.

#### Asymptotic length L∞

Using the same subset of data used to estimate k from FishBase, we took the mean of the corresponding L_∞_ values, which was 42.4 cm.

#### Gompertz growth constant k1

Villamor et al. (2004) estimated a k_1_ of 0.038 (day^-1^) for *S. scombrus* in the Cantabrian sea. We used NOAA’s Optimum Interpolation SST (NOAA 2017) to estimate an average SST of 290.91 k over the temporal and spatial extent of their study. We could not use the same SST data from MODIS-Aqua because it was not operational at the time. We then corrected Villamor, Bernal and Hernandez's (2004) k_1_ to 0.025 at T_ref_ using the Arrhenius function.

#### Energy cost of synthesising lipid Els

The energy density of lipid is about 39.3 kJ g^-1^ (Schmidt-Nielsen 2013). Subtracting this from the energetic cost of storing lipid given by Pullar and Webster (1977), 54 kJ g^-1^, gives the cost of synthesis, 14.7 kJg^-1^.

#### Parameters of the weight-length relationship

In the model body length is converted to structural body mass using the standard allometric relationship W=aL^b^. We obtained the relationship between standard (or average) mass and length by averaging estimates of a and b from each month of the year (except June where there was no data), using data from German survey and commercial catches (Wilhelms 2013). This gave standard mass = 0.00285 L^3.325^. Because we assume that structural mass is 0.76 x standard mass (see *minimum and maximum lipid reserves*), we then adjusted the constant a by a factor of 0.76 to get structural mass = 0.0022 L^3.325^.

#### Threshold length for maturity, Lmat

L_mat_ was determined using estimates of the median length at which *S. scombrus* become mature for the first time (Lm) from FishBase. We used the lowest Lm for the western stock to be L_mat_, which was 26.2 cm.

#### Minimum and maximum energy reserves E_max_

The amount of lipid an individual can store (g) was derived from data on the condition factor C_f_ (100 x mass (g) /length (cm) ^3^)) of *S. scombrus* at different stages of maturity (Grégoire et al. 1992, table 6). The mean C_f_ across all maturity stages in 1991 and 1992 was 1. We therefore considered an individual at standard mass (standard mass for its length, as obtained from the weight-length relationship above) to be at C_f_ =1. The lowest C_f_ was 0.76, or 76% of standard mass. We therefore took standard mass x 0.76 to be structural mass (i.e. where lipid reserves are empty). The mass of any lipid stored is added to structural mass (and gonad mass if in the spawning period) to get total mass. The maximum C_f_ in (Grégoire et al. 1992, table 6) was 1.35. We assume that this corresponds to an individual with full lipid reserves. Therefore, the maximum amount of lipid that can be stored is equivalent to the range in C_f_ from Grégoire et al. (1992), 0.59, multiplied by standard mass (where C_f_ = 1). Because we assume structural mass is 0.76 x standard mass, the maximum amount of lipid that can be stored is 0.78 x structural mass.

#### Age when growth changes form Gthresh

The age when growth switches from the Gompertz to von Bertalanffy form was taken to be the age at which the premature and mature growth curves meet for Indian mackerel (*Rastrelliger kanagurta*) in Fig. 5 of Yohannan (1979), ~ 240 days. This roughly corresponds to the point at which length at age in young-of-the-year *S. scombrus* reaches an asymptote (Cotano and Alvarez 2003), i.e. when they reach the end of their first growth phase in their first winter.

### Calibrated parameters

We recalibrate SEASIM-NEAM periodically as new data become available (e.g. new stock assessment estimates of SSB, for example). In this application, we calibrated three of SEASIM-NEAM’s parameters: the half saturation constant (h), the background early mortality rate (M_e_) and the strength of intraspecific competition (c). To estimate these parameters, SEASIM-NEAM was fitted to data on weight-at-age and SSB (stock assessment estimates). The IBM was fitted using rejection approximate Bayesian computation (ABC), generally following the methodology of van der Vaart et al. (2015). This involved running the model 2000 times, while randomly drawing values of the parameters from uniform prior distributions (see below for the ranges of these priors). To determine which parameters gave the best fits we calculated the distance d between the model outputs and the data, as:

|  | $d(m_{i},D)=\sqrt{\sum_{j} \left( \frac{m_{i,j}-D_{j}}{mad(m_{j})} \right)^{2}}$ | (19) |
| --- | --- | --- |

where m_i,j_ is simulation i’s output for data point j, D_j_ is the empirical data for data point j, and mad(m_j_) is the median absolute deviation from the median of output j in all model runs. mad(m_j_) is used to normalize the different scales of the data so that the distance calculations are not affected by the different units used in the datasets. We gave the SSB data 15 times the weighting of the weight-at-age data because: the SSB estimates are based on a substantial amount of data in the stock assessment (e.g. catches, recruitment index, SSB index, egg production index). We “accepted” the parameters from the simulations that resulted in the best 1.5% of fits to the data, giving an approximation of the posterior parameter distributions. See section 6 for a comparison of predicted and observed SSB and weight-at-age.

#### Rationale for calibrating parameters and choice of prior distributions

##### Background early mortality M_e_

Initially we considered background egg and larval mortality rates as separate parameters, but it was difficult to calibrate them because naturally they were strongly correlated. Instead, we combined them into one parameter, early mortality M_e_. The reasons we calibrate M_e_ are (1) there is a large range of estimates of egg and larval mortalities in the literature (at least 0.045 to 0.42 day^-1^); (2) these estimates are very uncertain due to difficulties in measuring this parameter; and (3) it is the parameter to which the model is most sensitive (see section 7). We chose a prior distribution of 0.045 to 0.36 for M_e_ in Boyd et al. (2018). The reason that the prior distribution does not span the entire literature range is that values above 0.36 can result in population collapse. More recently have further refined the lower bound of the prior distribution to be 0.25 because values below, or even near to, this were never accepted. So, the prior distribution for M_e_ spans 0.25 to 0.36.

##### Strength of the predator density dependence c

We calibrated the parameter c because we could find no data from which it could be estimated independent of the IBM. The prior distribution of c spans 0 (i.e. no competition effect) to an intentionally high bound of 10^-10^.

##### Half saturation constant h

It was not possible to estimate h independent to the model for two reasons: 1) the only data on feeding rate as a function of food density that we could find was over an experimental period of just 5 minutes (Pepin et al. 1988); and 2) because we use phytoplankton as a proxy for the amount of food available, but it is not an actual prey type for *S. scombrus*. In Boyd et al. (2018) the data we used for calibration did not prove informative as to the value of h, so we fixed the value at 1.3 g m^-2^. Since then, incorporation of weight-at-age data in the ABC has enabled estimated on h. We therefore calibrate h and, based on initial testing of parameter values, set the bounds of the prior distribution at 0.65 – 1.3 g m^-2^.

#### ABC results

ABC was able to estimate each of the three parameters, as indicated by posterior distributions that are significantly narrower than the priors (Levene’s test, p < 0.5, Fig. 10).


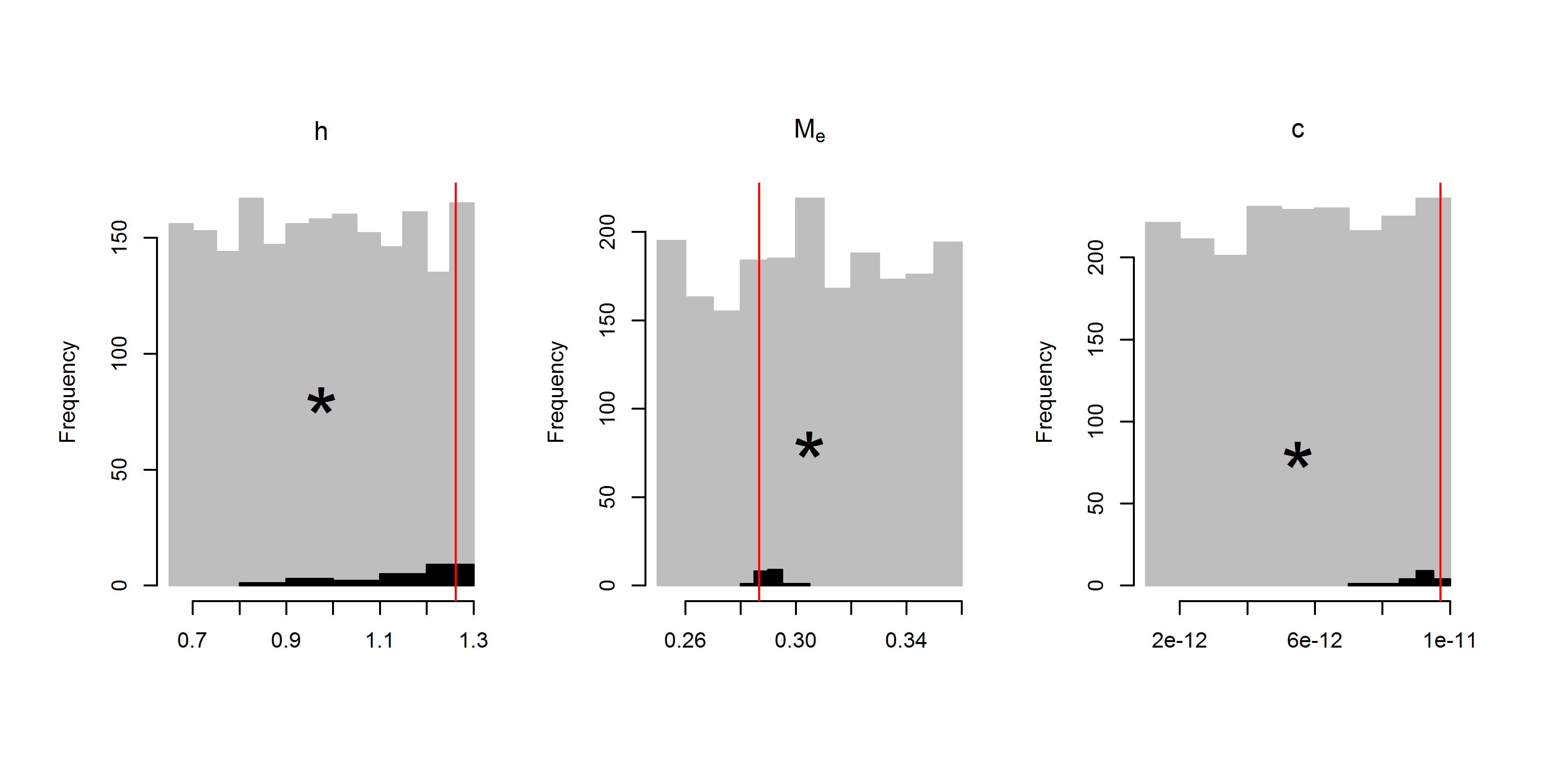


Figure 10. a) Histograms of the prior (grey bars) and posterior distributions (black bars) of the parameters M_e_, h and c. Vertical red lines indicate the parameters that resulted in the best-fitting simulations, and asterisks indicate that the posterior distribution was significantly narrower (lower variance) than the prior (Levene’s test, p<0.05).

## Input data

### Fishing mortality

Instantaneous rates of fishing mortality-at-age F (day^-1^) for each year are taken from the 2019 NEAM stock assessment (ICES (2019b) obtained from stockassessment.org). These rates are estimated using a state-space assessment model fitted to data on catches and various population indices. Unless testing management scenarios (see section 2), fishing mortality is not spatially-explicit but rather is applied uniformly to all individuals within an age group. Annual rates of F are taken from the stock assessment, but are weighted to represent the historical proportion of catches taken in each month (see table 3.)

### Environmental inputs

#### Chlorophyll and SST

Environmental inputs comprise dynamic maps of chlorophyll-a concentration, SST, bathymetry, photoperiod (as a proportion of 24 hours) and horizontal current velocities. Users can select whether they want to use inputs derived from satellite remote-sensing, or, if they would like to run the model into the future, to use estimates from the ESM GFDL-ESM-2M (Dunne et al. 2013). When using ESM inputs, users have the option to change the year in which the simulation starts (1981 at the earliest). However, when using remote sensing inputs users cannot change the start year: the spin up begins in 1995, lasts for ten years and the simulation begins in 2005.

The satellite remote-sensing data were taken from NASA’s ocean colour portal in NetCDF format (NASA 2017, NASA OBPG 2017). Processing of the satellite data included: 1) cropping to the model extent; 2) estimating missing values using linear temporal interpolation; 3) re-projecting on to the Lambert Azimuthal equal area projection using bilinear interpolation; 4) upscaling the spatial resolution from 9km x 9km to 60km x 60km using nearest neighbour interpolation (default NetLogo method); and 5) converting the files to ESRI ascii format for use in NetLogo. For the few pixels without a value of SST or chlorophyll after interpolation, annual averages were used. Most of the above steps are conducted using the R package raster (Hijmans 2019b). However, step 4 is implemented within NetLogo. This allows users to easily switch between high (9km) and low (60km) resolutions using an interface widget [although note some newer modules (see section 5.2) do not work on the finer resolution].

Although there are some gaps in the satellite remote-sensing data, the areas and times in which most interpolations are required are largely unimportant to the model. The majority of gaps in the data are found in the northern regions in the deep winter months. In our IBM individuals do not inhabit the Northern regions in these months. In fact, one of the more important outputs of our IBM is the summer feeding distribution when the satellite data has highest availability. The IBM is also highly sensitive to the environmental conditions that larvae experience after hatching. Larvae hatch in the southern region of the model extent in spring, again when few interpolations are required.

As an alternative to the satellite remote-sensing data, users can choose to use estimates of SST and chlorophyll from the ESM GFDL-ESM2M. GFDL-ESM-2M was chosen because: 1) it includes a highly resolved biogeochemical formulation and correlates relatively well with global net primary productivity data; and 2) because model drift is negligible (Tittensor et al. 2018). For each ESM users can choose from the extreme Representative Concentration Pathway (RCP) scenarios 2.6 and 8.5, i.e. low and high greenhouse gas emissions scenarios, respectively.

ESM forcing datasets for the IBM were generated by combining historical (Jan 1981 – Dec 2005) ESM outputs with RCP scenario-based projections out to 2050. A slight complication arises in that the historical period as defined for CMIP (phase 5 as used here) ends in December 2005, after which RCP scenario-driven estimates are produced from the ESMs. This does match with the historical period as defined in our IBM simulations (everything up to 2019). For this reason, from 2006 we had multiple environmental trajectories (one from each RCP) from which to choose as input to our IBM. Inspection of the environmental inputs revealed negligible divergence between fields of chlorophyll and SST out to 2019 from RCPs 2.6 and 8.5 (RMSEs of 0.31⁰C and 0.024 mg m^-2^, respectively; Fig. 11). For this reasons we simply took the mean of the environmental inputs from RCPs 2.6 and 8.5 as forcing to the IBM from 2006 to 2019. The data were obtained in NetCDF format from the coupled model inter-comparison project’s site at <https://cmip.llnl.gov/>. We use ESM data with a temporal resolution of one month. The default spatial resolution of GFDL-ESM-2M is 0.334 – 1° latitude (equator – poles), and constant 1° in longitude (<https://portal.enes.org/data/enes-model-data/cmip5/resolution>). The ESM spatial resolution was re-sampled and re-projected to match the IBM grid (60x60 km) using bilinear interpolation. Our code for re-gridding the ESM outputs was based on an excellent tutorial by Mark Payne of DTU Aqua (<http://rpubs.com/markpayne/132500>).


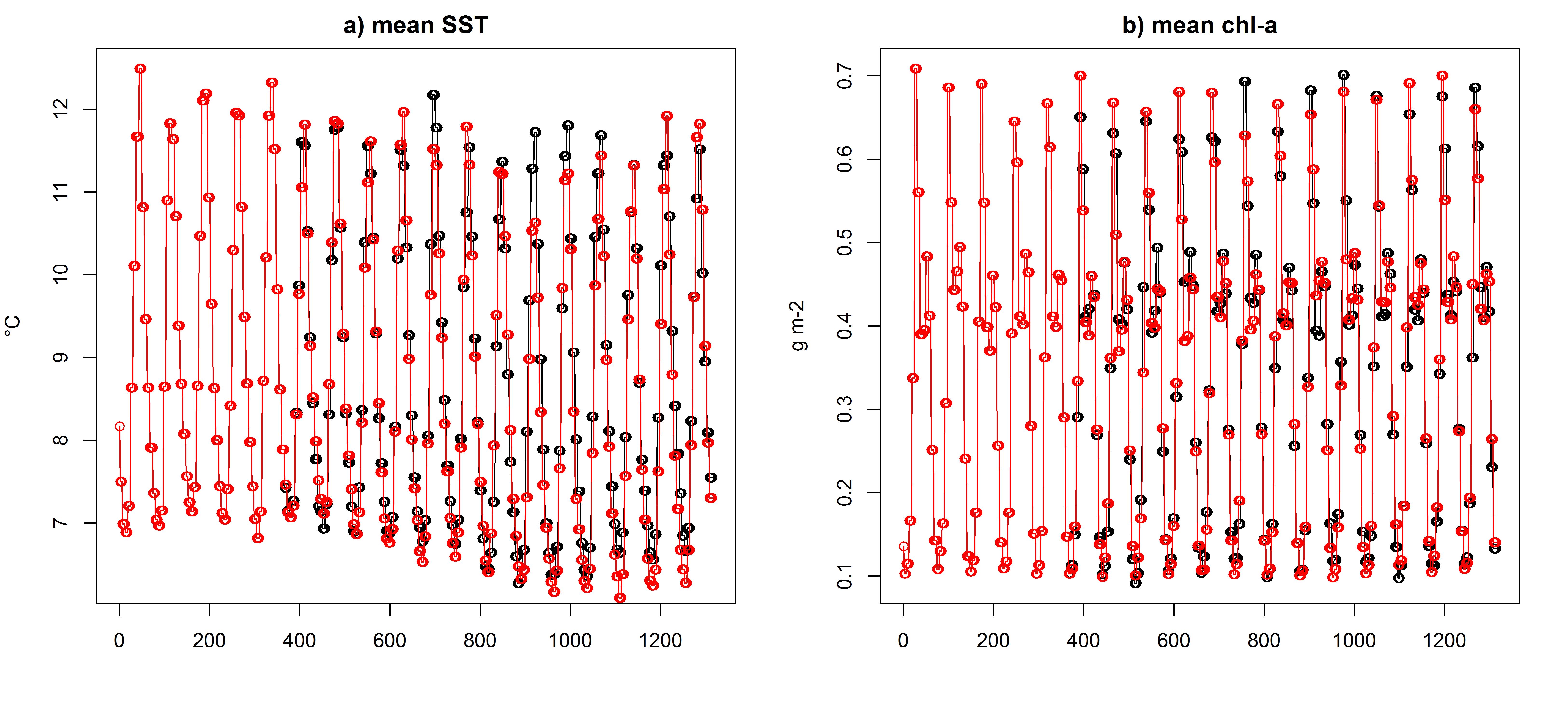


Figure 11. Comparisons of mean a) SST and b) chl-a over the IBM domain under the RCP scenarios 2.6 and 8.5. Units on the x-axis are time-steps since January 1st 2001 with the end of the time-series corresponding to December 31st 2018. Note that up to 2006 the time-series are identical as this is the „historical period“ as defined by CMIP. From this point the time-series‘ diverge, though not dramatically.

We obtained maps of phytoplankton biomass, which we use a proxy for prey available to the mackerel, from estimates of chlorophyll-a concentration. Chlorophyll-a concentration was converted to carbon weight using a Carbon:Chl-a ratio of 75. Then, a wet weight:Carbon ratio of 10 was used to obtain phytoplankton biomass (g wet weight m^-2^) (Link et al. 2006). This gives phytoplankton biomass (g m^-2^) = chl-a (mg m^-2^) x 0.75.

#### Bathymetry

Bathymetry is used to delineate the juvenile nursery areas, and the spawning area. Bathymetric data was obtained from the National Oceanographic Data Centre in NetCDF format (NODC 2017), at a spatial resolution of 30-arc second intervals. The resolution was re-sampled to match the other input data. All data was processed using the R package “raster” (Hijmans et al. 2016).

#### Horizontal currents

Near surface zonal (u) and meridional (v) current velocities (m sec^-1^) were obtained from the 1/3 ° OSCAR dataset (ESR 2009). The data were resampled and reprojected on to our model grid using bilinear interpolation. The data were processed using the projectRaster() function in the R package raster (Hijmans et al. 2016). The data were next converted from m sec^-1^ to km hour^-1^ using a multiplier of 3.6. Data were extracted for the months of May through September. No data were available for the selected months prior to 2012. For this reason, we calculated a mean climatology for each month based on the period 2012 to 2018, meaning that we do not account for inter-annual variability in current velocities. Current velocity vectors for the months of May through September can be seen in Fig. 12. A slight complication arises in that the OSCAR data represent current velocities in the true north and true west directions, as opposed to the grid north and grid west in the IBM. To circumvent this problem we calculated the Netlogo heading on each patch which corresponds to true north, and approximated the heading that corresponds to true west. It is then simple to calculate SIs’ displacements caused by currents in the NetLogo grid (see equation 6).


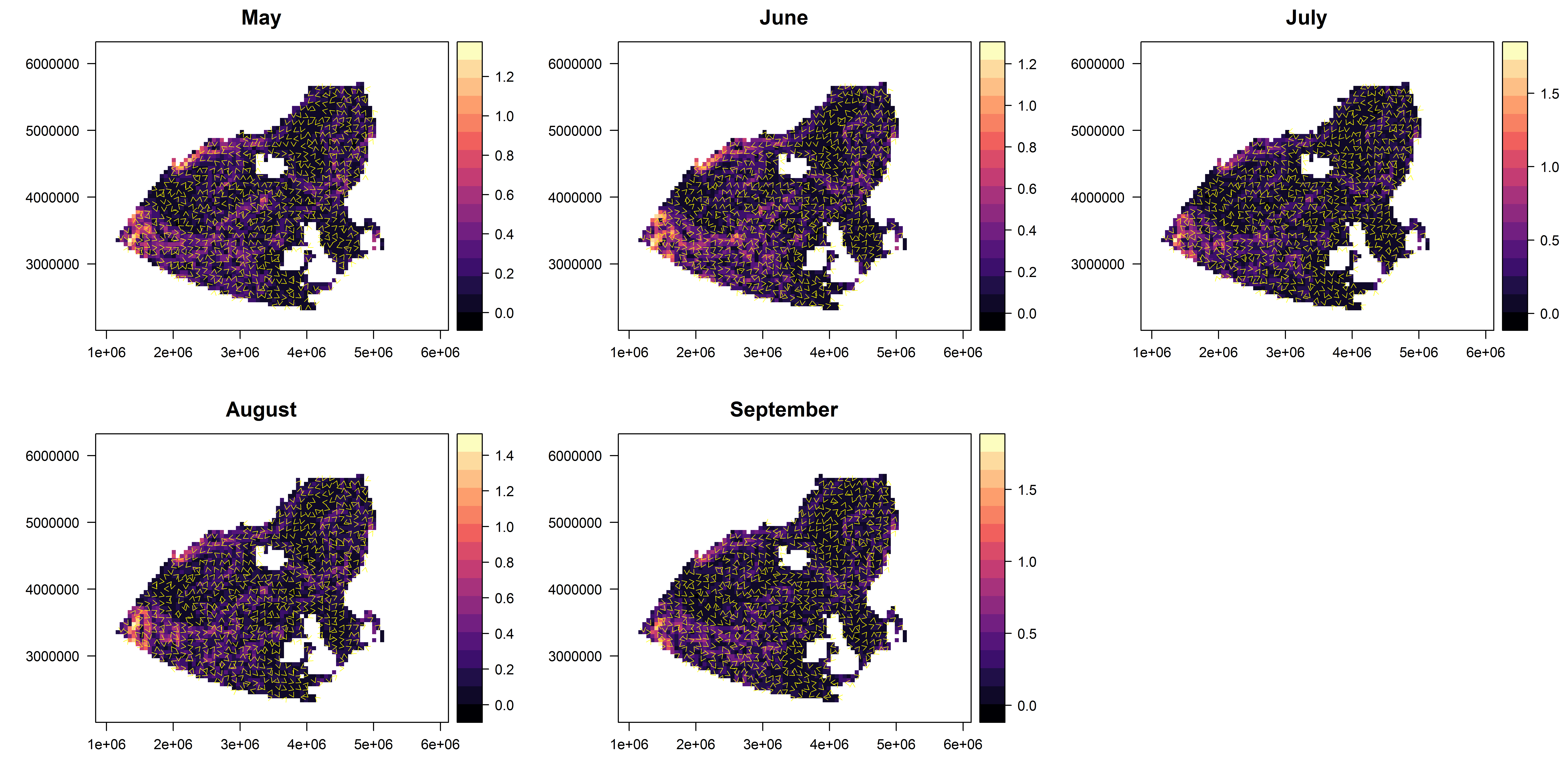


Figure 12. Current velocity (km hour^-1^) for May (top left) through to September (bottom middle). Velocities are calculated as means over the focal month and over 2012 to 2018. Arrows indicate current direction, calculated from the zonal and meridional components.

Photoperiod

For each month photoperiod at varying latitudes was calculated using the daylength() function in R package geosphere (Hijmans 2019a). Values correspond to the 15^th^ day of each month. These data were then used to create rasters (Fig. 13) of photoperiod (as a proportion of 24 hours) in each month for use in the IBM.


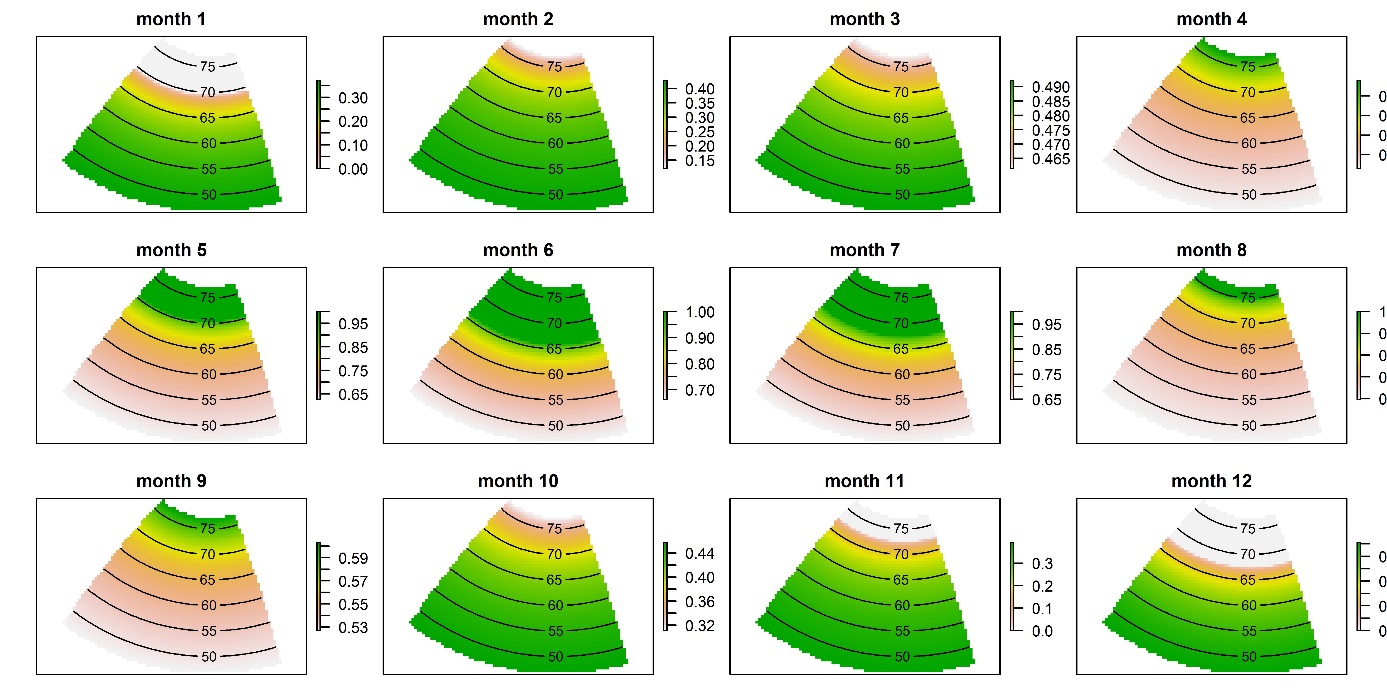


Figure 13. Photoperiod (as a proportion of 24 hours) for each month. Contours show latitude.

# Conceptual model evaluation

**This TRACE element provides supporting information on**: The simplifying assumptions underlying a model’s design, both with regard to empirical knowledge and general, basic principles. This critical evaluation allows model users to understand that model design was not ad hoc but based on carefully scrutinized considerations.

Summary**:**

**The conceptual model is represented by the flowcharts in Figs. 2 and 4. More information on the model’s simplifying assumptions can be found in sections 2 and 3. In this section we give the rationale for 1) the use of food availability and temperature as forcing variables; 2) the spatial structure of the model; and 3) the omission of temperature dependence in the egg development sub-model.**

## Choice of food availability and temperature as model inputs

We chose to use food availability and temperature as forcing variables in our model because they are known to be related to the population dynamics of many fish species, such as egg production and recruitment (Winters 1976, Marshall et al. 1998, Castonguay et al. 2008, Friedland et al. 2008, Jansen 2016). Such relationships are at least in part underpinned by the effects of food and temperature on the rates at which individuals acquire energy, then expend it on vital processes such as maintenance (metabolic rate), growth and reproduction (Giguère et al. 1988, Miller et al. 1992, Yamada et al. 1998, Gillooly et al. 2001, Mendiola et al. 2007). We use generic and established principles of behavioural and physiological ecology to model these processes explicitly (Brown et al. 2004, Sibly et al. 2013).

## Stock spatial distribution

In our model the adult feeding distribution is a fully emergent feature, but the distribution at other times of year, and for different life stages, is to some extent imposed. The nursery area is an approximation of the area of highest juvenile densities within our extent, shown in Jansen et al. (2014). It is also delineated by the 200m isobath off the European shelf, where the author notes most juveniles are found. In Boyd et al. (2018) we defined the spawning area as the “enhanced area” for sampling of western NEA mackerel eggs in MEGS, which is thought to cover the core area of egg production from 2010 (ICES, 2014) (though we cut this off at the IBM’s southern boundary). Within these limits individuals will only move to, and spawn on, patches in which 10°C < SST < 12°C, i.e. their preferred spawning temperature (Sette 1943). In the current formulation, we changed the spawning area to comprise patches on the shelf edge to the west of the Bristish Isles (see section 2). We changed this aspect of the model because habitat suitability modelling has shown that bottom depth is one of the most important determinants of the spawning distribution (Brunel et al. 2017). In the model spawning occurs in five batches (for simplicity), and over 60 days (Dawson and Lockwood, 1986; Eltink, 1987; Watson et al., 1992) in March and April. This covers the peak spawning periods in 2010 and 2013 as shown by egg production curves from the triennial mackerel egg survey (MEGS) (ICES, 2017). See section 2 for details of the adult summer feeding distribution. The overwintering area is assumed to correspond with ICES divisions VIa, to the west of Scotland, and IVa, in the northern North Sea. Tagging and recapture studies indicate that, within our model extent, this is where most adults are found over winter (Uriarte et al., 2001).

## Omission of temperature-dependent egg mortality and development

*S. scombrus* egg development time decreases with temperature (Worley 1933, Mendiola et al. 2006). Initially we included this in the model but recruitment, which is partly determined by egg survival, became overly sensitive to temperature (i.e. recruitment increased drastically with temperature which the literature does not support). Mendiola et al. (2006) show that, as egg development time decreases with temperature, background mortality increases. Hence, cumulative mortality rate does not vary appreciably with temperature (Fig. 14), as indicated by a non-significant slope when regressing cumulative mortality on temperature (p > 0.05). The exception is at 8.6 °C, although in our model we include the constraint that mackerel will not spawn at such low temperatures. In other words, M_back_ is expected to be roughly constant over the temperature at which individuals will spawn in the model because mortality and the rate at which eggs develop cancel each other out. To reflect this, we simply set a constant egg development time and M_back_ (at rate M_e_), so that cumulative background egg mortality rate (egg development period^-1^) does not change. This means that the mortality inflicted on eggs differs only in terms of how many are eaten by other explicitly modelled mackerel, which in turn depends on the number of predators and temperature.


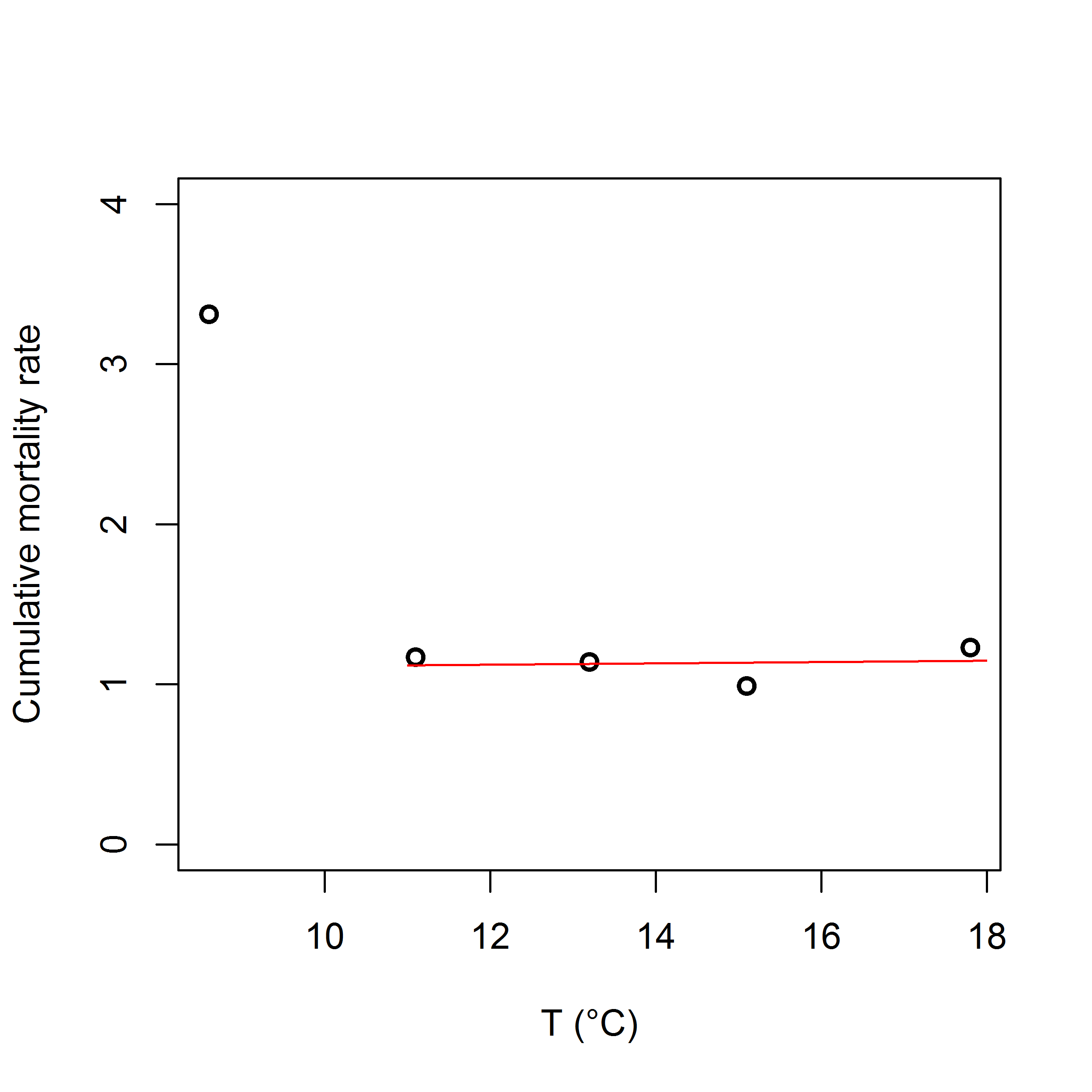


Figure 14. Data from Mendiola et al. (2006) on the cumulative mortality rate (egg development duration^-1^) of *S. scombrus* eggs over the temperature range 8.6 to 18 °C.

# Implementation verification

**This TRACE element provides supporting information on**: (1) whether the computer code implementing the model has been thoroughly tested for programming errors, (2) whether the implemented model performs as indicated by the model description, and (3) how the software has been designed and documented to provide necessary usability tools (interfaces, automation of experiments, etc.) and to facilitate future installation, modification, and maintenance.

Summary:

**To ensure the model works according to its specification in the ODD description, we performed a series of checks. These checks included syntax checking, visual testing through the NetLogo interface, the use of print statements and spot tests with agent and patch monitors, stress tests with extreme parameters, tracking some variables of a cohort through a simulation, and an independent code review.**

We tested the model thoroughly to ensure it behaves as expected. Initial testing was focused on the energy budget. We tracked the food and temperature history of individuals in the model, which allowed us to cross check the energy-budget sub-models with independent implementations in R. We also used print statements (to display the values of key variables) and spot checks with the agent and patch monitors to manually check whether the energy-budget equations were producing the correct outputs. The IBM code has been reviewed by three authors of this paper. The first version of our IBM Boyd et al. (2018) was independently checked by a member of the IBM research group at the University of Reading, Vicky Boult.

## How to install, use and update the model

The model is implemented in NetLogo 5.3.1, a free software platform. The NetLogo code can be found at https://github.com/robboyd/SEASIM-NEAM/tree/master. The model inputs are freely-available and we show how to process them in the supplementary R Markdown documents. R Markdown documents are also provided showing how to run the model through R, and how to run the ABC. If you would like the inputs that we have processed, you will need to provide us somewhere to upload the data (500 GB) online. Users can use the “BehaviourSpace” tool in NetLogo to design new experiments. The developers of NetLogo provide transition guides so the model will be usable in later versions of NetLogo. Users who wish to modify the model structurally will need knowledge of NetLogo.

### User options and model interface

Our IBM has a user-friendly graphical user interface (GUI, Fig. 15). The GUI consists of 1) a visual representation of the simulation; 2) buttons (grey rectangles) that can be used to interact with the IBM; 3) “sliders” (green sliding scale bars) that can be used to specify the value of parameters or variables; 4) “choosers” (green drop down menus) which are used to modify the model settings; 5) dynamic plots (brown rectangles with plots); and 6) monitors (brown rectangles with numeric values) which indicate the current value of a particular variable. In the following we outline what each element of the GUI does or shows.

***Buttons***

- “setup” initializes the model (pre-spin-up).
- “spin-up”: spins up the model for a number of years (see section for details)
- “go” calls the necessary selection of sub-models each time step. Note the arrows which indicate it is a “forever” button, i.e. it runs on a loop for until manually stopped.
- “go-ABC” is identical to “go” but for the appropriate number of time-steps for calibration.
- “go-forecast” is identical to “go” but runs the IBM out to 2050.
- “profiler” runs the IBM for one year and reports diagnostics (e.g. execution speed).

***Sliders***

- “c”,”Me” and ”h” represent the parameters strength of the density dependence, early mortality and the half saturation constant (Table 4). These parameters have been calibrated in the past and the sliders offer an easy way to quickly modify their values and check how the population responds.
- “sim_n” is used as an index to distinguish between simulations when the user wants to conduct multiple runs for an experiment.

***Choosers***

- “feeding_strategy” determines the model used to describe adult movement during the feeding period. Options are: 1) Ideal Free Distribution (“IFD”); 2) Gradient Area Search (“GAS”); or “random”.
- “spawning_strategy” determines the location of the spawning area. Options are: 1) “MEGS”, i.e. the core area of sampling for mackerel eggs in the MEGS survey; or 2) “shelf_edge” in which spawning is constrained to the shelf edge (delineated by depth).
- “Recruitment” determines how recruitment is modelled in the IBM. Options are: 1) “Emergent” in which recruitment emerges from egg production and the survival of young; and 2) “Ricker” in which the early life stages are not modelled explicitly and recruitment is given as a function of SSB and SST on the spawning grounds.
- “n_multiplier” is the number of SIs as a multiple of 1000. Default is 1.
- “high_res” determines the spatial and temporal resolutions of the model. Options are 1) “true” which gives a spatial resolution of 30x30km and a time-step of one day; and 2) “false” which gives a spatial resolution of 60x60km and a time-step of five days. Note that sub-models introduced from 2.0.0 onwards are not set up to work in high resolution.
- “start_year” determines the year in which the model spin-up is to start, i.e. ten years before the actual simulations start. When using RS inputs users cannot change the start year; it is fixed at 1995 (meaning that after the ten year spin up the model starts in 2005).
- “constant_rec?” determines whether or not recruitment inputs in the spin-up period are on a loop, or whether inter-annual variability is incorporated.
- “scale_ssb” is used to adjust SSB at spawning time each year. Options are: 1) “false”, in which SSB is not scaled; 2) “constant”, in which SSB is held constant each year; and 3) “decreasing”, in which SSB decreases at a rate of 5% of its value in 2005 per year.
- “close_a4” determines whether or not ICES division 4a is closed to mackerel fishing. Options are: 1) 0, in which fishing mortality is not spatially-explicit; 2) 1 in which 4a is closed for half of the year; and 3) 2 in which the area is closed permanently.
- “redistribute_F” determines whether or not, when ICES division 4a is closed, fishing mortality that would have taken place inside is relocated elsewhere. 0 = true, 1 = false.
- “match_closure_F” Boolean variable (0 = true, 1 = false). If true, lows total F such that it is equal to the rate that would result from the closure of 4a.
- “future_annual_F” determines the rate of fishing mortality in the forecast period (2019 onwards). Options are: 1) 0, i.e. unfished; 2) “F_MSY (0.23); and 3) “F_lim” (0.46).
- “enviro_inputs” determine the source of the SST and chlorophyll input data. Options are: 1) “RS”, i.e. remote-sensing; and 2) “ESM”, i.e. GFDL-ESM-2M earth system model. If RS is chosen, then the start year is fixed at 2005.
- “RCP” determines which RCP scenario the model inputs correspond to if using GFDL-ESM-2M.
- “export_distribution” is a Boolean variable which determines whether or not .asc files of the stock’s distribution should be exported. 0 = true and 1 = false.
- “force_spin_up_rec” determines how recruitment is modelled in the spin up period. If true, then the 2019 stock assessment recruitment is used (and recruits enter the model as in the Ricker model) or, if constant_rec is true, then recruitment is constant at the level of 2004. If force_spin_up_rec if false, then recruitment emerges as in the simulations. The default is true because this helps the model to settle down in the spin up and gives a reasonable approximation of stock size in the first year of the simulation (i.e. at the end of the spin up).

***Plots and monitors***

Plots and monitors are labelled on Fig 15.


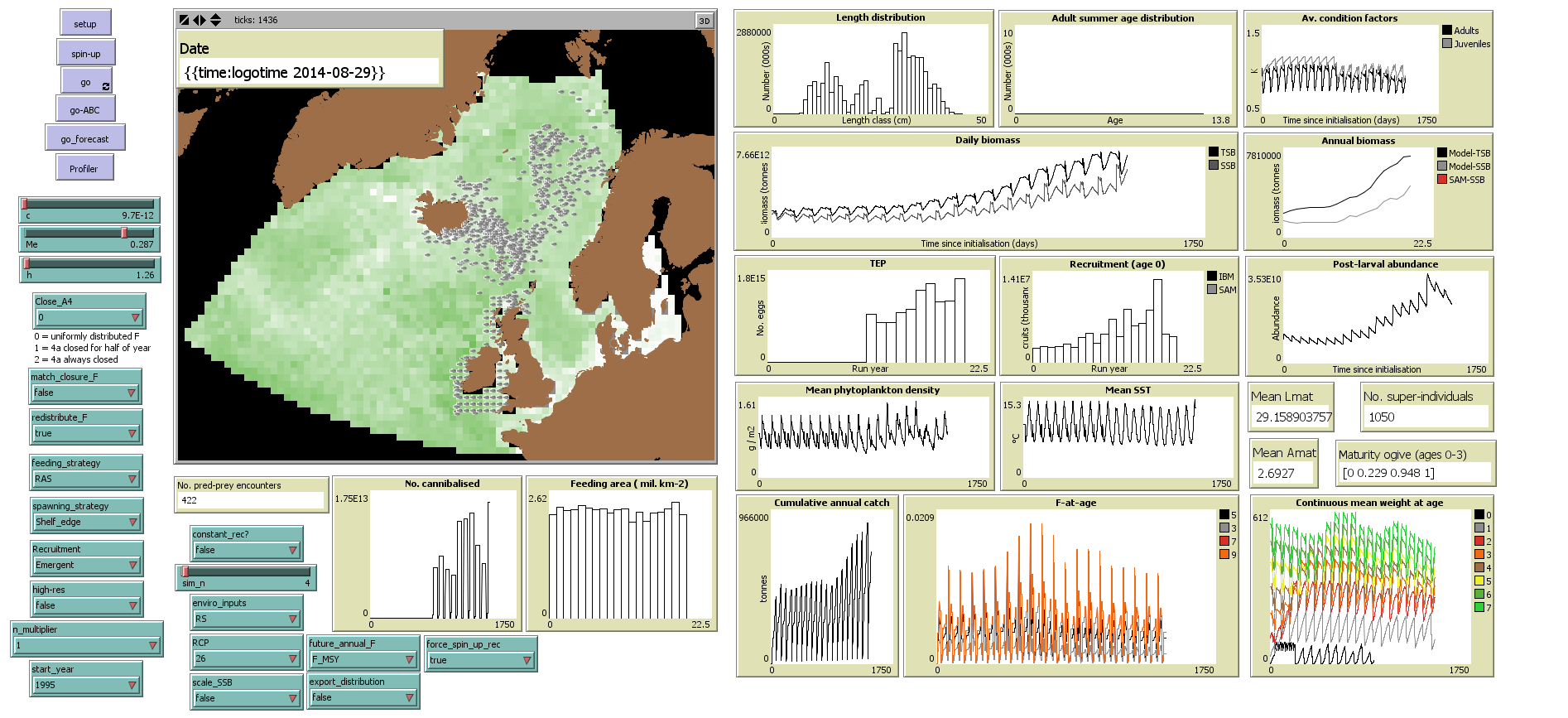


Figure 15. IBM interface. See text for full details. Lmat and Amat are length and age at maturity, respectively. TEP is total egg production.

## Summary of changes to the IBM between versions

Since it was first published we have made structural changes to the IBM. To enable a complete understanding of how the IBM has changed over time, we provide a log of major modifications and when they were made. We refer to versions 1.0.0, 2.0.0 and 3.0.0, denoting the model as presented in Boyd et al. (2018), Boyd et al. (2020) and Boyd et al. (submitted, as here), respectively. A summary of changes made between versions is provided in Table 8.

### Data used for calibration

In 1.0.0 we used data on SSB, recruitment and egg production to calibrate the IBM (See section 3). These data are relative survey indices and give insight into trends in the population dynamics. However, matching these trends does not guarantee that absolute stock size is predicted well.

In 2.0.0 we focused on the mackerel summer feeding distribution. Because intraspecific competition for food is a key driver of the summer distribution, we needed to ensure the IBM predicted absolute stock size well. To do this we fitted the IBM to estimates of SSB from the stock assessment, and weight-at-age in the summer. The rationale here was that if the IBM could match these data simultaneously, then competition for food at a given stock size, as reflected in the weights of the fish, was reasonable.

In 3.0.0 we also used estimates of SSB from the stock assessment and data on weight-at-age for calibration. However, we substituted data on weight-at-age in summer for weight-at-age at spawning time because a longer time-series became available. It is also important to note that the stock assessment is updated each year so the SSB estimates to which the IBM was fitted differ from 2.0.0.

### Spatial distribution and movement models

Key changes to the movement models and spatial distribution between versions are: 1) movement from a fixed to emergent feeding area, based on a mechanistic movement model; and 2) the location of the spawning area.

In 1.0.0 we fixed spawning, feeding, overwintering and nursery areas from information in the literature (see section). Juveniles are constrained to the nursery areas until maturing as adults, at which point they begin to migrate between spawning, feeding and overwintering areas on set dates (Fig. 5). While not migrating individuals move randomly within the designated area. The spawning area comprises the core sampling area in the mackerel egg survey, i.e. the areas of highest expected spawning activity.

From 2.0.0 onwards we made changes to the spatial distribution of the population. First, we removed boundaries to the adult feeding area. Instead, after migrating to the entrance to the feeding area in the Norwegian Sea, they begin to actively move in search of the most profitable patches on which to feed (see 2 section for details). As a result, the spatial distribution over summer months (end of spawning through September) is a fully emergent feature of the IBM. From 2.0.0 onwards we also changed the location of the spawning area. A recent paper showed that mackerel spawning is most intense on the continental shelf edge to the west of the British Isles (Brunel et al. 2017). To reflect this, we restricted spawning to patches on the shelf edge (-500 < depth < -50m).

### Model inputs

In 1.0.0 and 2.0.0 model forcing data (phytoplankton biomass, SST and F) are input in the same way. From 3.0.0 we introduce two changes. First, the user is given the option to substitute satellite-derived estimates of phytoplankton biomass and SST for predictions from an ESM GFDL-ESM-2M. Switching to the ESM inputs allows the model to be run forward in time. Second, we introduce intra-annual variation in F by setting the monthly fraction of annual F proportional to the monthly mean historical fraction of the NEAM catch taken in each month. Prior to 3.0.0 annual F was applied uniformly each time-step within a year.

### Model resolution and extent

From 2.0.0 we make modifications to the spatial and temporal resolutions and extents of the IBM. In 1.0.0 the temporal extent spans from January 1^st^ 2007 to December 31^st^ 2015, and the spatial extent spans from -20 to 10°E, and 47 to 75° N. In 2.0.0 we focus on the summer feeding distribution of NEAM which has recently expanded outside of the area covered by 1.0.0. For this reason we extended the IBM to span -45 to 20°E, and 47 to 77° N. We also extended the temporal extent of the IBM in 2.0.0 so that simulations begin in 2005 as opposed to 2007. In 3.0.0 we extended this even further so that the simulations begin in 2005 and end in 2050. Expanding the spatial and temporal extents of the IBM caused execution speed to slow. For this reason from 2.0.0 onwards we reduced the model time-step from one to five days.

### Initialisation

From 1.0.0 to 3.0.0 we improved the way in which the model is initialized. In 1.0.0 a pre-spin-up population size and structure was approximated from estimates of SSB and TSB from the stock assessment, as well as age and length distributions from catch samples. This population is then spun-up for 20 years before a simulation begins. Subtracting the spin-up period of twenty years from the start of the actual simulation in 2007 gives a start year for the spin-up of 1987. Ideally, we would include realistic inter-annual forcing in the spin-up period, i.e. in 1987 SST and phytoplankton biomass would correspond to that same year, and would differ to that in 1988 etc. This was not possible, however, because the satellite remote-sensing inputs are only available as far back as 2003. For this reason we decided to spin-up the population using input data from the first year of the actual simulation on an annual loop.

In 3.0.0 and 4.0.0 we were able to substantially improve the spin-up procedure. We obtained historical estimates of numbers-at-age from the ICES WGWIDE working group, which allowed us to generate a realistic pre-spin-up population. Moreover, in 3.0.0 we substituted the RS inputs for those from an ESM, which are available much further back in time. This meant in 3.0.0 we were not forced to use input data on a loop in the spin up, but rather could include realistic inter-annual variation. In 4.0.0, RS data is still input on an annual loop in the spin-up period.

Table 8. Summary of changes made to the IBM between versions. Full details of the changes and the rationale behind them can be found in the text. 1.0.0 refers to Boyd et al. (2018), 2.0.0 to Boyd et al. (2020) and 3.0.0 to Boyd et al. (submitted).

| Version | Spatial resolution | Temporal resolution | Spatial extent | Temporal extent | Environmental inputs | Source of environmental inputs | Fishing mortality | Initialisation |  |  |
| --- | --- | --- | --- | --- | --- | --- | --- | --- | --- | --- |
| 1.0.0 | 30 x 30km | 1 day | -30 to 10 East, 47 to 75 North | 2007 to 2015 | Chlorophyll, SST, bathymetry | RS | Varies annually | Calculated from SSB and TSB, 20 year spin-up |  |  |
| 2.0.0 | 60 x 60km | 5 days | -45 to 20 East, 47 to 77 North | 2005 to 2015 | Chlorophyll, SST, bathymetry | RS | Varies annually | Calculated from 1.0.0, 10 year spin-up |  |  |
| 3.0.0 | 60 x 60 km | 5 days | -45 to 20 East, 47 to 77 North | 2001 to 2050 | Chlorophyll, SST, bathmetry, horizontal currents, photoperiod | ESM and RS | Varies monthly | Numbers at age from stock assessment, ten year spin-up |  |  |

# Model output verification

**This TRACE element provides supporting information on**: (1) how well model output matches observations and (2) how much calibration and effects of environmental drivers were involved in obtaining good fits of model output and data.

Summary**:**

**In this section we present model fits to data on SSB and weight-at-age obtained using approximate Bayesian Computation (see section 3 and supplementary R Markdown document for details).**

The IBM was fitted to estimates of SSB from the 2019 stock assessment and data on weight-at-age at spawning time using ABC. The data were taken from stockassessment.org (accessed February 2020). Predicted and observed SSB and weight-at-age are shown in Figs 16 and 17, respectively.


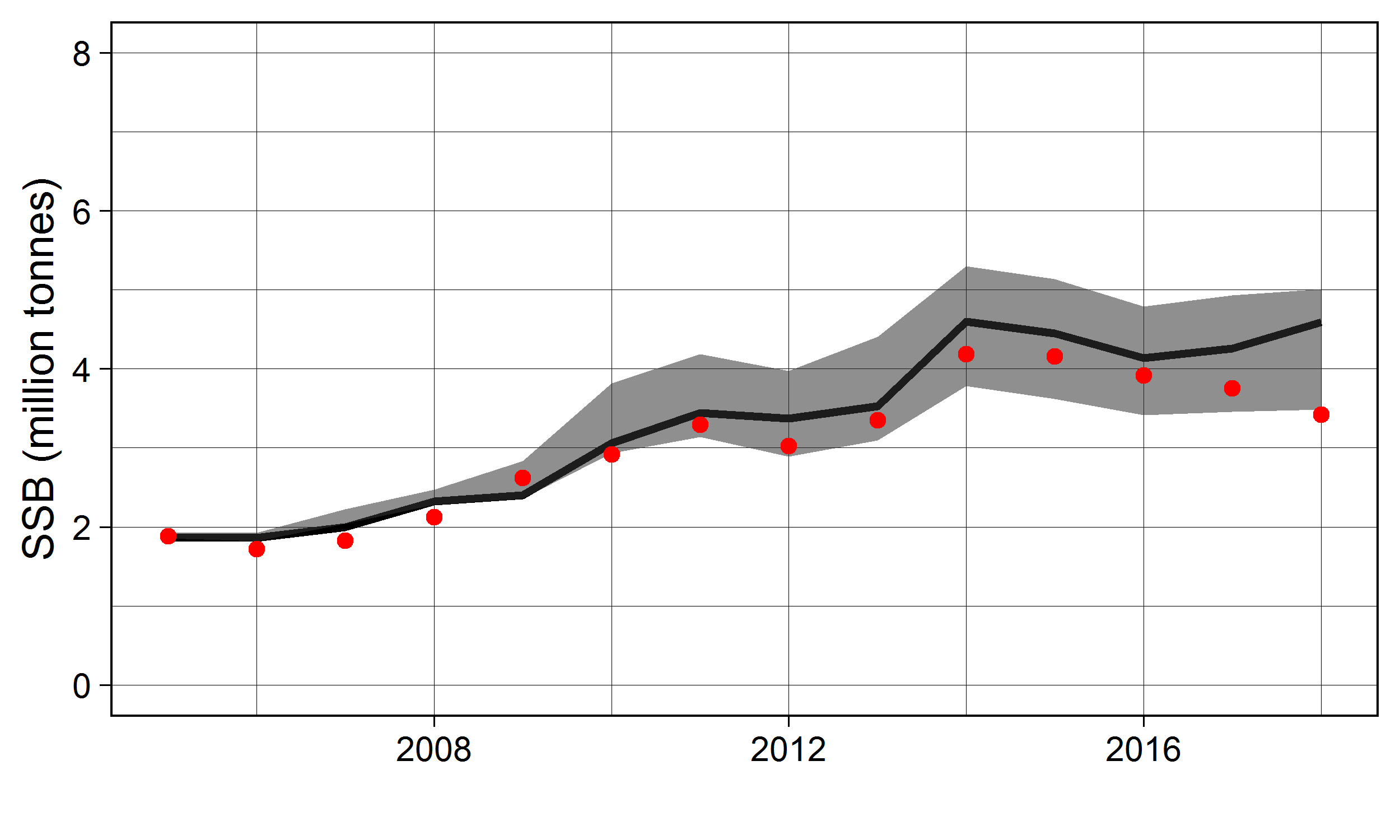


Figure 16. Comparison of predicted (black line is the best fitting simulation) and observed (stock assessment estimate) SSB (red points). The shaded region delimits the 95% credible intervals as estimated by ABC, and represents uncertainty in the calibrated parameters.


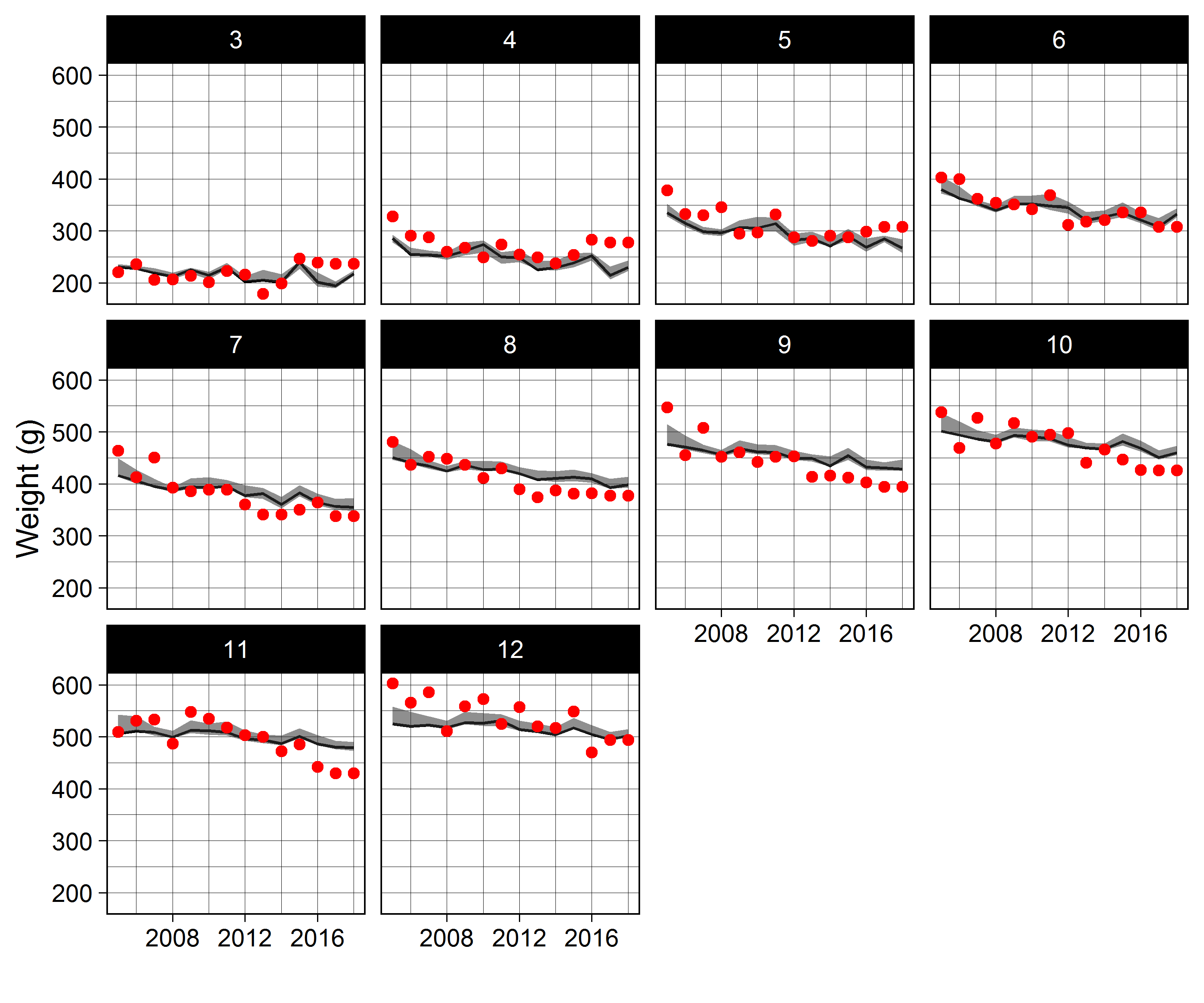


Figure 17. Comparison of the IBM’s predictions of weight at age (lines) and the data (points). The shaded region delimits the 95% credible interval as estimated by ABC, and represents uncertainty in the calibrated parameters.

# Model analysis

**This TRACE element provides supporting information on**: (1) how sensitive model output is to changes in model parameters (sensitivity analysis), and (2) how well the emergence of model output has been understood.

Summary:

**In this section we first provide the results of a local sensitivity analysis. This is presented as the ratio the change in various population outputs to 10% change in the parameter values and was conducted in** Boyd et al. (2020)**. We then look at sensitivity of model outputs to changes in the number of super-individuals, and discuss the results.**

## Local Sensitivity analysis

The relative sensitivities of SSB, recruitment and total egg production (TEP) to each parameter were evaluated using the ratio of % change in the outputs to 10% change in the parameter (Table 9). Changes in outputs are evaluated after one year of simulation. The changes in outputs were calculated as the mean of those that resulted from an increase and decrease in the parameter, and over five simulations for each. Parameters were tested independently. As expected the model is most sensitive to the parameters that determine survival in the egg and larval stages, when the overwhelming majority of mackerel die. The most important parameter is background early mortality rate M_e_, followed by the Gompertz growth rate constant k_1_. These two parameters determine cumulative survival of eggs and larvae because individuals are only susceptible to the high value of M_e_ until reaching a size threshold, meaning the duration of susceptibility depends on k_1_. The model is also sensitive to the activation energy E_a_, which determines its sensitivity to temperature. This is also expected as it affects the values of several of the energy budget parameters (k, k_1_, C_max_, A_0_, S_0_). It is important to note here that the model is stochastic, hence some variability among simulations is expected even without changing parameter values.

Table 9. Mean relative sensitivities of SSB, recruitment and egg production to ± 10% changes in the value of the model’s parameters with ± one standard deviation. While one parameter was tested all others were kept at their value in table 4. Relative sensitivity was calculated as the ratio of the % change in mean output variables to % changes in the parameter, where the changes in output are averaged over those that resulted from an increase and decrease of the parameter. Values represent means over five simulation ± one standard deviation.

|  | Relative sensitivity of output variables | |  |
| --- | --- | --- | --- |
| Parameter | **SSB** | **Recruitment** | **TEP** |
| a_AMR_ | 0.2 ± 0.06 | 0.76 ± 0.4 | 0.29 ± 0.2 |
| A_e_ | 0.38 ± 0.09 | 0.78 ± 0.48 | 0.38 ± 0.23 |
| a_f_ | 0.27 ± 0.07 | 1.41 ± 0.77 | 0.88 ± 0.41 |
| A_r_ | 0.14 ± 0.08 | 0.65 ± 0.33 | 0.42 ± 0.34 |
| a_SMR_ | 0.12 ± 0.09 | 0.75 ± 0.4 | 0.33 ± 0.32 |
| a_v_ | 0.1 ± 0.09 | 1 ± 0.4 | 0.34 ± 0.2 |
| a_w_ | 0.11 ± 0.06 | 1.13 ± 0.37 | 0.38 ± 0.25 |
| b_f_ | 1.12 ± 0.04 | 4.49 ± 0.86 | 4.61 ± 1.33 |
| b_w_ | 0.08 ± 0.03 | 0.73 ± 0.4 | 0.35 ± 0.23 |
| c | 0.22 ± 0.18 | 0.58 ± 0.41 | 0.35 ± 0.21 |
| C_max_ | 0.38 ± 0.1 | 0.5 ± 0.27 | 0.65 ± 0.33 |
| E_a_ | 0.15 ± 0.07 | 4.52 ± 0.56 | 0.48 ± 0.21 |
| E_fl_ | 0.23 ± 0.2 | 0.53 ± 0.18 | 0.35 ± 0.21 |
| E_flesh_ | 0.37 ± 0.13 | 1.21 ± 0.33 | 1.12 ± 0.21 |
| E_fs_ | 0.11 ± 0.07 | 0.76 ± 0.89 | 0.29 ± 0.25 |
| E_lipid_ | 0.15 ± 0.03 | 0.92 ± 0.31 | 0.46 ± 0.14 |
| E_p_ | 0.67 ± 0.05 | 1.39 ± 0.67 | 1.12 ± 0.36 |
| h | 0.21 ± 0.13 | 0.67 ± 0.4 | 0.43 ± 0.22 |
| k | 0.35 ± 0.13 | 1.05 ± 0.23 | 1.19 ± 0.28 |
| K_1_ | 0.06 ± 0.03 | 13.2 ± 0.43 | 0.42 ± 0.3 |
| L_1_ | 0.1 ± 0.03 | 1.61 ± 0.5 | 0.31 ± 0.15 |
| L_∞_ | 0.44 ± 0.03 | 0.4 ± 0.24 | 0.61 ± 0.36 |
| L_mat_ | 1.07 ± 0.12 | 1.56 ± 0.33 | 0.9 ± 0.33 |
| M_0_ | 0.59 ± 0.17 | 1.91 ± 0.54 | 2.35 ± 0.71 |
| M_a_ | 0.25 ± 0.31 | 0.88 ± 0.39 | 0.46 ± 0.1 |
| M_e_ | 0.06 ± 0.04 | 13.79 ± 1.8 | 0.39 ± 0.18 |

## Sensitivity to number of super-individuals

In this section we test the sensitivities of summer SSB (August 1^st^) and recruitment to changes in the number of super-individuals n. This analysis was formally conducted for Boyd et al. (2018), but the results hold in later versions of the IBM. From Fig. 18 it is clear that, despite some stochasticity in the model, SSB and recruitment are not appreciably affected by the choice of n.


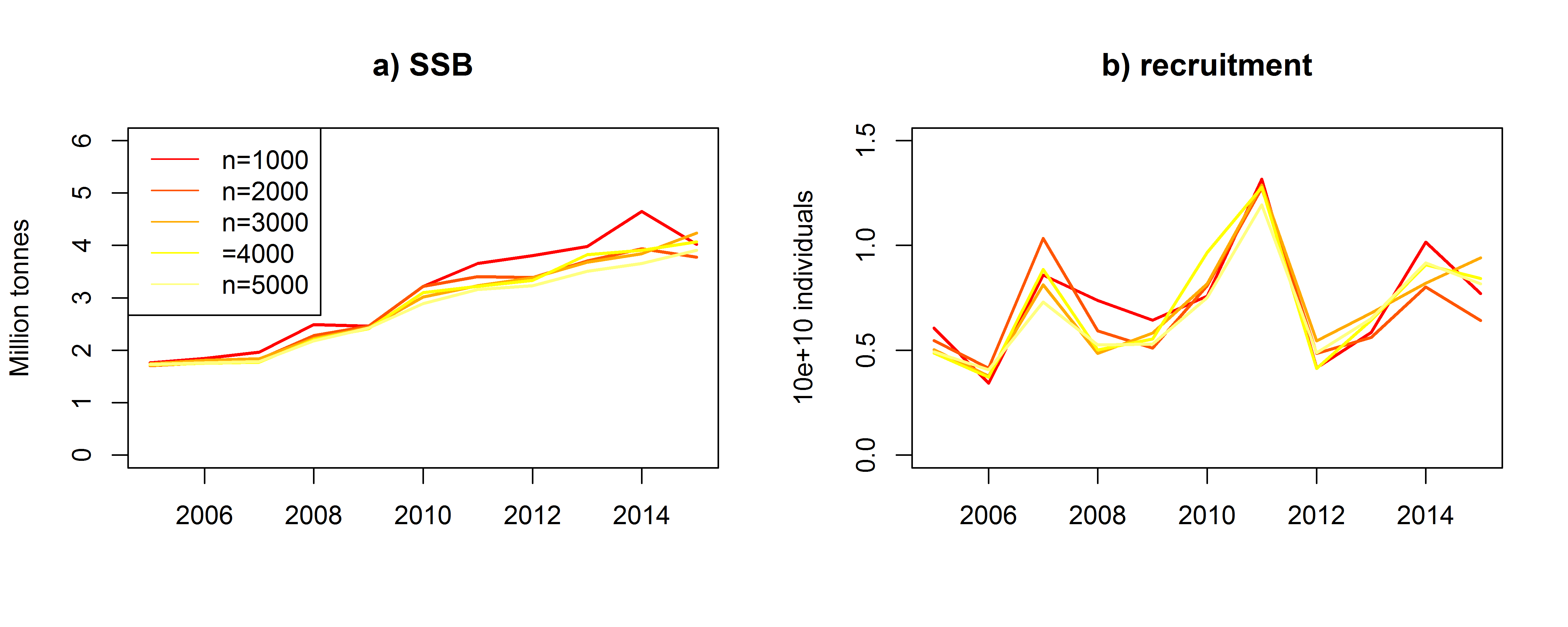


Figure 18. Predicted a) SSB and b) recruitment with different numbers of super-individuals.

## Optimising threshold density for presence

To assess the IBM‘s ability to match data that was not used in the ABC, we compare its predictions of presence/ absence over July/ August to observations from the Internation Ecosystem Survey in the Noridc Seas (IESSNS, Fig. 20). See Nøttestad et al. (2015) for details of these data, which we approximated from Olafsdottir et al. (2018) using Java’s PlotDigitizer (<http://plotdigitizer.sourceforge.net/>). To assess model fits we used two standard statistics for binary data, sensitivity and specificity, i.e. the proportion of presences and absences correctly classified, respectively. As is standard, we first optimised a threshold mackerel density (patch^-1^) above which that patch is classed as a presence, and below which it is classed as an absence. To do this we selected the threshold that minimised the Eclidean distance to point 0,1 (a perfectly classified model) on a plot of sensitivity as a function of 1 – specificity (Fig. 19) (Cantor et al. 1999, Liu et al. 2005). Other skill metrics can be used to decide on a threshold, but we chose the distance to point 0,1 on fig. 19 because it robust to asymmetry in the prevalence of presences and absences in data which is the case here (~82% presences). After optimising this threshold, sensitivty and specificity values of 0.73 and 0.68 were obtained, respectively. Note that we pooled the predictions and data across all (surveyed) years over 2007 to 2015. This gives extra weight to years in which sampling effort was higher, which we consider appropriate.

See Fig. 20 for a comparison of predicted and observed presence/ absence over the summer months. When comparing predictions of a continuous variable (here mackerel density per patch) to presence/ absence data, it is useful to optimise a threshold above which a continuous value is classed as a presence, and below which it is classed as an absence. The effects of varying the threshold value can be assessed by plotting sensitivity as a function of 1 – specificity, i.e. a receiver-operator curve (ROC, Fig. 19), as obtained with each tested threshold value. We optimised the threshold by selecting the value which minimised the Eucliudean distance to point 0, 1 (i.e. a perfectly classified model) on the ROC (Liu et al. 2005). This threshold was 0.003 times the maximum predicted density, and resulted in a specificity of 0.68, and a sensitivity of 0.73. The area under the receiver operator curve (AUC) is 0.75.


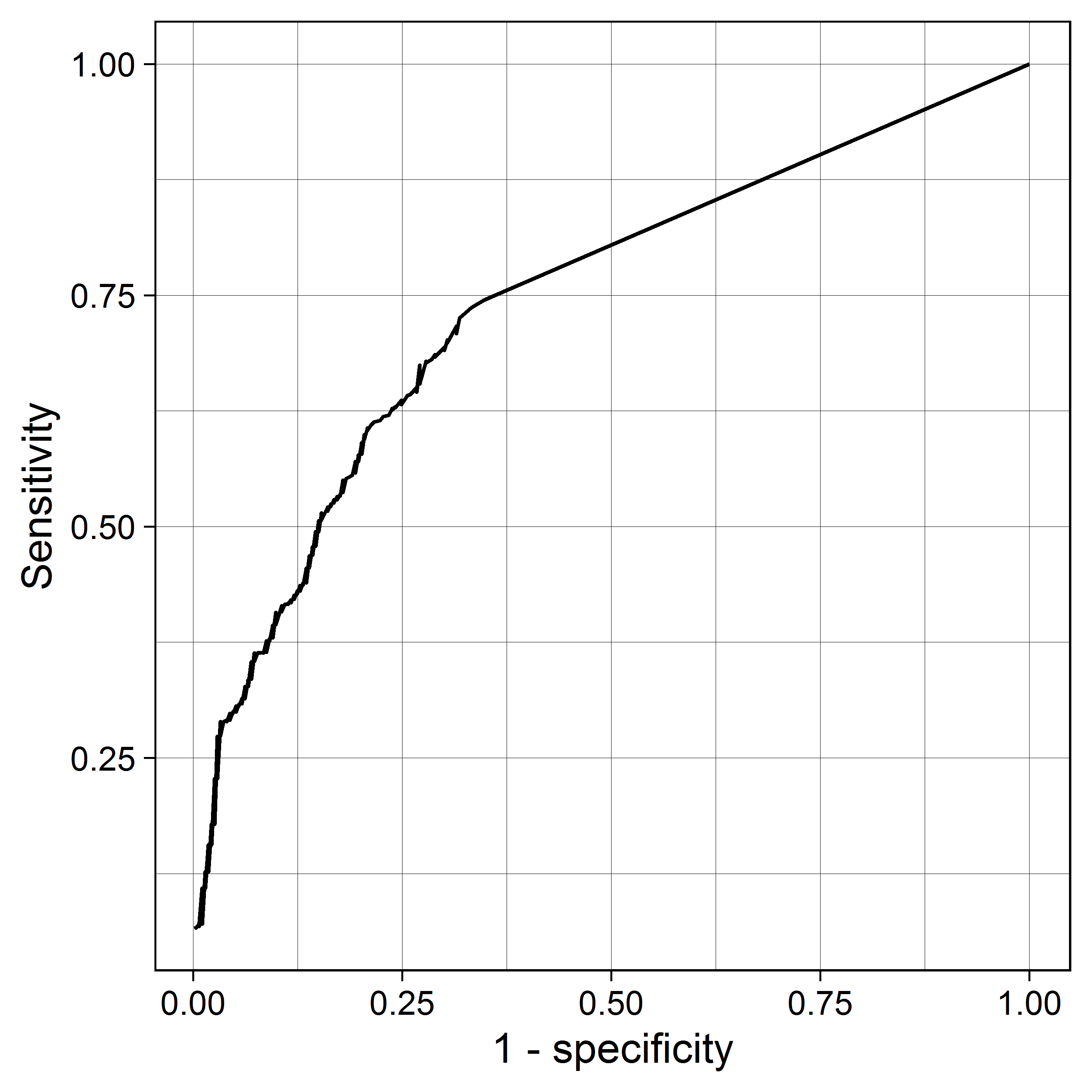


Figure 19. Sensitivity as a function of 1 – specificity (loss of specificity) for the IBM‘s predictions of mackerel presence/ absence in the summer months when compared with the data in Fig. 20. The curve represents varying density thresholds above which a value is classed as a presence, and below which it is classed as an absence, over the range 0 to 30% maximum predicted density.

# Model output corroboration

**This TRACE element provides supporting information on**: How model predictions compare to independent data and patterns that were not used, and preferably not even known, while the model was developed, parameterized, and verified. By documenting model output corroboration, model users learn about evidence which, in addition to model output verification, indicates that the model is structurally realistic so that its predictions can be trusted to some degree.

Summary:

**In this section we validate the IBM by demonstrating its ability to match data on NEAM occurrence in the Nordic seas over July/ August. This data was not used in for calibration.**


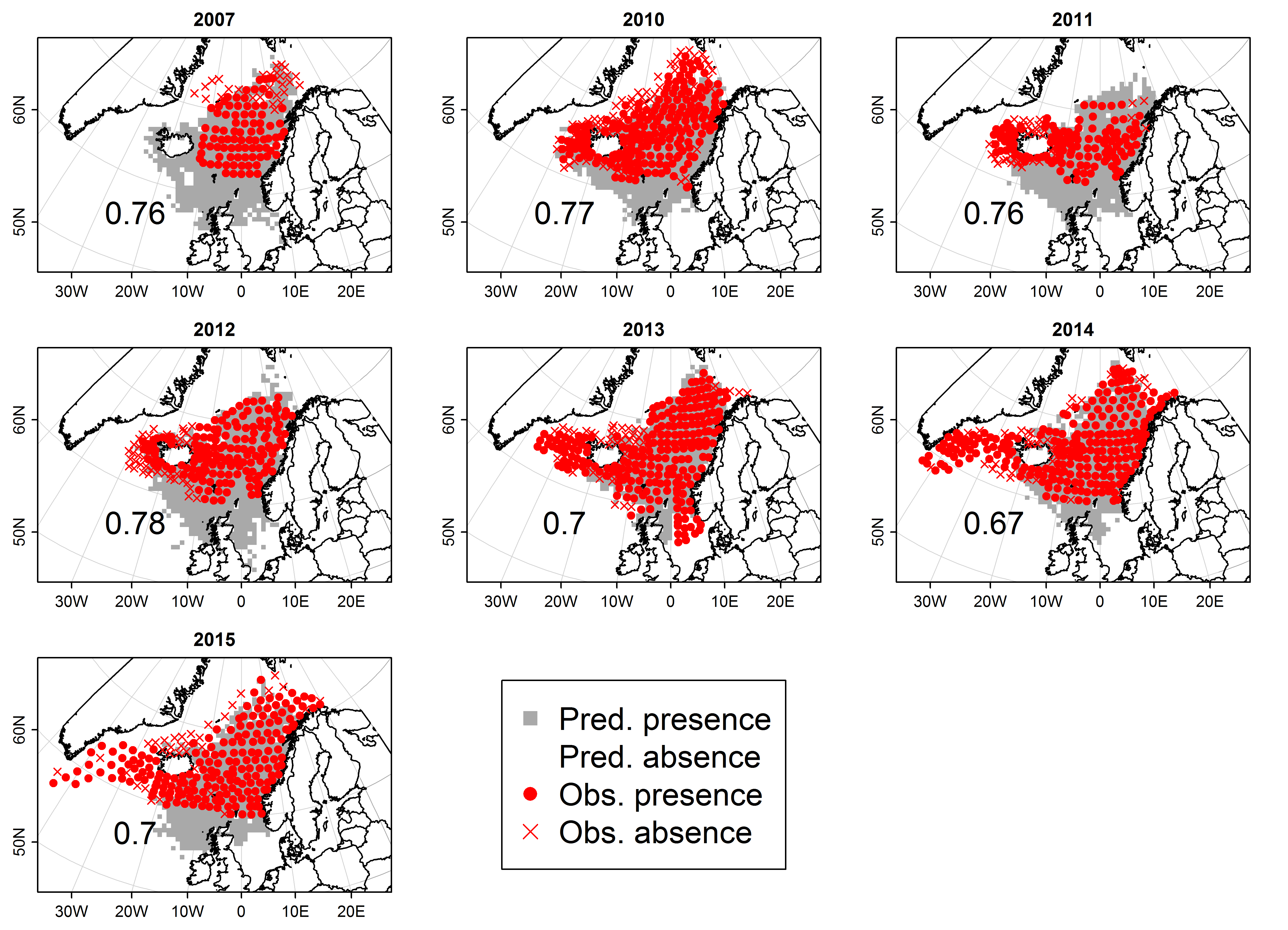


Figure 20. Data from the IESSNS survey (approximated from Olafsdottir et al. (2018)) on presence (obs. presence) and absence (obs. absence) of mackerel in the Nordic seas over July/ August. We also show simulated presence (pred. presence) and absence (pred. absence) as predicted by one simulation using the IFD_dd_ search mechanism. The numbers on each panel represent the proportion of data points in each year for which the model correctly predicted whether or not mackerel was present. Note that these outputs were obtained using remote-sensing environmental inputs as opposed to those from the ESM.

# References

Annis, E. R., E. D. Houde, L. W. Harding, M. E. Mallonee, and M. J. Wilberg. 2011. Calibration of a bioenergetics model linking primary production to Atlantic menhaden Brevoortia tyrannus growth in Chesapeake Bay. Marine Ecology Progress Series 437:253–267.

Bachiller, E., and X. Irigoien. 2013. Marine Science. ICES Journal of Marine Science 70:352–361.

Beddington, J. R. 1975. Mutual interference between parasites or predators and its effect on searching efficiency. The Journal of Animal Ecology 44:331–340.

Bernreuther, M., J.-P. Herrmann, M. A. Peck, and A. Temming. 2013. Growth energetics of juvenile herring, *Clupea harengus*  L.: food conversion efficiency and temperature dependency of metabolic rate. Journal of Applied Ichthyology 29:331–340.

von Bertalanffy, L. 1938. A quantitative theory of organic growth. Human Biology 10:181–213.

Boyd, R., S. Roy, R. Sibly, R. Thorpe, and K. Hyder. 2018. A general approach to incorporating spatial and temporal variation in individual-based models of fish populations with application to Atlantic mackerel. Ecological Modelling 382:9–17.

Boyd, R., R. M. Sibly, K. Hyder, R. B. Thorpe, N. D. Walker, and S. Roy. 2020. Simulating the summer feeding distribution of Northeast Atlantic mackerel with a mechanistic individual-based model. Progress in Oceanography 183:102299.

Brett, J. R., and D. Grove. 1979. Environmental factors and growth. Page Fish Physiology: Bioenergetics and Growth.

Brodziak, J., J. Ianelli, K. Lorenzen, and R. D. Methot. 2011. Estimating Natural Mortality in Stock Assessment Applications. U.S. Dep. Commer., NOAA Tech. Memo. NMFS-F/SPO-119, 38 p.:38.

Brown, J. H., J. F. Gillooly, a P. Allen, V. M. Savage, and G. B. West. 2004. Toward a metabolic theory of ecology. Ecology 85:1771–1789.

Brunel, T., C. J. G. van Damme, M. Samson, and M. Dickey-Collas. 2017. Quantifying the influence of geography and environment on the northeast Atlantic mackerel spawning distribution. Fisheries Oceanography:1–15.

Cantor, S. B., C. C. Sun, G. Tortolero-Luna, R. Richards-Kortum, and M. Follen. 1999. A comparison of C/B ratios from studies using receiver operating characteristic curve analysis. Journal of Clinical Epidemiology 52:885–892.

Castonguay, M., S. Plourde, D. Robert, J. A. Runge, and L. Fortier. 2008. Copepod production drives recruitment in a marine fish. Canadian Journal of Fisheries and Aquatic Sciences 65:1528–1531.

Cotano, U., and P. Alvarez. 2003. Growth of young-of-the-year mackerel in the Bay of Biscay. Journal of Fish Biology 62:1010–1020.

Dawson, W. A., and S. J. Lockwood. 1986. Changes in western mackerel (Scomber scombrus) spawning stock composition during the spawning season. Journal of the marine biological association of the United Kingdom:367–383.

DeAngelis, D. L., R. a. Goldstein, and R. V. O’Neill. 1975. A Model for Tropic Interaction. Ecology 56:881–892.

Dickson, K. a, J. M. Donley, C. Sepulveda, and L. Bhoopat. 2002. Effects of temperature on sustained swimming performance and swimming kinematics of the chub mackerel Scomber japonicus. The Journal of experimental biology 205:969–980.

Dunne, J. P., J. G. John, S. Shevliakova, R. J. Stouffer, J. P. Krasting, S. L. Malyshev, P. C. D. Milly, L. T. Sentman, A. J. Adcroft, W. Cooke, K. A. Dunne, S. M. Griffies, R. W. Hallberg, M. J. Harrison, H. Levy, A. T. Wittenberg, P. J. Phillips, and N. Zadeh. 2013. GFDL’s ESM2 global coupled climate-carbon earth system models. Part II: Carbon system formulation and baseline simulation characteristics. Journal of Climate 26:2247–2267.

Elliott, J. M., and W. Davison. 1975. Energy equivalents of oxygen consumption in animal energetics. Oecologia 19:195–201.

Eltink, a. T. G. W. 1987. Changes in age - size distribution and sex ratio during spawning and migration of Western mackerel (Scomber scombrus L.). J. Cons. int. Explor. Mer 44:10–22.

ESR. 2009. OSCAR third degree resolution ocean surface currents. Ver. 1. PO.DAAC, CA, USA, accessed February 2020.

Fernö, A., T. J. Pitcher, W. Melle, and L. Nøttestad. 1998. The challenge of the herring in the Norwegian Sea: making optimal collective spatial decisions. Sarsia 83:149–167.

Friedland, K. D., J. a. Hare, G. B. Wood, L. a. Col, L. J. Buckley, D. G. Mountain, J. Kane, J. Brodziak, R. G. Lough, and C. H. Pilskaln. 2008. Does the fall phytoplankton bloom control recruitment of Georges Bank haddock, Melanogrammus aeglefinus, through parental condition? Canadian Journal of Fisheries and Aquatic Sciences 65:1076–1086.

Fry, F. E. J. 1971. Effect of environmental factors on the physiology of fishes. The Yale journal of biology and medicine 30:152–153.

Giguère, L., B. Coté, and J.-J. St-Pierre. 1988. Metabolic rates scale isometrically in larval fishes. Marine Ecology Progress Series 50:13–19.

Gillooly, J. F., A. Allen, V. Savage, E. Charnov, G. West, and J. Brown. 2006. Response to Clarke and Fraser : effects of temperature. Functional Ecology 20:400–404.

Gillooly, J. F., J. H. Brown, and G. B. West. 2001. Effects of Size and Temperature on Metabolic Rate. Science 293:2248–2252.

Gluyas-Millan, M. G., M. Castonguay, and C. Quinonez-Velazouez. 1998. Growth of juvenile Pacific mackerel, Scomber japonicus in the Gulf of California. Scientia Marina 62:225–231.

Grégoire, F., H. Dionne, C. Lévesque, M. Lamontagne, P. O. Box, and D. Mer. 1992. Fat content of Atlantic mackerel ( Scomber scombrus L .) in 1991 and 1992 Canadian lndustry Report of Fisheries and Aquatic Sciences 220.

Grimm, V., U. Berger, F. Bastiansen, S. Eliassen, V. Ginot, J. Giske, J. Goss-Custard, T. Grand, S. K. Heinz, G. Huse, A. Huth, J. U. Jepsen, C. Jørgensen, W. M. Mooij, B. Müller, G. Pe’er, C. Piou, S. F. Railsback, A. M. Robbins, M. M. Robbins, E. Rossmanith, N. Rüger, E. Strand, S. Souissi, R. a. Stillman, R. Vabø, U. Visser, and D. L. DeAngelis. 2006. A standard protocol for describing individual-based and agent-based models. Ecological Modelling 198:115–126.

Grimm, V., U. Berger, D. L. DeAngelis, J. G. Polhill, J. Giske, and S. F. Railsback. 2010. The ODD protocol: A review and first update. Ecological Modelling 221:2760–2768.

Hatanaka, M., K. Sekino, M. Takahashi, and T. Ichimura. 1957. Growth and food consumption in young mackerel, Pneumatophorus japiconus. Journal of agricultural research VII.

Herrmann, J. P., and E. C. Enders. 2000. Effect of body size on the standard metabolism of horse mackerel. Journal of Fish Biology 57:746–760.

Hijmans, R., J. van Etten, J. Cheng, M. Mattiuzzi, M. Sumner, J. a Greenberg, O. Lamigueiro, A. Bevan, E. Racine, and A. Shortridge. 2016. Package “ raster ”. CRAN -R.2.5-8.

Hijmans, R. J. 2019a. geosphere: Spherical Trigonometry. R package version 1.5-10.

Hijmans, R. J. 2019b. raster: Geographic Data Analysis and Modeling. R package version 2.9-23.

ICES. 2012. 1.7 Acronyms and terminology.

ICES. 2014. Manual for the mackerel and horse mackerel egg surveys (MEGS): sampling at sea. Page Series of ICES Survey Protocols.

ICES. 2017. ICES WGWIDE REPORT 2014 Report of the Report of the Working Group on Widely Distributed Stocks ( WGWIDE ) International Council for the Exploration of the Sea.

ICES. 2019a. Norway special request for revised 2019 advice on mackerel ( Scomber scombrus ) in subareas 1 – 8 and 14 , and in Division 9 . a ( the Northeast Atlantic and adjacent waters ):1–17.

ICES. 2019b. Mackerel (Scomber scombrus) in subareas 1–8 and 14, and in Division 9.a (the Northeast Atlantic and adjacent waters). ICES Advice on fishing opportunities, catch, and effort.

Jansen, T. 2016. First-year survival of North East Atlantic mackerel (Scomber scombrus) from 1998 to 2012 appears to be driven by availability of Calanus, a preferred copepod prey. Fisheries Oceanography.

Jansen, T., A. Campbell, C. Kelly, H. Hátún, and M. R. Payne. 2012. Migration and Fisheries of North East Atlantic Mackerel (Scomber scombrus) in Autumn and Winter. PLoS ONE 7.

Jansen, T., and H. Gislason. 2011. Temperature affects the timing of spawning and migration of North Sea mackerel. Continental Shelf Research 31:64–72.

Jansen, T., K. Kristensen, J. Van Der Kooij, S. Post, A. Campbell, K. R. Utne, P. Carrera, J. A. Jacobsen, A. Gudmundssdottir, B. A. Roel, and E. M. C. Hatfield. 2014. Nursery areas and recruitment variation of Northeast Atlantic mackerel (Scomber scombrus). ICES Journal of Marine Science.

Johnson, P. O. 1977. A review of spawning in the north Atlantic Mackerel, Scomber scombrus L.

Kooijman, S. A. L. M., and J. A. J. Metz. 1983. On the dynamics of chemically stressed populations: The deduction of population consequences from effects on individuals. Hydrobiological Bulletin 17:88–89.

Lambert, T. C. 1985. Gastric emptying time and assimilation efficiency in Atlantic mackerel (Scomber scombrus ). Canadian Journal of Zoology 63:817–820.

Link, J., C. Griswold, E. Methratta, and J. Gurnard. 2006. Documentation for the Energy Modeling and Analysis eXercise (EMAX). Page Northeast Fisheries Science Center Reference Document 06-15.

Liu, C., P. M. Berry, T. P. Dawson, and R. G. Pearson. 2005. Thresholds of Occurrence in the Prediction of Species Distributions 28:385–393.

Lockwood, S. J., J. H. Nichols, and W. A. Dawson. 1981. The estimation of a mackerel (Scomber scombrus L.) spawning stock size by plankton survey. Journal of Plankton Research 3:217–233.

Marshall, C. T., O. S. Kjesbu, N. a Yaragina, P. Solemdal, and Ø. Ulltang. 1998. Is spawner biomass a sensitive measure of the reproductive and recruitment potential of Northeast Arctic cod? Canadian Journal of Fisheries and Aquatic Sciences 55:1766–1783.

Mendiola, D., P. Alvarez, U. Cotano, E. Etxebeste, and a. M. de Murguia. 2006. Effects of temperature on development and mortality of Atlantic mackerel fish eggs. Fisheries Research 80:158–168.

Mendiola, D., P. Alvarez, U. Cotano, and A. Martinez De Murguia. 2007. Early development and growth of the laboratory reared north-east Atlantic mackerel Scomber scombrus L. Journal of Fish Biology 70:911–933.

Miller, T. J., L. B. Crowder, J. a. Rice, and F. P. Binkowski. 1992. Body Size and the Ontogeny of the Functional Response in Fishes. Canadian Journal of Fisheries and Aquatic Sciences 49:805–812.

NASA. 2017. Aqua Moderate Resolution Imaging Spectroradiometer (MODIS) chlor_a ocean color data.

NASA OBPG. 2017. Moderate-resolution Imaging Spectroradiometer (MODIS) Aqua Sea Surface Temperature (daytime) Data; 2014 reprocessing. NASA Goddard Space Flight Center, Ocean Ecology Laboratory, Ocean Biology Processing Group., NASA OB.DAAC, Greenbelt, MD, USA.

NOAA. 2017. NOAA Optimum Interpolation SST Data. https://www.esrl.noaa.gov/psd/cgi-bin/GrADS.pl?dataset=NOAA Optimum Interpolation %28OI%29 SST V2;DB_did=62;file=%2FDatasets%2Fnoaa.oisst.v2%2Fsst.mnmean.nc;variable=sst;DB_vid=1295;DB_tid=59229;units=degC;longstat=Mean;DB_statistic=Mean;stat=;lat-begin=4.

NODC. 2017. Gridded bathymretric chart of the oceans (GEBCO) data, elevation relative to sea level, National oceanographic data centre.

Nøttestad, L., J. Diaz, H. Peña, H. Søiland, G. Huse, and A. Fernö. 2016. Feeding strategy of mackerel in the Norwegian Sea relative to currents, temperature, and prey. ICES Journal of Marine Science 73:1127–1137.

Nøttestad, L., S. Þ. Jo, J. A. Jacobsen, K. R. Utne, J. O. Guðmundur, Ø. Tangen, V. Anthonypillai, S. Aanes, J. H. Vølstad, M. Bernasconi, J. C. Holst, T. Jansen, A. Slotte, H. Debes, L. Smith, and S. Sveinbjo. 2015. Quantifying changes in abundance, biomass and spatial distribution of Northeast Atlantic mackerel in the Nordic seas from 2007 to 2014. ICES Journal of Marine Science 73:359–373.

Ólafsdóttir, A. H., K. R. Utne, J. A. Jacobsen, T. Jansen, G. J. Óskarsson, L. Nøttestad, B. Þ. Elvarsson, C. Broms, and A. Slotte. 2018. Geographical expansion of Northeast Atlantic mackerel (Scomber scombrus) in Nordic Seas from 2007 - 2016 was primarily driven by stock size and constrained by low temperatures. Deep-Sea Research Part II: Topical Studies in Oceanography:0–1.

Pepin, P., J. A. Koslow, and S. Pearre. 1988. Laboratory study of foraging by Atlantic mackerel, Scomber scombrus, on natural zooplankton assemblages. Can J Fish Aquat Sci 45:879–887.

Peters, R. 1983. Ecological implications of body size. Cambridge University Press, New York.

Peterson, W., and S. Ausubel. 1984. Diets and selective feeding by larvae of Atlantic mackerel Scomber scombrus on zooplankton . Marine Ecology Progress Series 17:65–75.

Petitgas, P., A. Uriarte, E. Nogueira, J. Massé, and U. Cotano. 2010. Life-cycle spatial patterns of small pelagic fish in the Northeast Atlantic. ICES Cooperative Research Report 306:40–44.

Pinnegar, J. K. 2014. DAPSTOM - An Integrated Database & Portal for Fish Stomach Records. Page Cefas contract report.

Politikos, D., M. Huret, and P. Petitgas. 2015. A coupled movement and bioenergetics model to explore the spawning migration of anchovy in the Bay of Biscay. Ecological Modelling 313:212–222.

Pullar, J., and A. Webster. 1977. The energy cost of fat and protein deposition in the rat. British journal of nutrition 37:355–363.

Railsback, S. F., and V. Grimm. 2010. Models, Agent-Based Models, and the Modeling Cycle 1.1. Agent-Based and Individual-Based Modeling: A Practical Introduction:3–13.

Sambilay Jr, V. 1990. Interrelationships between swimming speed, caudal fin aspect ratio and body length of fishes.

Scheffer, M., J. M. Baveco, D. L. Deangelis, K. a Rose, and E. H. Vannes. 1995. Super-Individuals A Simple Solution For Modeling Large Populations On An Individual Basis. Ecological Modelling 80:161–170.

Schmidt-Nielsen, K. 2013. Animal physiology: Adaption and environment. Fifth edition. Cambridge University Press.

Sette, O. 1943. Part I. Biology of the atlantic mackerel (Scomber scombrus) of North America. Fishery bulletin 50.

Shin, Y. J., and P. Cury. 2001. Exploring fish community dynamics through size-dependent trophic interactions using a spatialized individual-based model. Aquatic Living Resources 14:65–80.

Sibly, R. M., and P. Calow. 1986. Physiological ecology of animals: an evolutionary approach. Blackwell.

Sibly, R. M., V. Grimm, B. T. Martin, A. S. a Johnston, K. Kulakowska, C. J. Topping, P. Calow, J. Nabe-Nielsen, P. Thorbek, and D. L. Deangelis. 2013. Representing the acquisition and use of energy by individuals in agent-based models of animal populations. Methods in Ecology and Evolution 4:151–161.

Sirnard, P., M. Castonguay, D. D. Arnours, and P. Magnan. 1992. Growth comparison between juvenile Atlantic mackerel from the two pawning groups of the Northwest Atlantic. Canadian Journal of Fisheries and Aquatic Sciences 49.

Steven, G. 1952. Contributions to the biology of the mackerel, Scomber scombrus L. III. Age and growth. Journal of the marine biological association of the United Kingdom 30.

Studholme, a. L., D. B. Packer, P. L. Berrien, D. L. Johnson, C. a. Zetlin, and W. W. Morse. 1999. Atlantic Mackerel, Scomber scombrus, Life history and Habitat Characteristics. NOAA Technical report NMFS-NE-141:44.

Tittensor, D. P., T. D. Eddy, H. K. Lotze, E. D. Galbraith, W. Cheung, M. Barange, J. L. Blanchard, L. Bopp, A. Bryndum-Buchholz, M. Büchner, C. Bulman, D. A. Carozza, V. Christensen, M. Coll, J. P. Dunne, J. A. Fernandes, E. A. Fulton, A. J. Hobday, V. Huber, S. Jennings, M. Jones, P. Lehodey, J. S. Link, S. MacKinson, O. Maury, S. Niiranen, R. Oliveros-Ramos, T. Roy, J. Schewe, Y. J. Shin, T. Silva, C. A. Stock, J. Steenbeek, P. J. Underwood, J. Volkholz, J. R. Watson, and N. D. Walker. 2018. A protocol for the intercomparison of marine fishery and ecosystem models: Fish-MIP v1.0. Geoscientific Model Development 11:1421–1442.

Tu, C. Y., Y. H. Tseng, T. S. Chiu, M. L. Shen, and C. H. Hsieh. 2012. Using coupled fish behavior-hydrodynamic model to investigate spawning migration of Japanese anchovy, Engraulis japonicus, from the East China Sea to Taiwan. Fisheries Oceanography 21:255–268.

Uriarte, A., P. Alvarez, S. Iversen, J. Molloy, B. Villamor, M. M. Martins, and S. Myklevoll. 2001. Spatial Pattern of Migration and Recruitment of North East Atlantic Mackerel.

van der Vaart, E., M. a. Beaumont, A. S. a. Johnston, and R. M. Sibly. 2015. Calibration and evaluation of individual-based models using Approximate Bayesian Computation. Ecological Modelling 312:182–190.

Villamor, B., P. Abaunza, and C. Farina. 2001. Age and growth of Northeast Atlantic mackerel in waters off the north and northwest of Spain.

Villamor, B., M. Bernal, and C. Hernandez. 2004. Models describing mackerel ( Scomber scombrus ) early life growth in the North and Northwest of the Iberian Peninsula in 2000 *. Scientia Marina 68:571–583.

Wallace, P. D. 1991. Seasonal variation in fat content of mackerel (Scomber scombrus L.) caught in the western English Channel. Fish. Res. Tech. Rep., MAFF Direct. Fish Res., Lowestoft (91): 8 pp:8.

Walsh, M., D. G. Reid, and W. R. Turrell. 1995. Understanding mackerel migration off Scotland: Tracking with echosounders and commercial data, and including environmental correlates and behaviour. ICES Journal of Marine Science 52:925–939.

Watson, J. J., I. G. Priede, P. R. Witthames, and A. Owori-Wadunde. 1992. Batch fecundity of Atlantic mackerel, Scomber scombrus L. J Fish Biol:591–598.

Wilhelms, I. 2013. Thünen Working Paper 12: Atlas of length-weight relationships of 93 fish and crustacean species from the North Sea and the North-East Atlantic.

Winters, G. H. 1976. Recruitment Mechanisms of Southern Gulf of St Lawrence Atlantic Herring (Clupea harengus harengus). J Fish Res.Board.Can 33:1751–1763.

Worley, P. 1933. Development of the egg of the mackerel at different constant temperatures. Journal of General Physiology:841–857.

Yamada, T., I. Aoki, and I. Mitani. 1998. Spawning time, spawning frequency and fecundity of Japanese chub mackerel, Scomber japonicus in the waters around the Izu Islands, Japan. Fisheries Research 38:83–89.

Yohannan, T. M. 1979. The growth pattern of Indian mackerel. Indian Journal of Fisheries 26.
